# Supplementary figures and images for: The nanoscale organization of the Nipah virus fusion protein informs new membrane fusion mechanisms
Source: eLife. 2025 Jan 2;13:RP97017. doi: 10.7554/eLife.97017 (PMC11695058; doi:10.7554/eLife.97017)

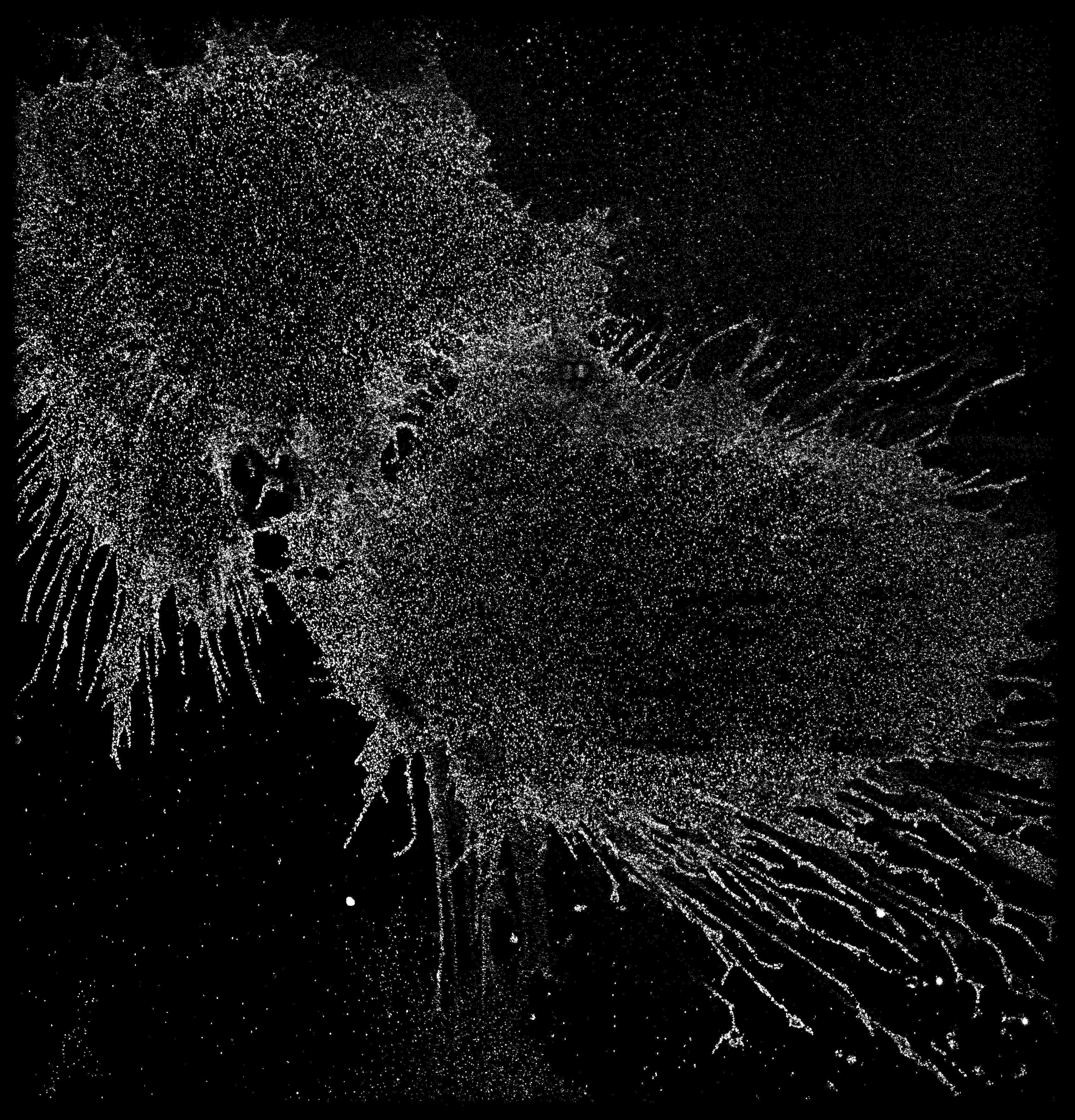

Supplement: Figure 1—source data 1. [file elife-97017-fig1-data1.jpg]

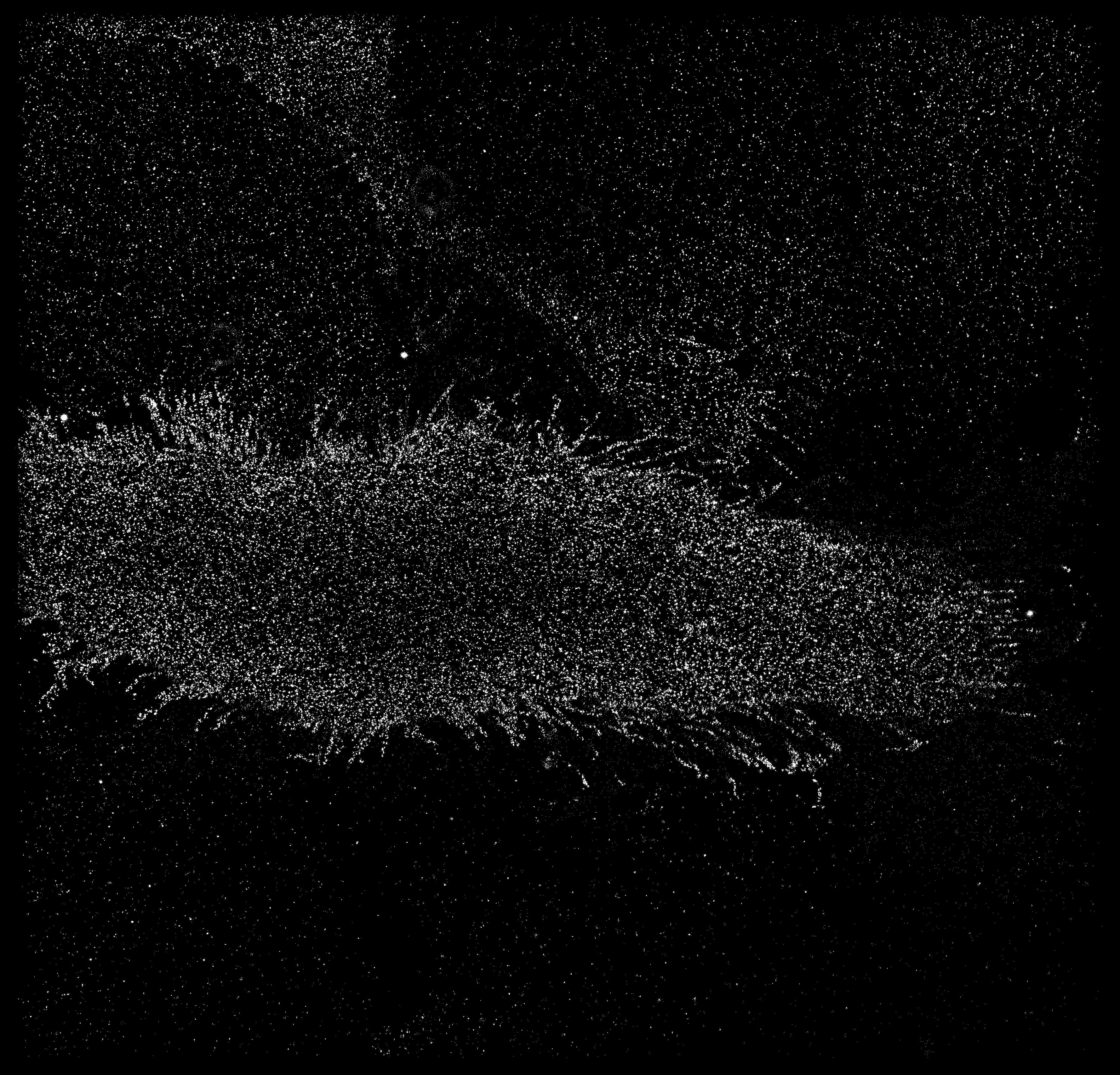

Supplement: Figure 1—source data 2. [file elife-97017-fig1-data2.jpg]

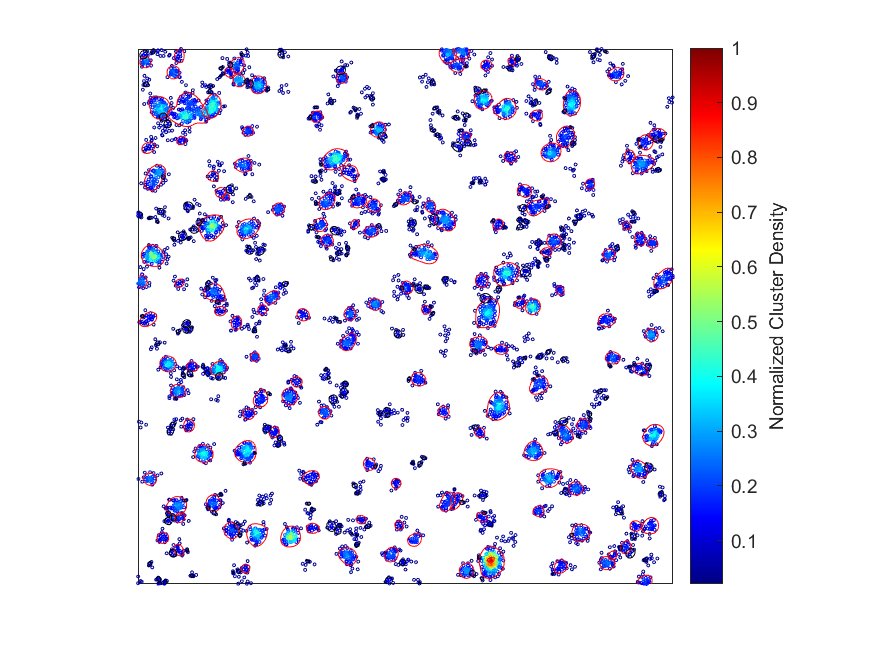

Supplement: Figure 1—source data 3. [file elife-97017-fig1-data3.zip › Figure 1_source data 3_Related to Figure 1D/Density map.jpg]

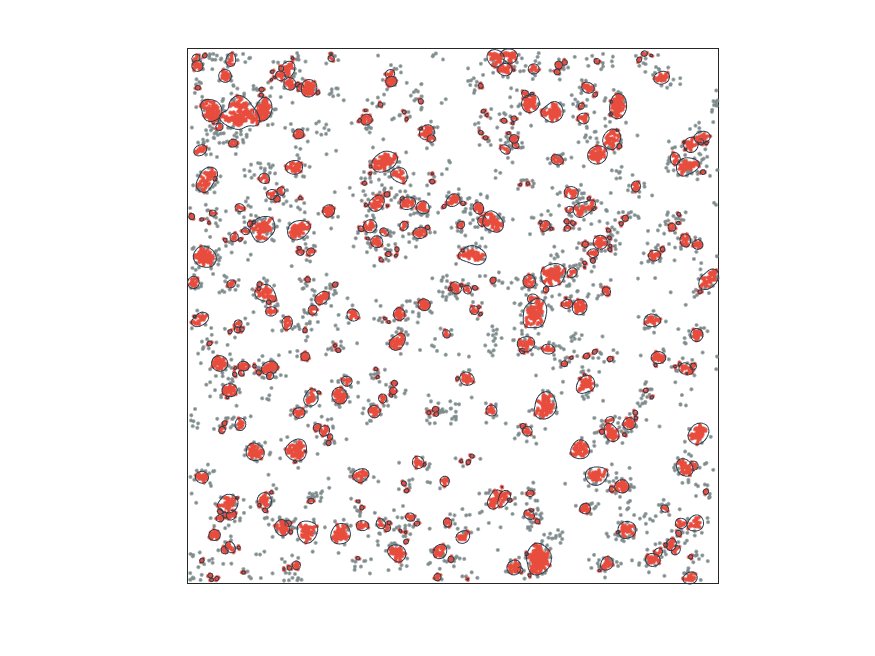

Supplement: Figure 1—source data 3. [file elife-97017-fig1-data3.zip › Figure 1_source data 3_Related to Figure 1D/Cluster map.jpg]

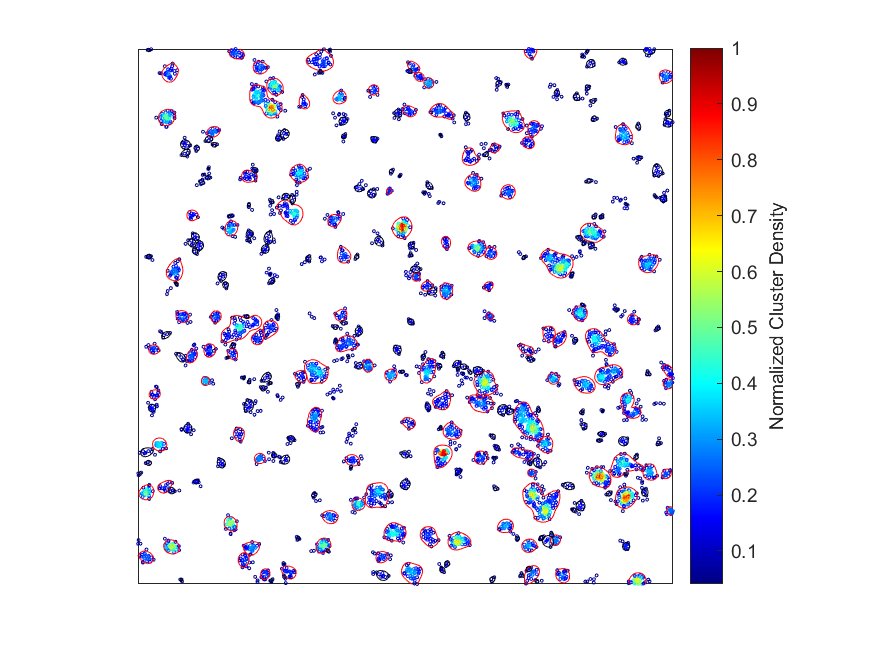

Supplement: Figure 1—source data 4. [file elife-97017-fig1-data4.zip › Figure 1_source data 4_Related to Figure 1E/Density map.jpg]

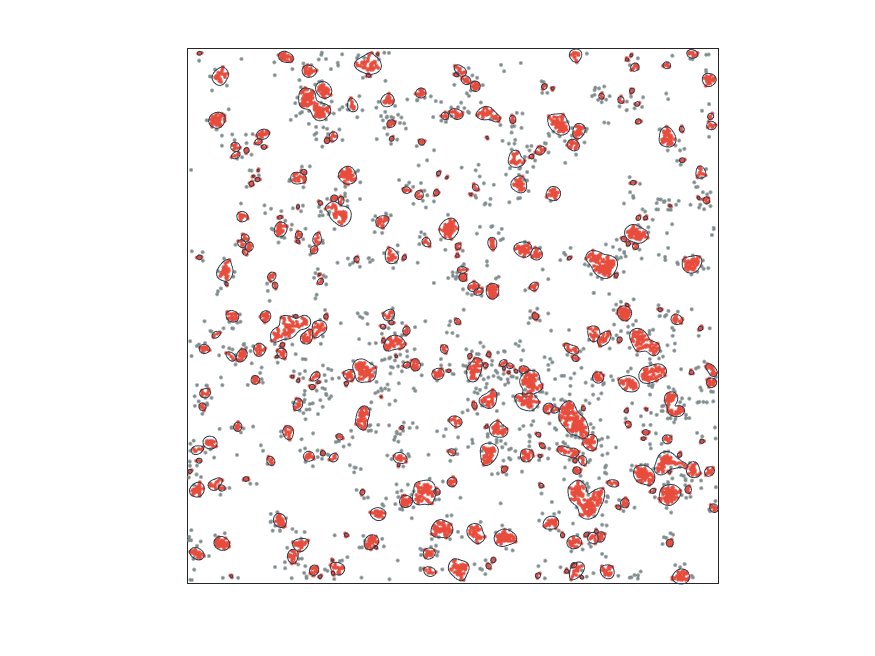

Supplement: Figure 1—source data 4. [file elife-97017-fig1-data4.zip › Figure 1_source data 4_Related to Figure 1E/Cluster map.jpg]

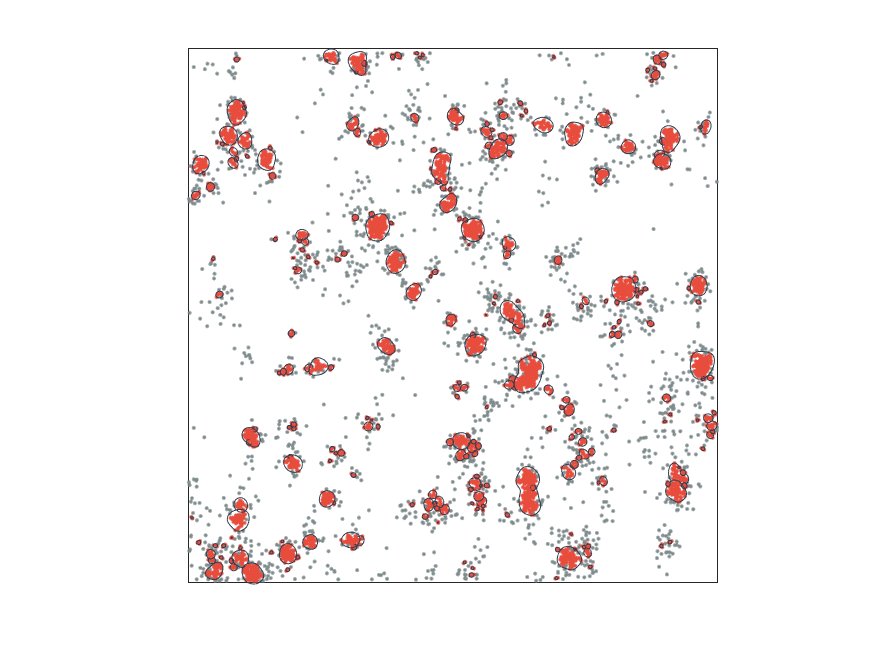

Supplement: Figure 1—figure supplement 1—source data 1. [file elife-97017-fig1-figsupp1-data1.zip › Figure 1-figure supplement 1_source data1/HA-ClusterMap.jpg]

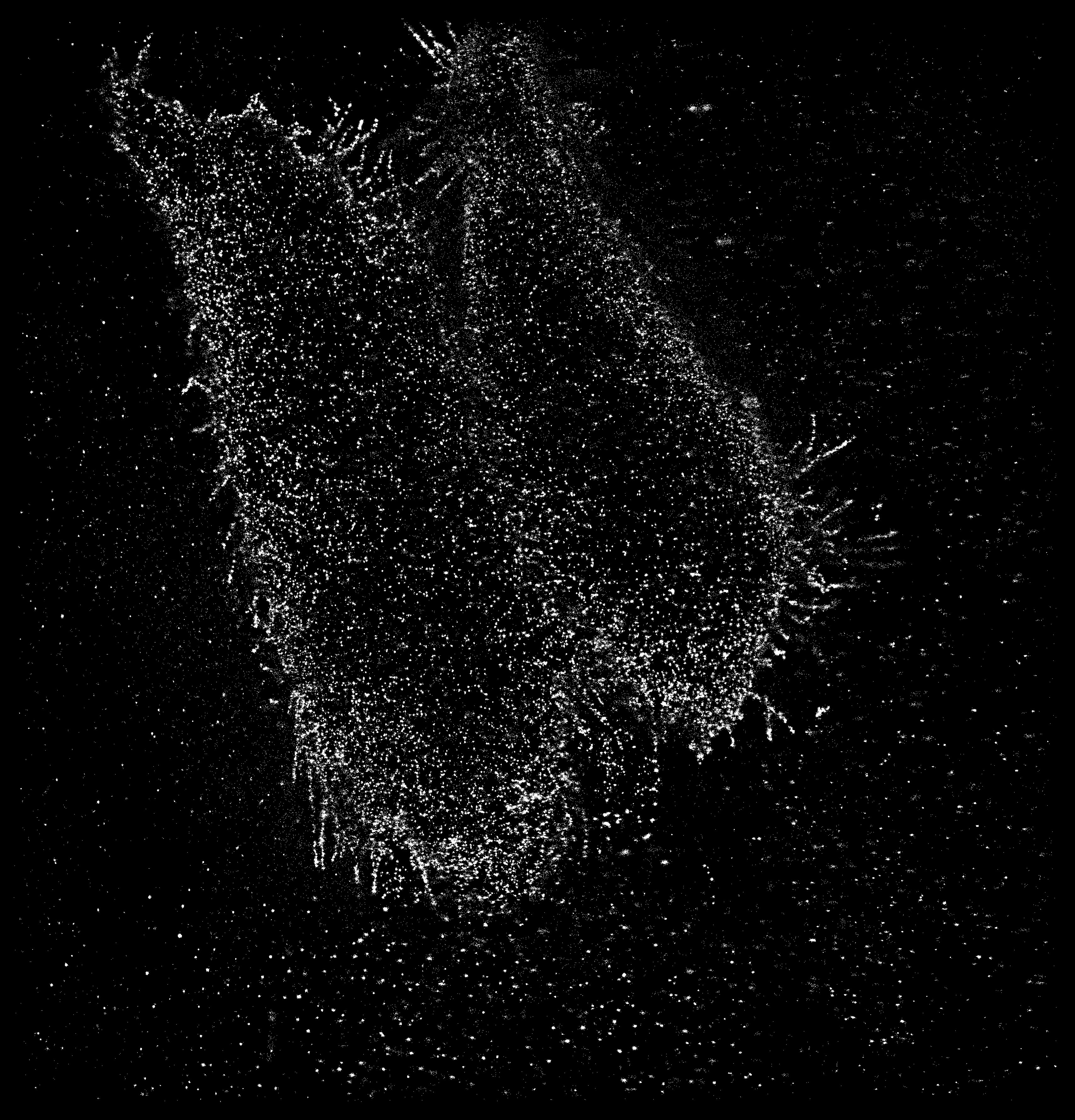

Supplement: Figure 1—figure supplement 1—source data 1. [file elife-97017-fig1-figsupp1-data1.zip › Figure 1-figure supplement 1_source data1/NiV-F-HA.jpg]

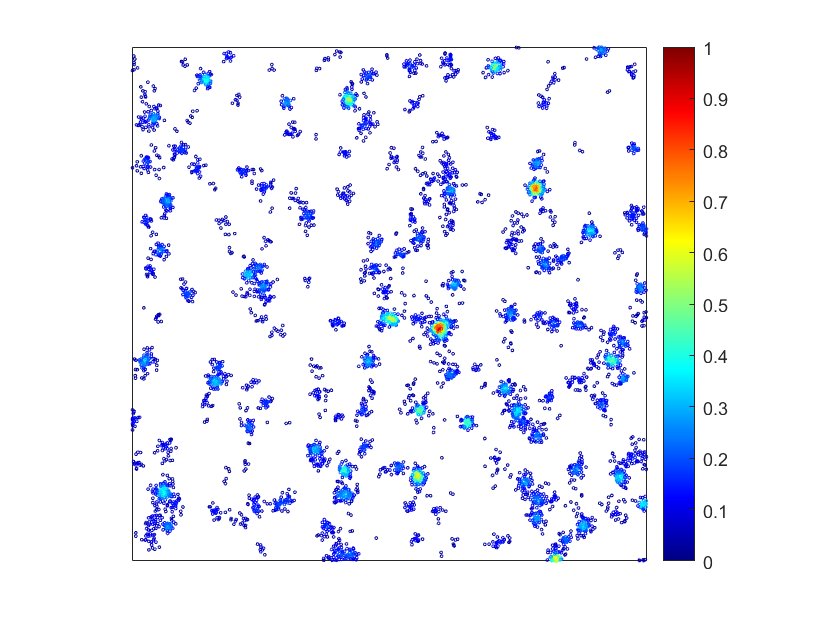

Supplement: Figure 1—figure supplement 1—source data 1. [file elife-97017-fig1-figsupp1-data1.zip › Figure 1-figure supplement 1_source data1/FLAG-DensityMap.jpg]

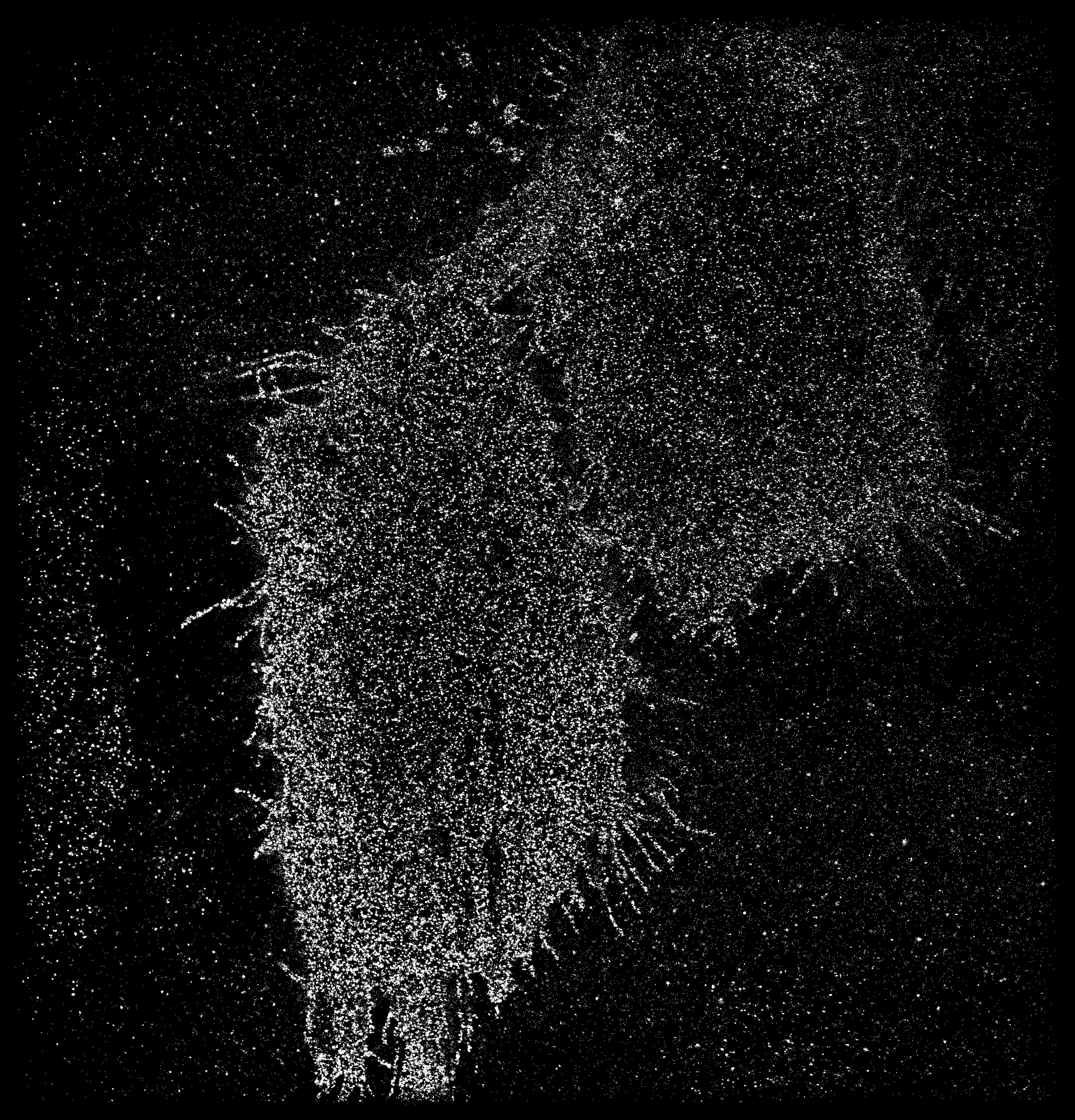

Supplement: Figure 1—figure supplement 1—source data 1. [file elife-97017-fig1-figsupp1-data1.zip › Figure 1-figure supplement 1_source data1/NiV-F-FLAG.jpg]

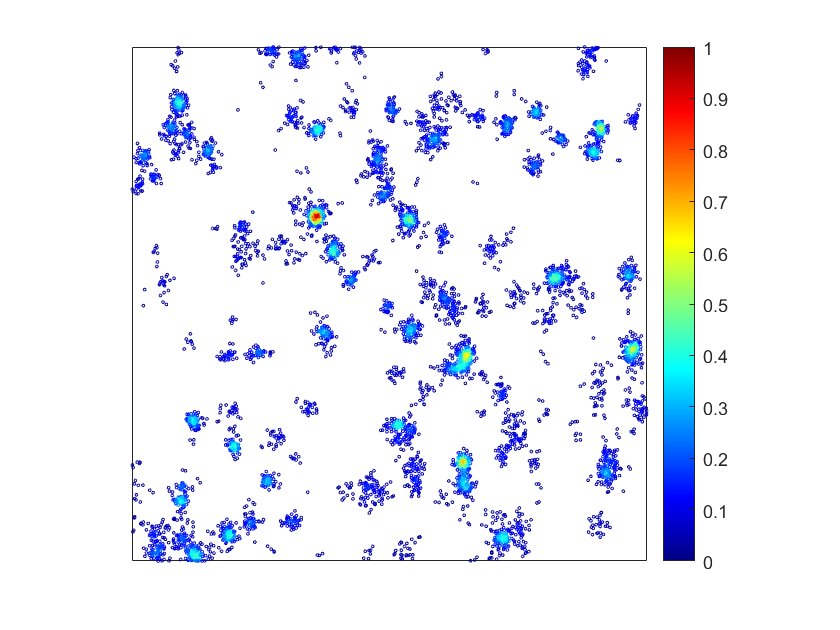

Supplement: Figure 1—figure supplement 1—source data 1. [file elife-97017-fig1-figsupp1-data1.zip › Figure 1-figure supplement 1_source data1/HA-DensityMap.jpg]

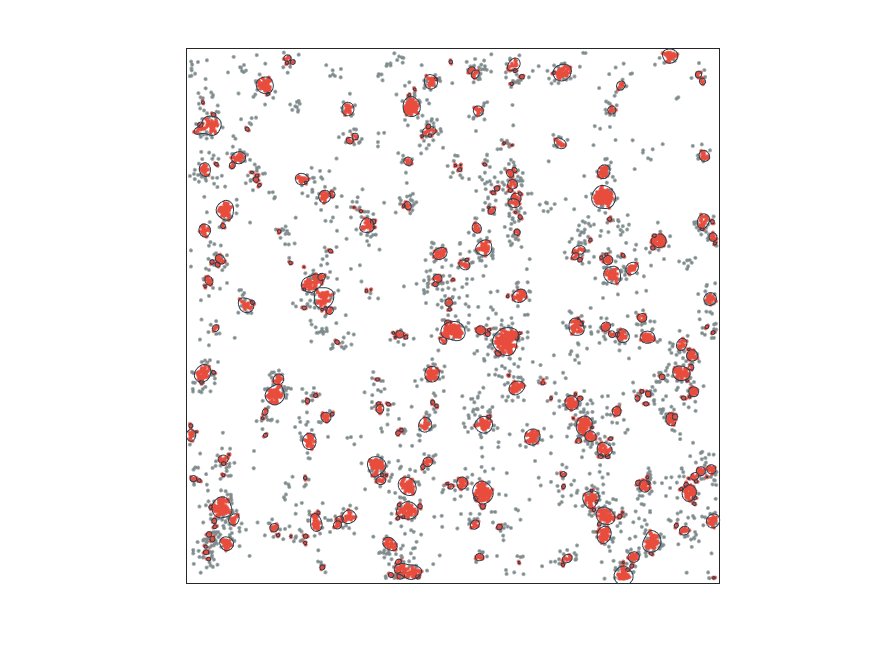

Supplement: Figure 1—figure supplement 1—source data 1. [file elife-97017-fig1-figsupp1-data1.zip › Figure 1-figure supplement 1_source data1/FLAG-ClusterMap.jpg]

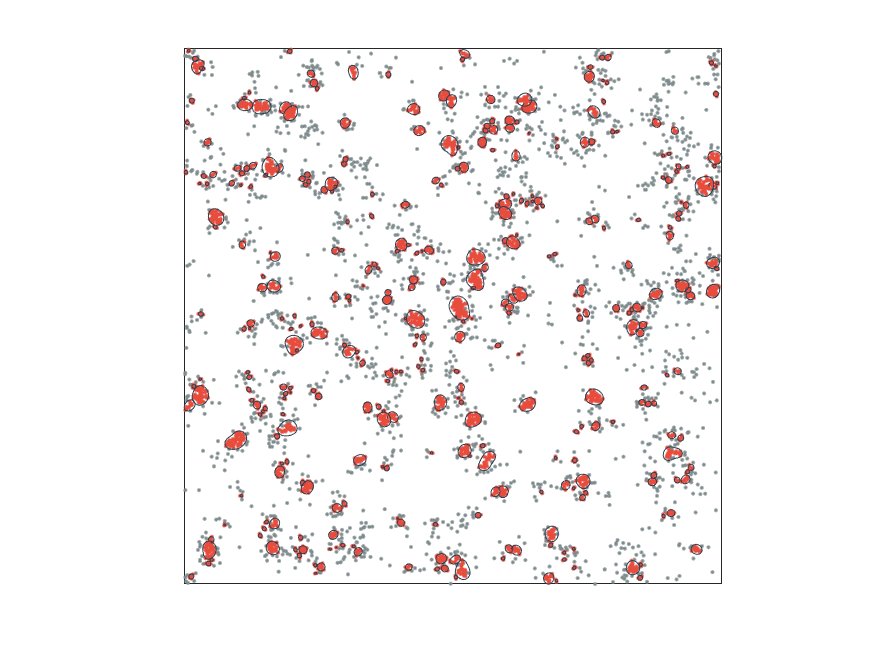

Supplement: Figure 1—figure supplement 2—source data 1. [file elife-97017-fig1-figsupp2-data1.zip › Figure 1-figure supplement 2_source data1/Hela-ClusterMap.jpg]

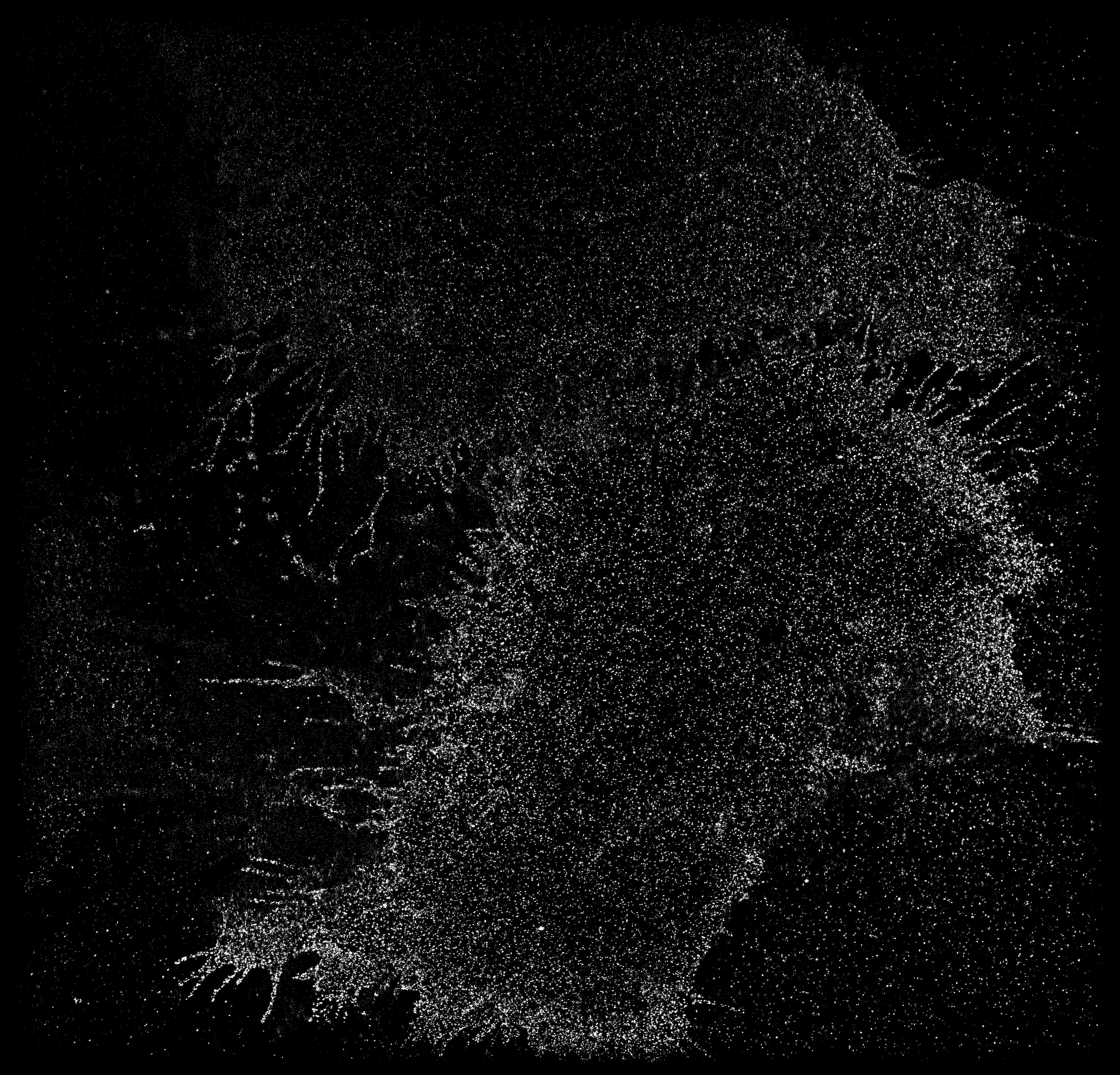

Supplement: Figure 1—figure supplement 2—source data 1. [file elife-97017-fig1-figsupp2-data1.zip › Figure 1-figure supplement 2_source data1/PK13.jpg]

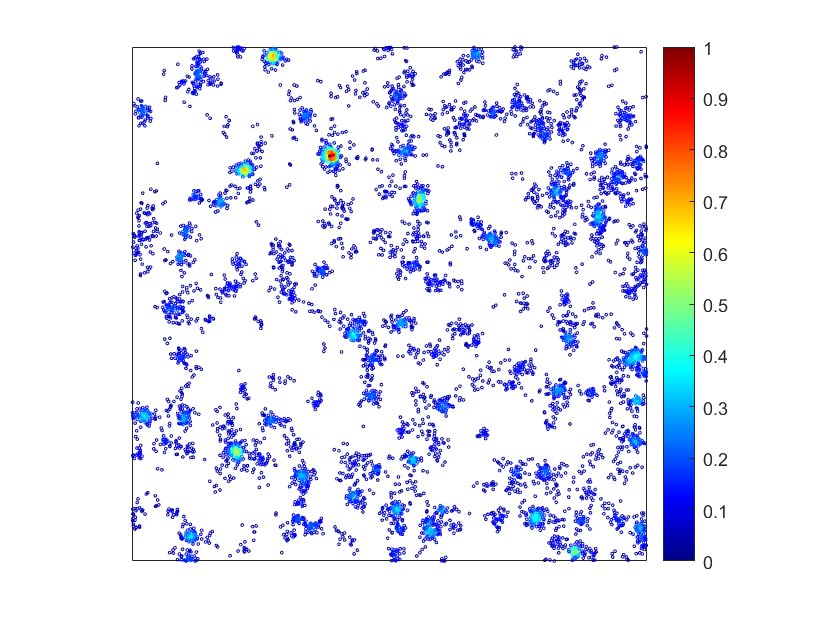

Supplement: Figure 1—figure supplement 2—source data 1. [file elife-97017-fig1-figsupp2-data1.zip › Figure 1-figure supplement 2_source data1/PK13-DensityMap.jpg]

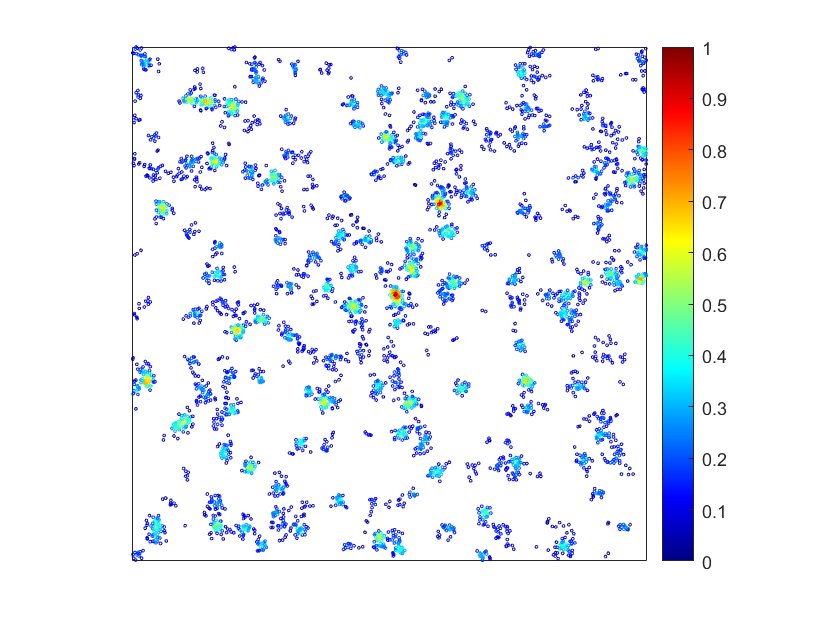

Supplement: Figure 1—figure supplement 2—source data 1. [file elife-97017-fig1-figsupp2-data1.zip › Figure 1-figure supplement 2_source data1/HELA-DensityMap.jpg]

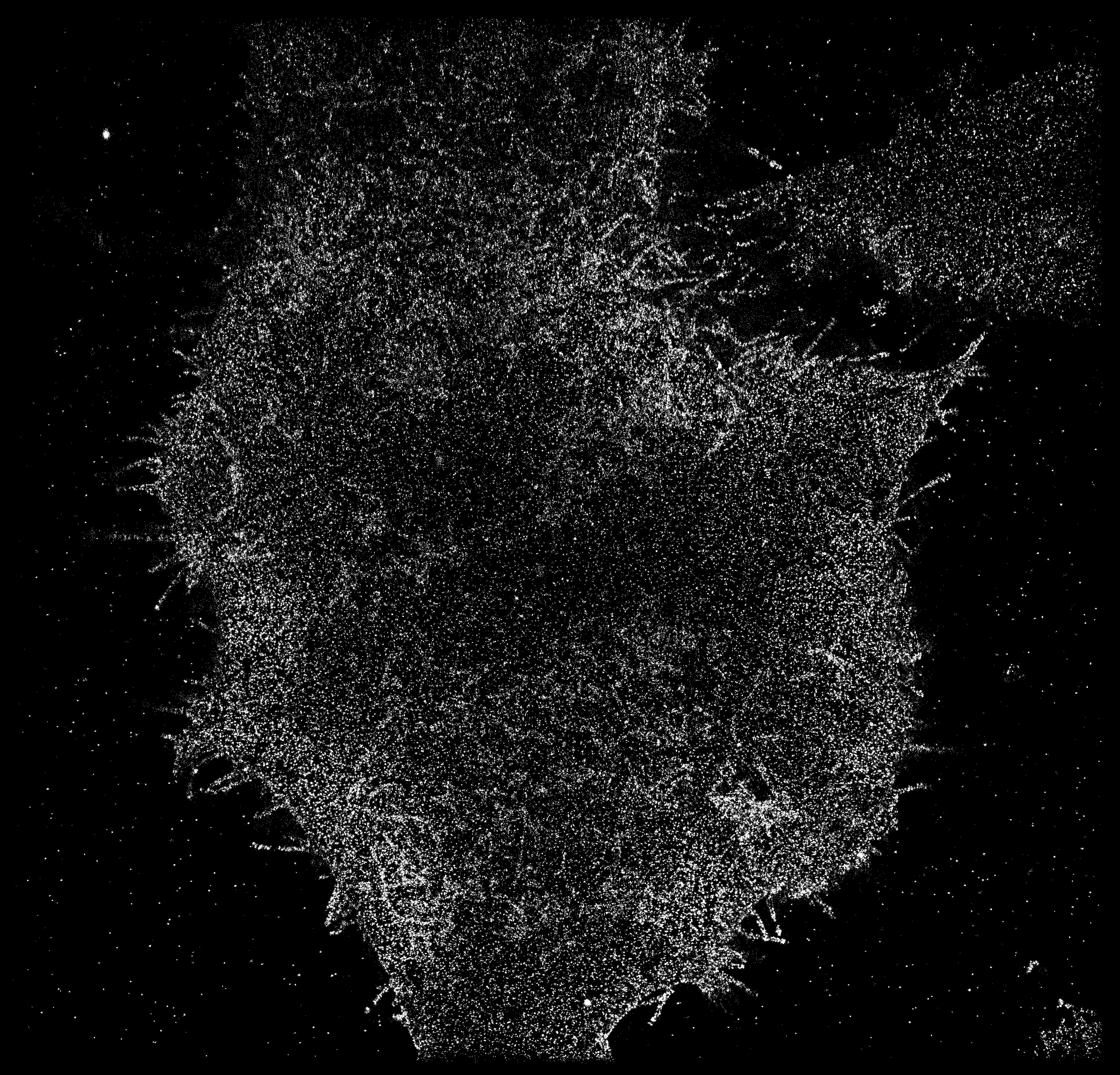

Supplement: Figure 1—figure supplement 2—source data 1. [file elife-97017-fig1-figsupp2-data1.zip › Figure 1-figure supplement 2_source data1/HELA.jpg]

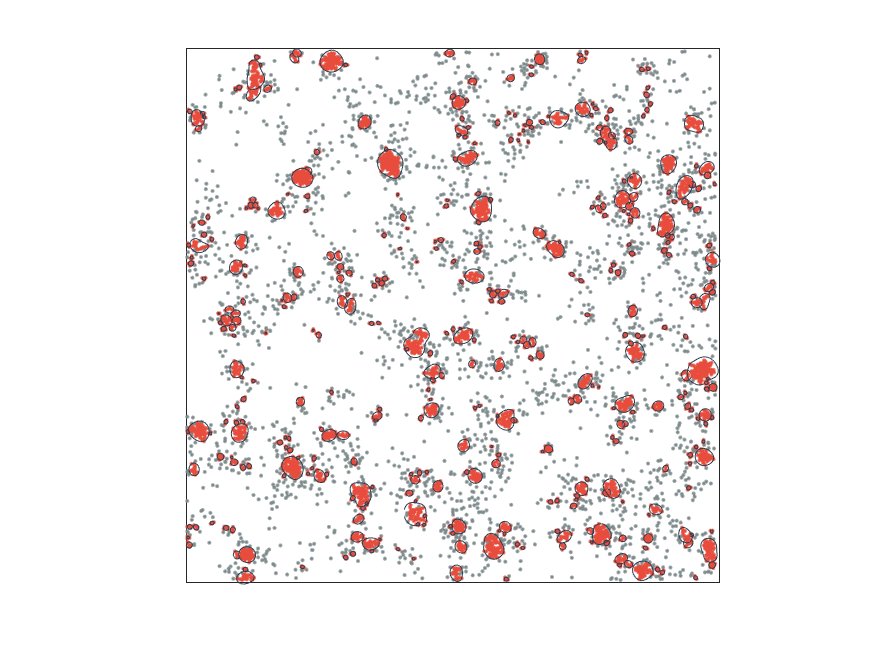

Supplement: Figure 1—figure supplement 2—source data 1. [file elife-97017-fig1-figsupp2-data1.zip › Figure 1-figure supplement 2_source data1/PK13-ClusterMap.jpg]

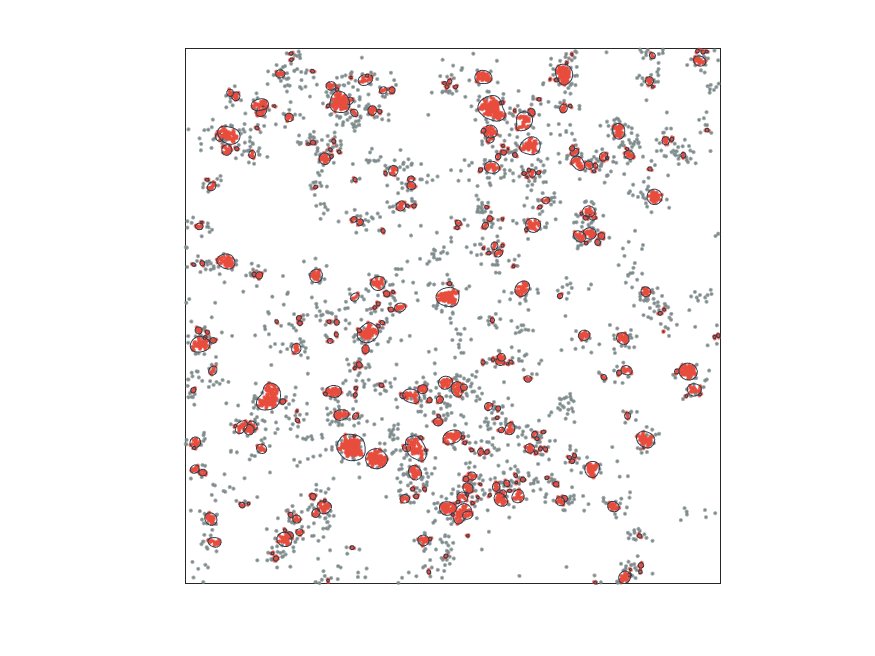

Supplement: Figure 2—source data 1. [file elife-97017-fig2-data1.zip › Figure 2_source data 1/E64d-ClusterMap.jpg]

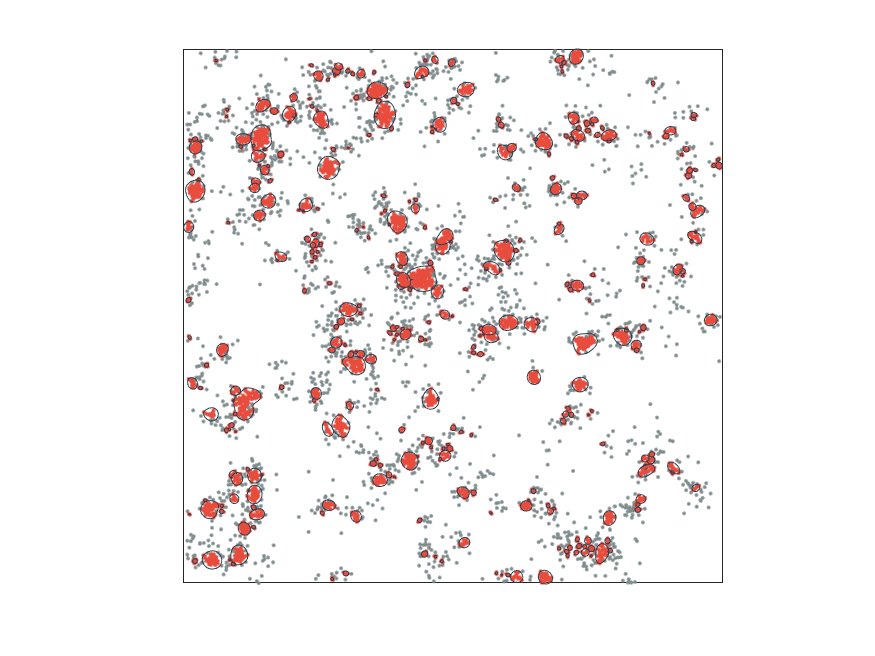

Supplement: Figure 2—source data 1. [file elife-97017-fig2-data1.zip › Figure 2_source data 1/NC-ClusterMap.jpg]

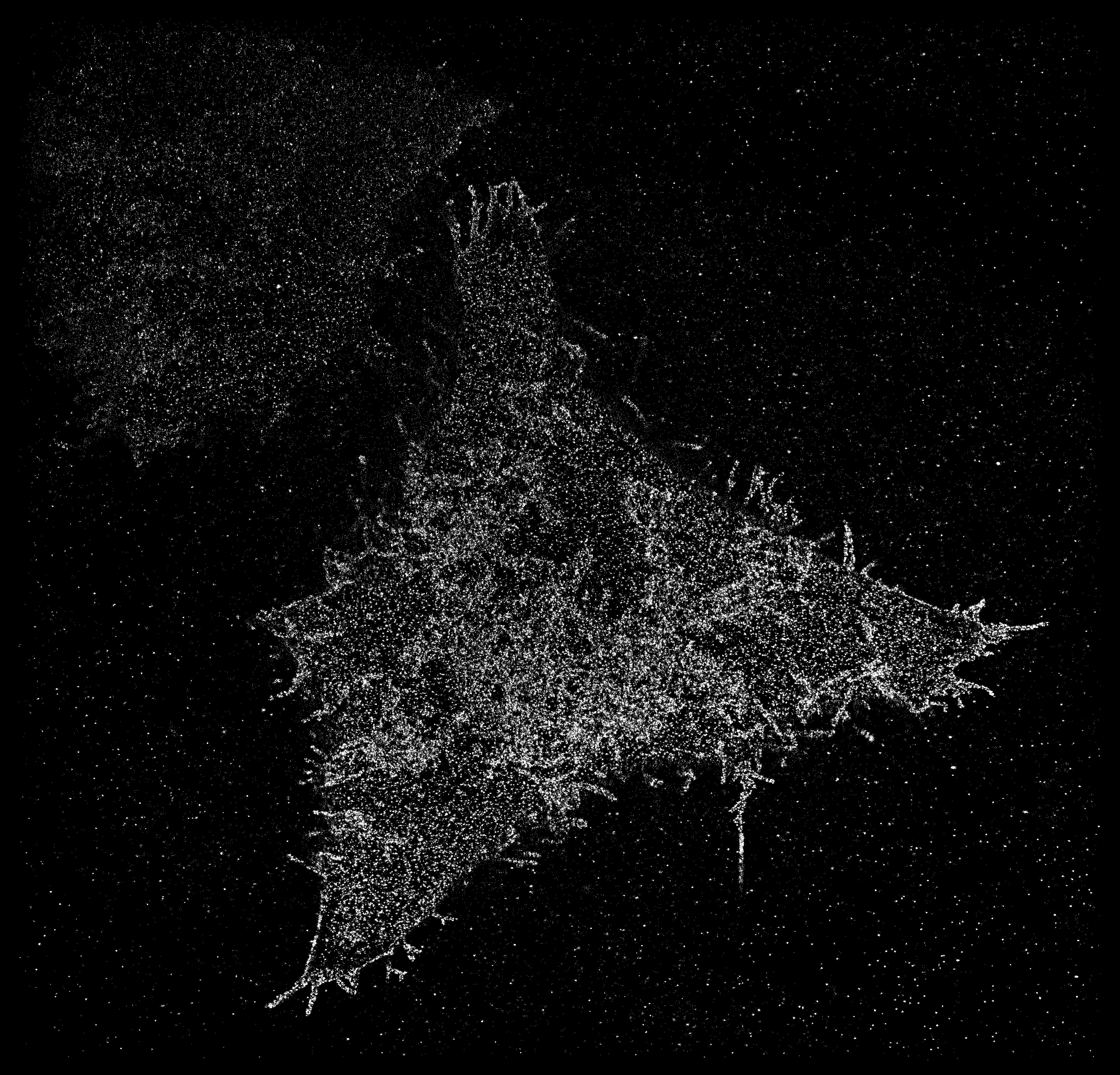

Supplement: Figure 2—source data 1. [file elife-97017-fig2-data1.zip › Figure 2_source data 1/E64d.jpg]

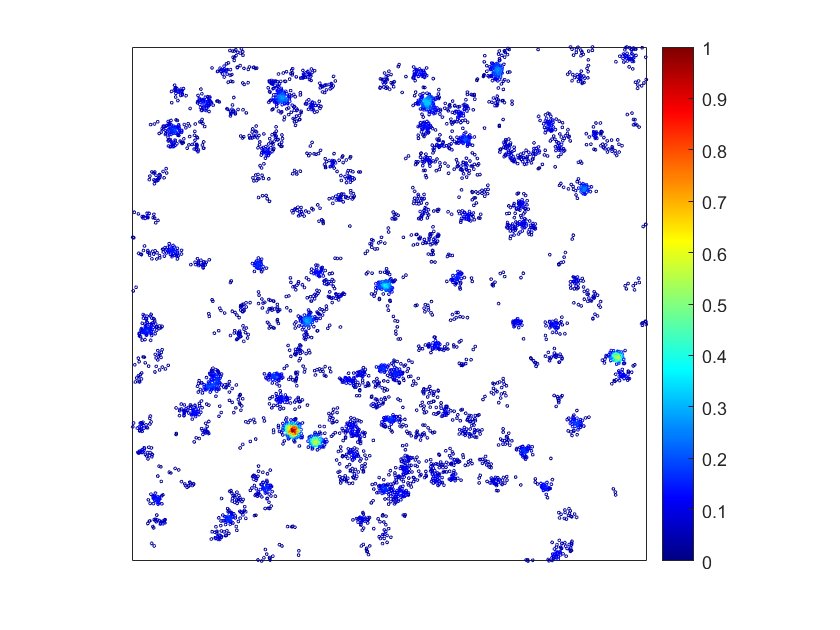

Supplement: Figure 2—source data 1. [file elife-97017-fig2-data1.zip › Figure 2_source data 1/E64d-DensityMap.jpg]

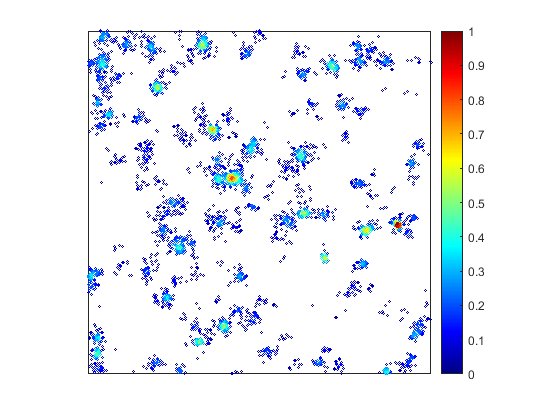

Supplement: Figure 2—source data 1. [file elife-97017-fig2-data1.zip › Figure 2_source data 1/NC-DensityMap.jpg]

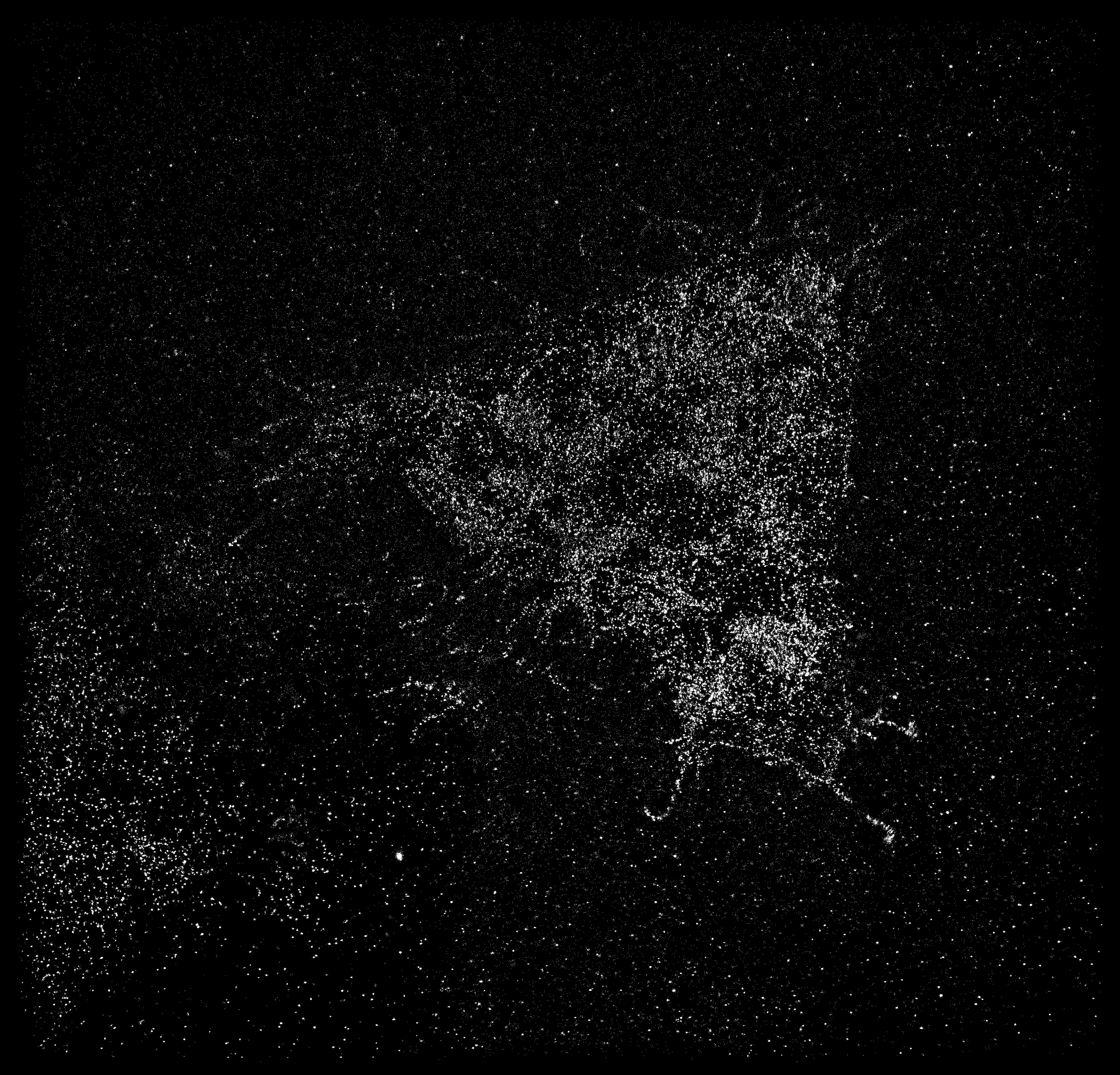

Supplement: Figure 2—source data 1. [file elife-97017-fig2-data1.zip › Figure 2_source data 1/NC.jpg]

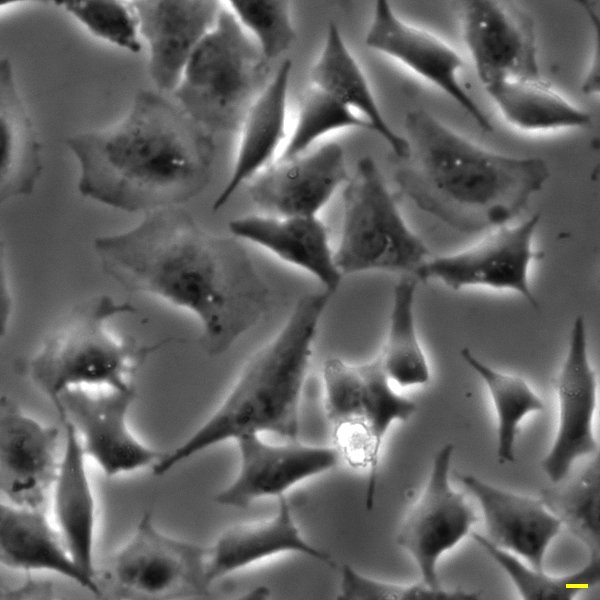

Supplement: Figure 2—figure supplement 1—source data 1. [file elife-97017-fig2-figsupp1-data1.zip › Figure 2-figure supplement 1_source data 1/E64d-fusion.jpg]

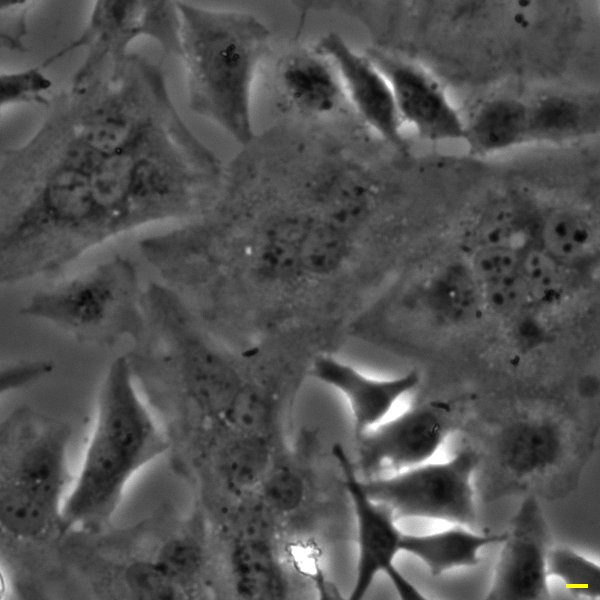

Supplement: Figure 2—figure supplement 1—source data 1. [file elife-97017-fig2-figsupp1-data1.zip › Figure 2-figure supplement 1_source data 1/NC-fusion.jpg]

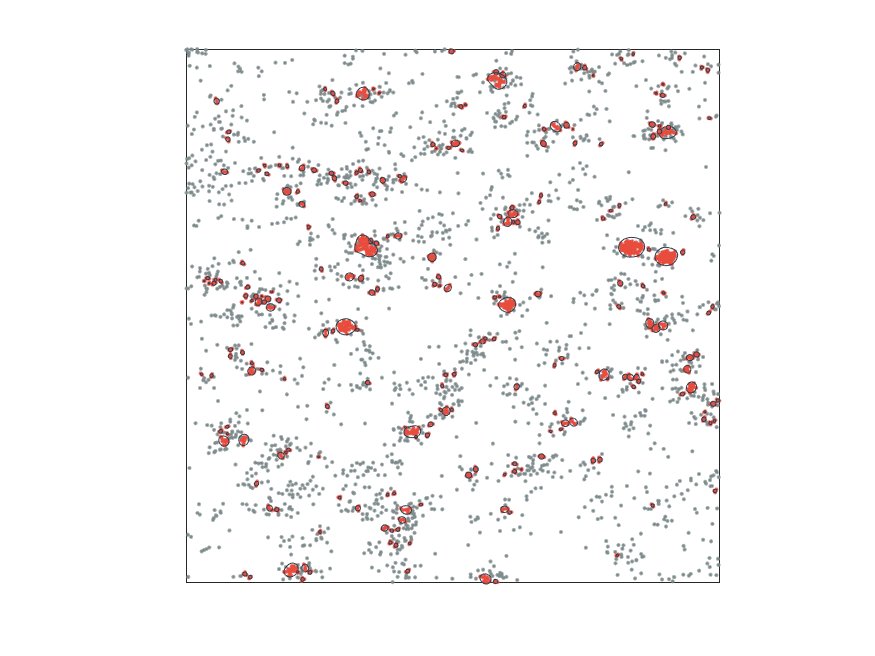

Supplement: Figure 3—source data 1. [file elife-97017-fig3-data1.zip › Figure 3_source data 1/L53D-ClusterMap.jpg]

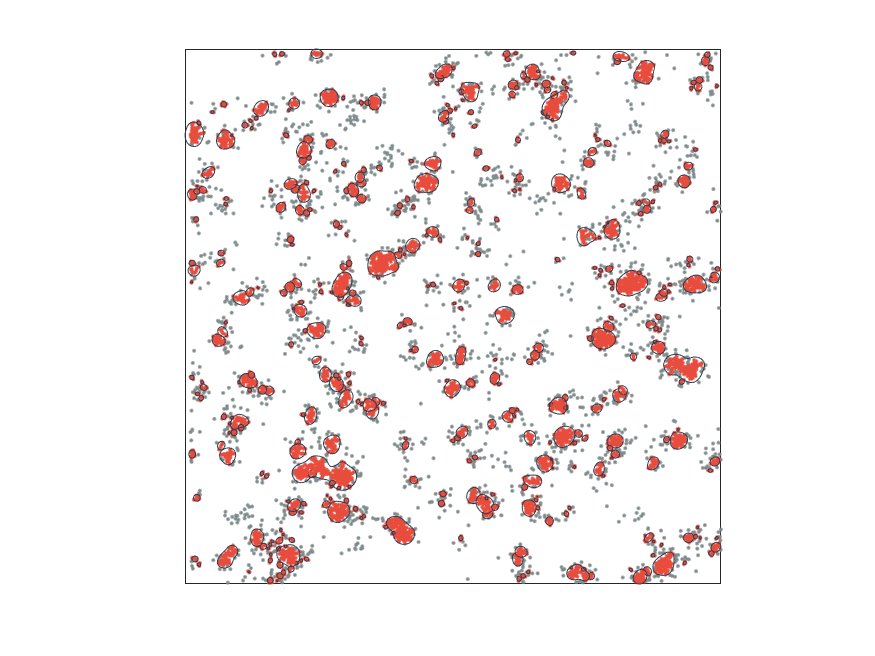

Supplement: Figure 3—source data 1. [file elife-97017-fig3-data1.zip › Figure 3_source data 1/WT-ClusterMap.jpg]

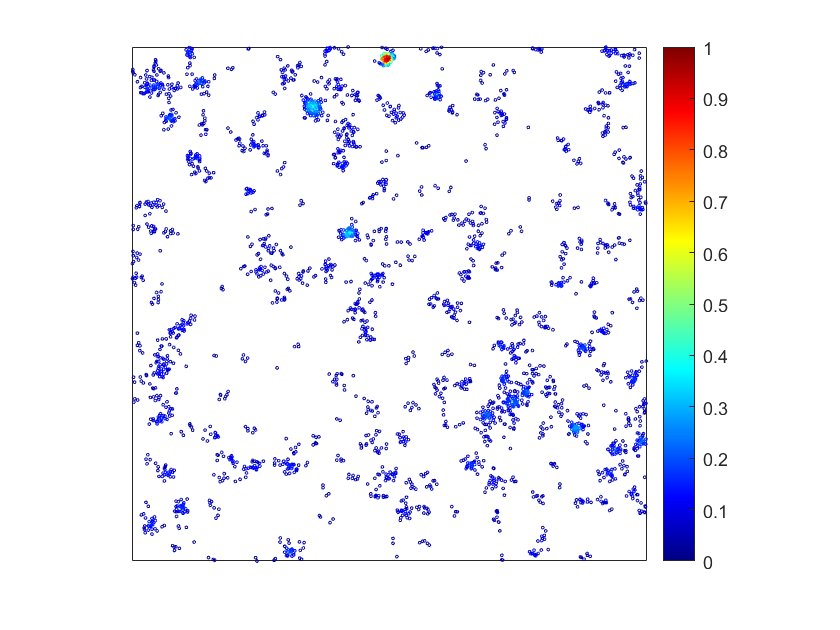

Supplement: Figure 3—source data 1. [file elife-97017-fig3-data1.zip › Figure 3_source data 1/V108D-DensityMap.jpg]

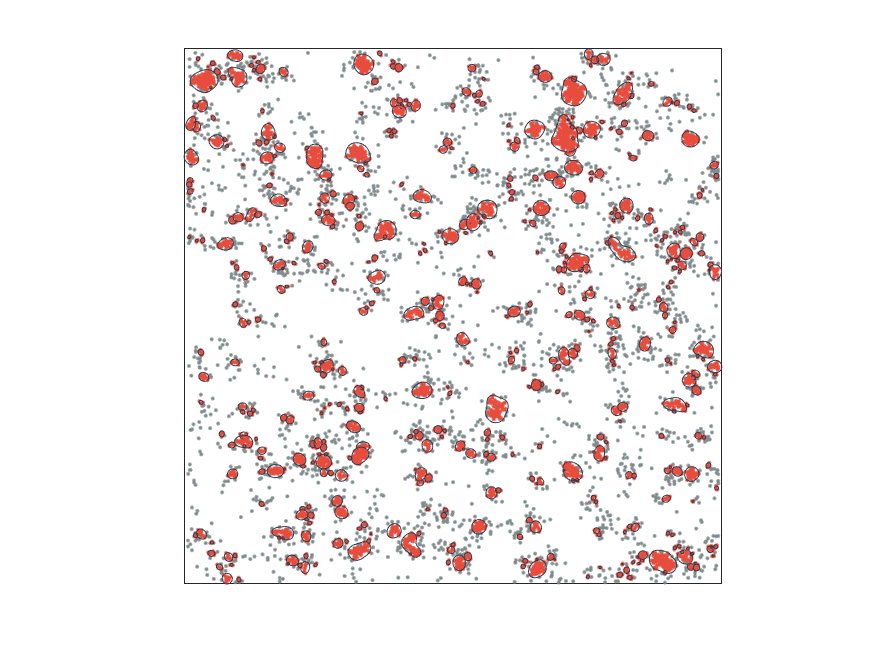

Supplement: Figure 3—source data 1. [file elife-97017-fig3-data1.zip › Figure 3_source data 1/Q393L-ClusterMap.jpg]

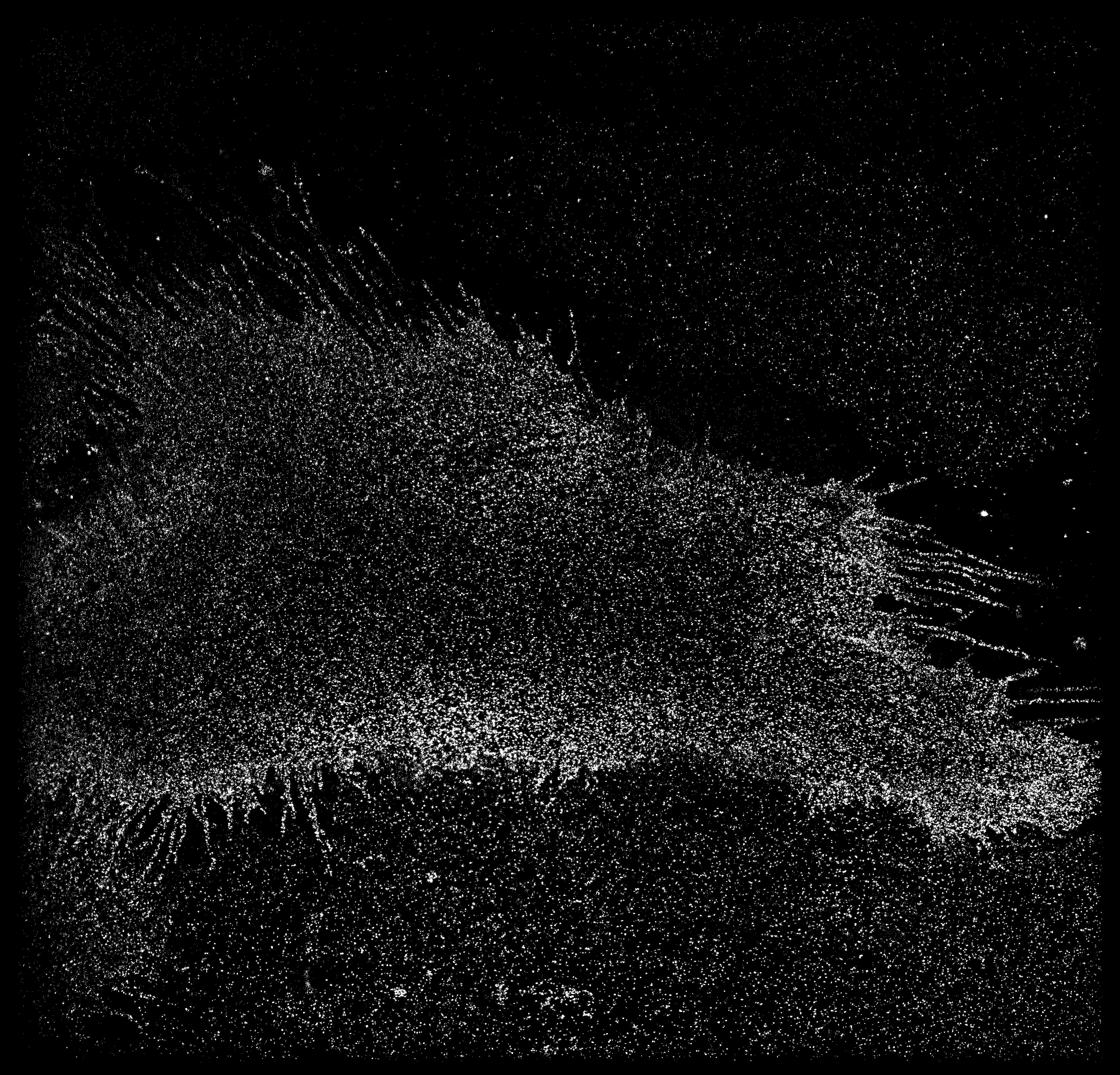

Supplement: Figure 3—source data 1. [file elife-97017-fig3-data1.zip › Figure 3_source data 1/Q393L.jpg]

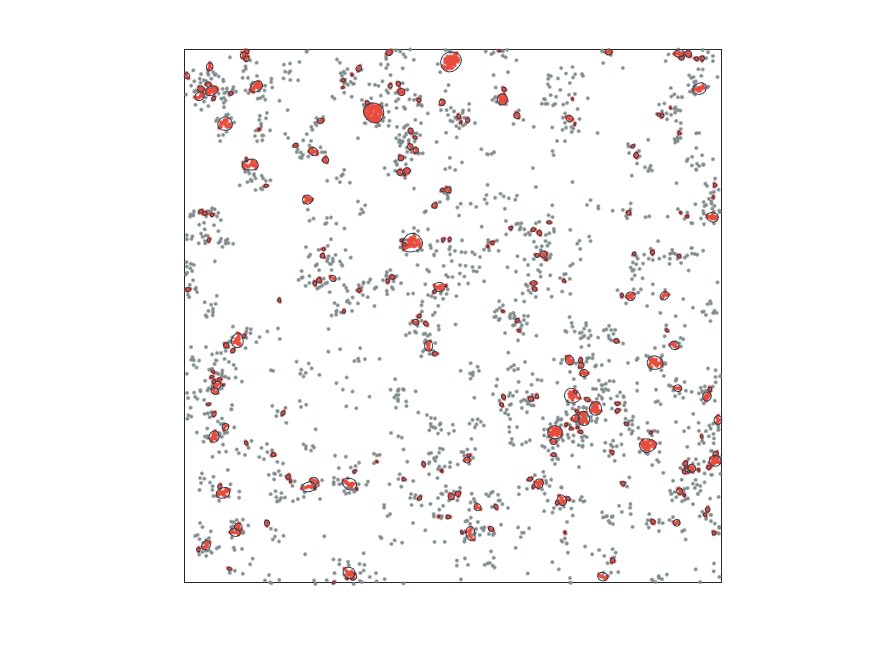

Supplement: Figure 3—source data 1. [file elife-97017-fig3-data1.zip › Figure 3_source data 1/V108D-ClusterMap.jpg]

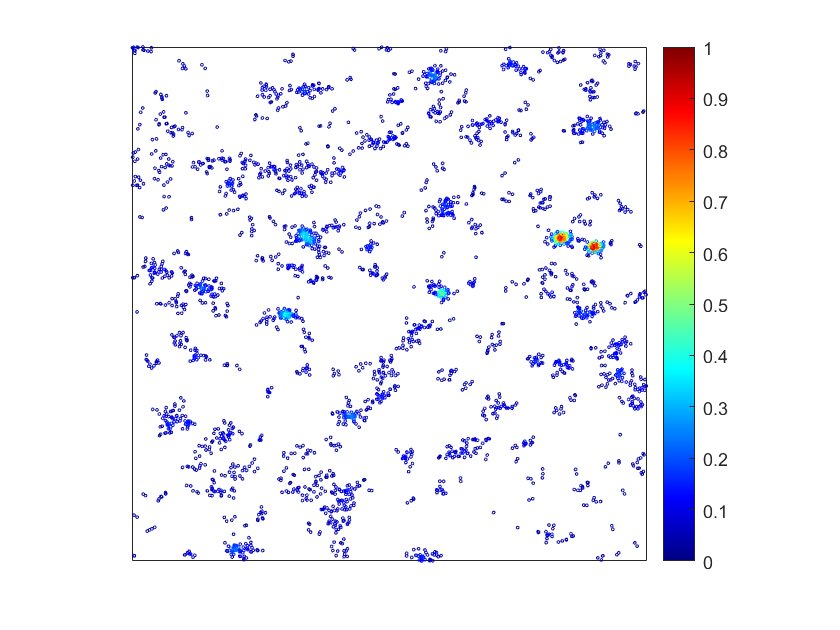

Supplement: Figure 3—source data 1. [file elife-97017-fig3-data1.zip › Figure 3_source data 1/L53D-DensityMap.jpg]

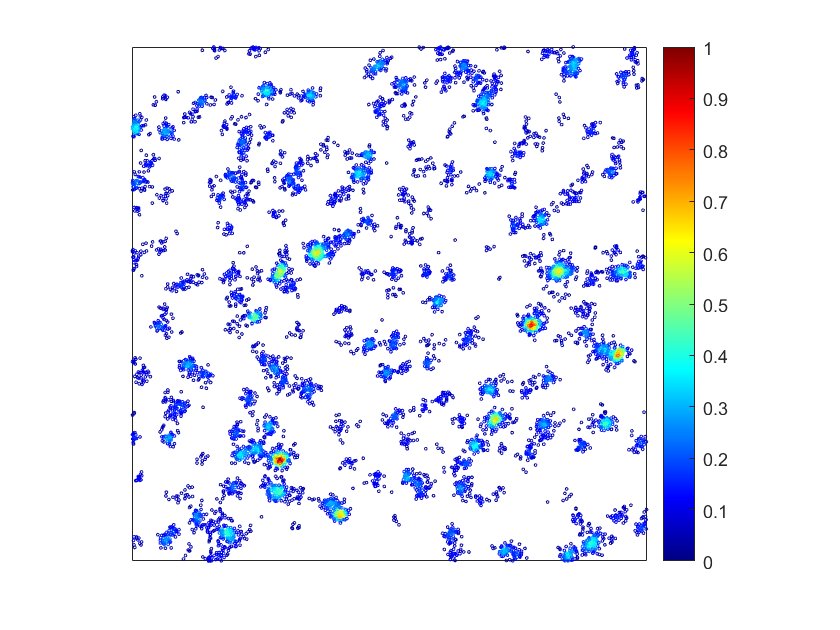

Supplement: Figure 3—source data 1. [file elife-97017-fig3-data1.zip › Figure 3_source data 1/WT-DensityMap.jpg]

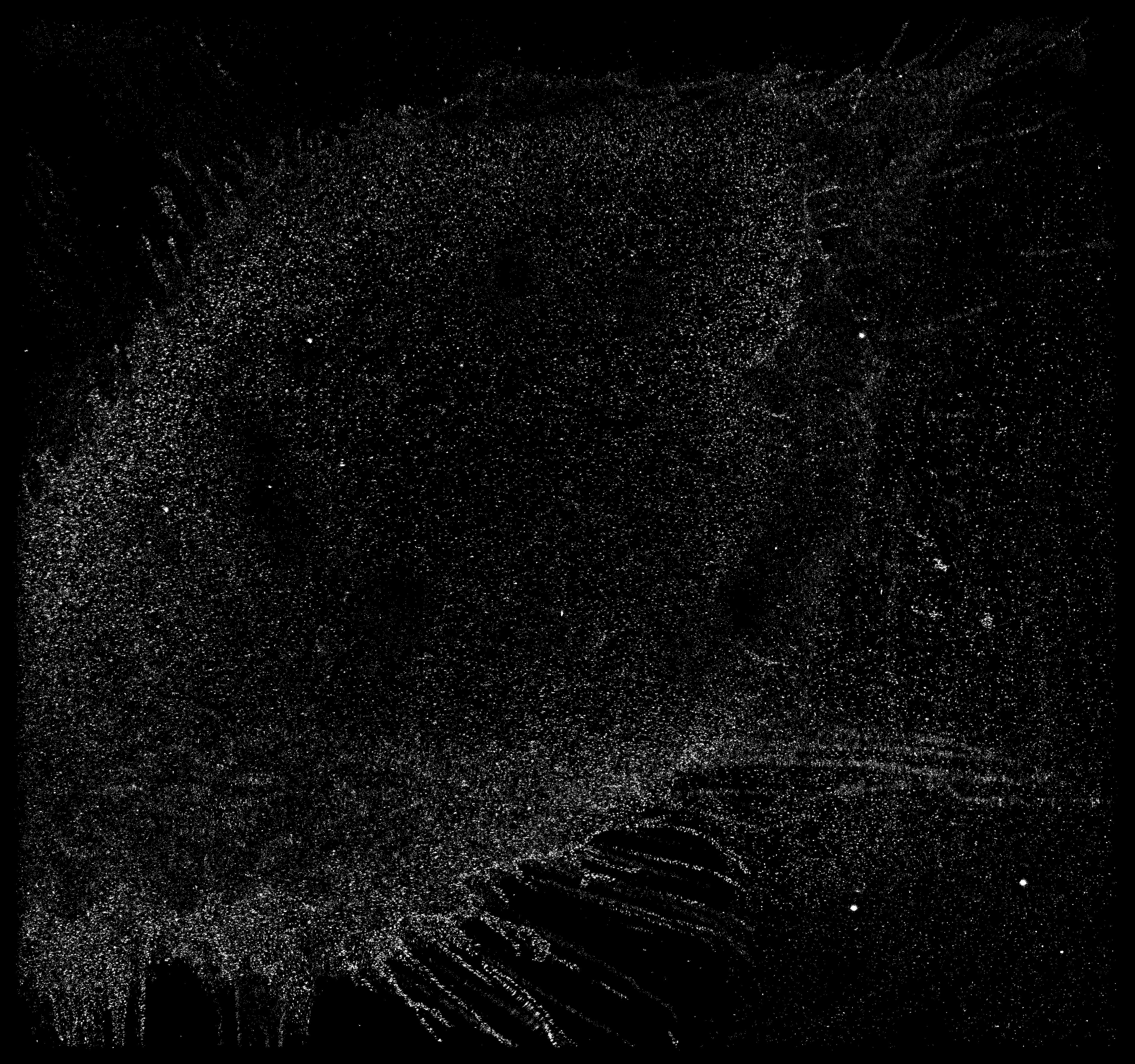

Supplement: Figure 3—source data 1. [file elife-97017-fig3-data1.zip › Figure 3_source data 1/L53D.jpg]

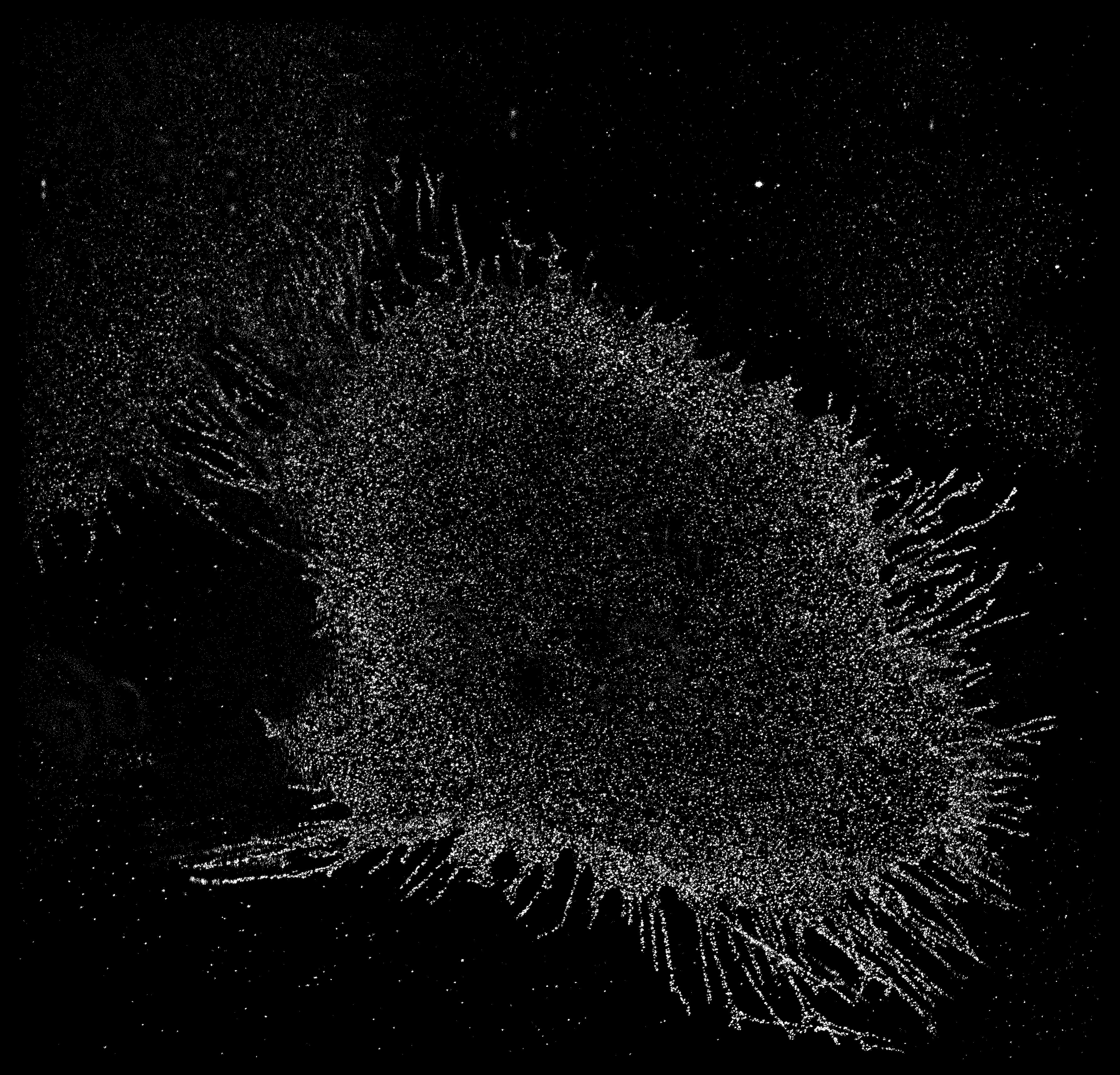

Supplement: Figure 3—source data 1. [file elife-97017-fig3-data1.zip › Figure 3_source data 1/WT.jpg]

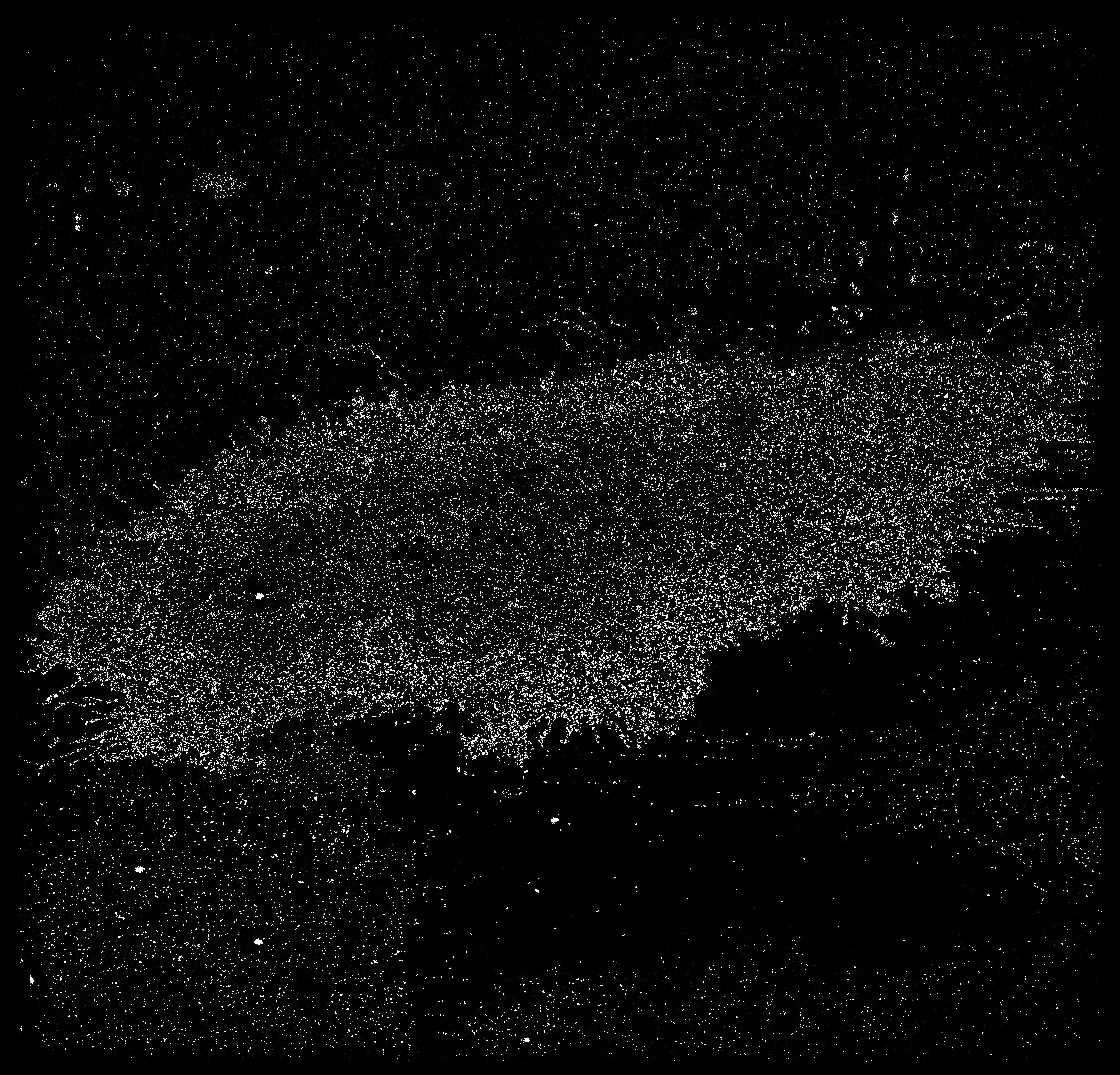

Supplement: Figure 3—source data 1. [file elife-97017-fig3-data1.zip › Figure 3_source data 1/V108D.jpg]

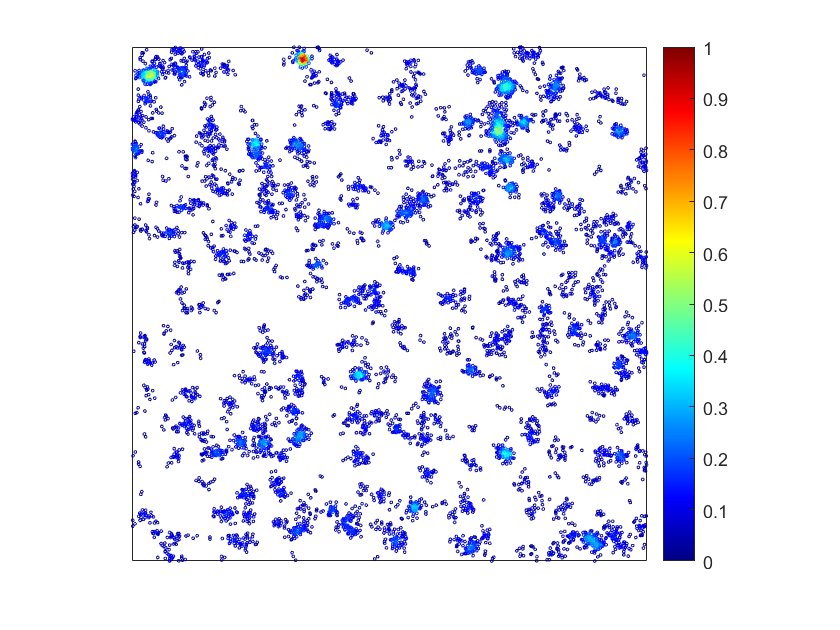

Supplement: Figure 3—source data 1. [file elife-97017-fig3-data1.zip › Figure 3_source data 1/Q393L-DensityMap.jpg]

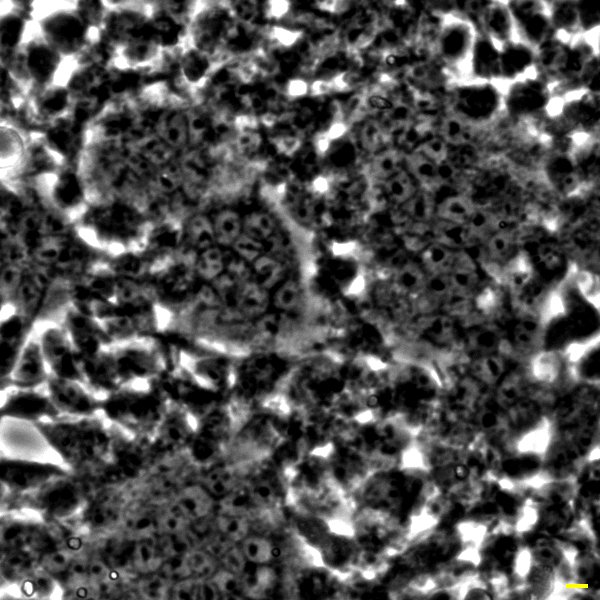

Supplement: Figure 3—figure supplement 1—source data 1. [file elife-97017-fig3-figsupp1-data1.zip › Figure 3-figure supplement 1_source data 1/Q393L.jpg]

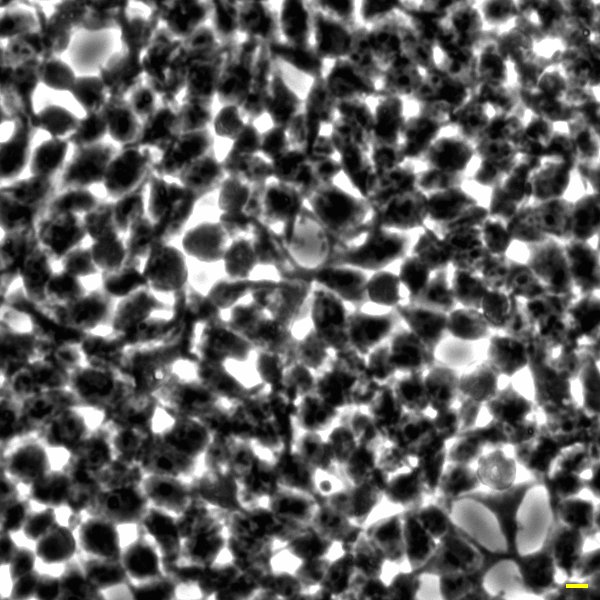

Supplement: Figure 3—figure supplement 1—source data 1. [file elife-97017-fig3-figsupp1-data1.zip › Figure 3-figure supplement 1_source data 1/L53D.jpg]

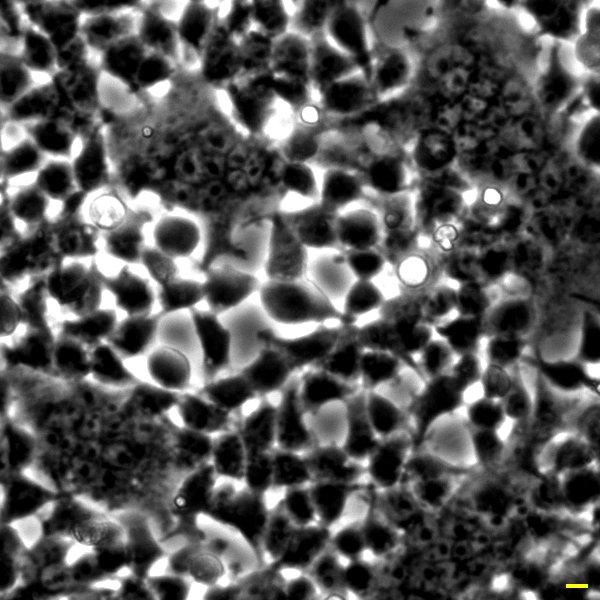

Supplement: Figure 3—figure supplement 1—source data 1. [file elife-97017-fig3-figsupp1-data1.zip › Figure 3-figure supplement 1_source data 1/WT.jpg]

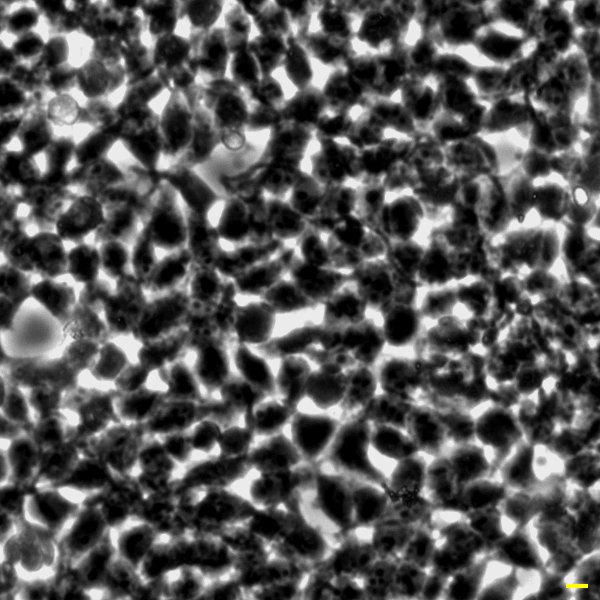

Supplement: Figure 3—figure supplement 1—source data 1. [file elife-97017-fig3-figsupp1-data1.zip › Figure 3-figure supplement 1_source data 1/NC.jpg]

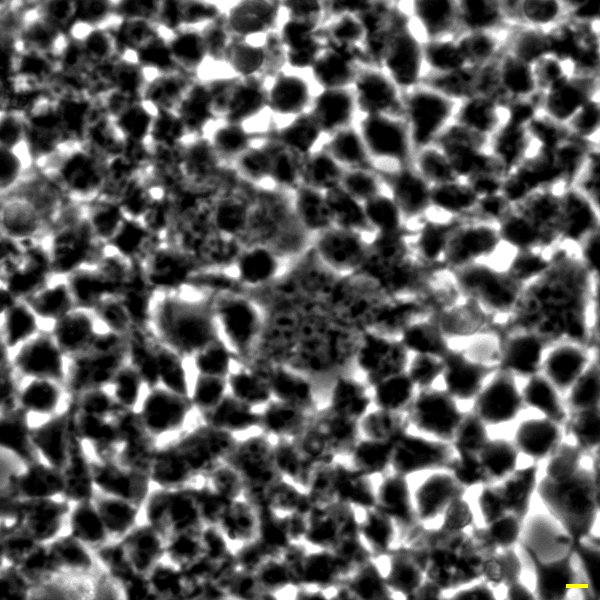

Supplement: Figure 3—figure supplement 1—source data 1. [file elife-97017-fig3-figsupp1-data1.zip › Figure 3-figure supplement 1_source data 1/V108D.jpg]

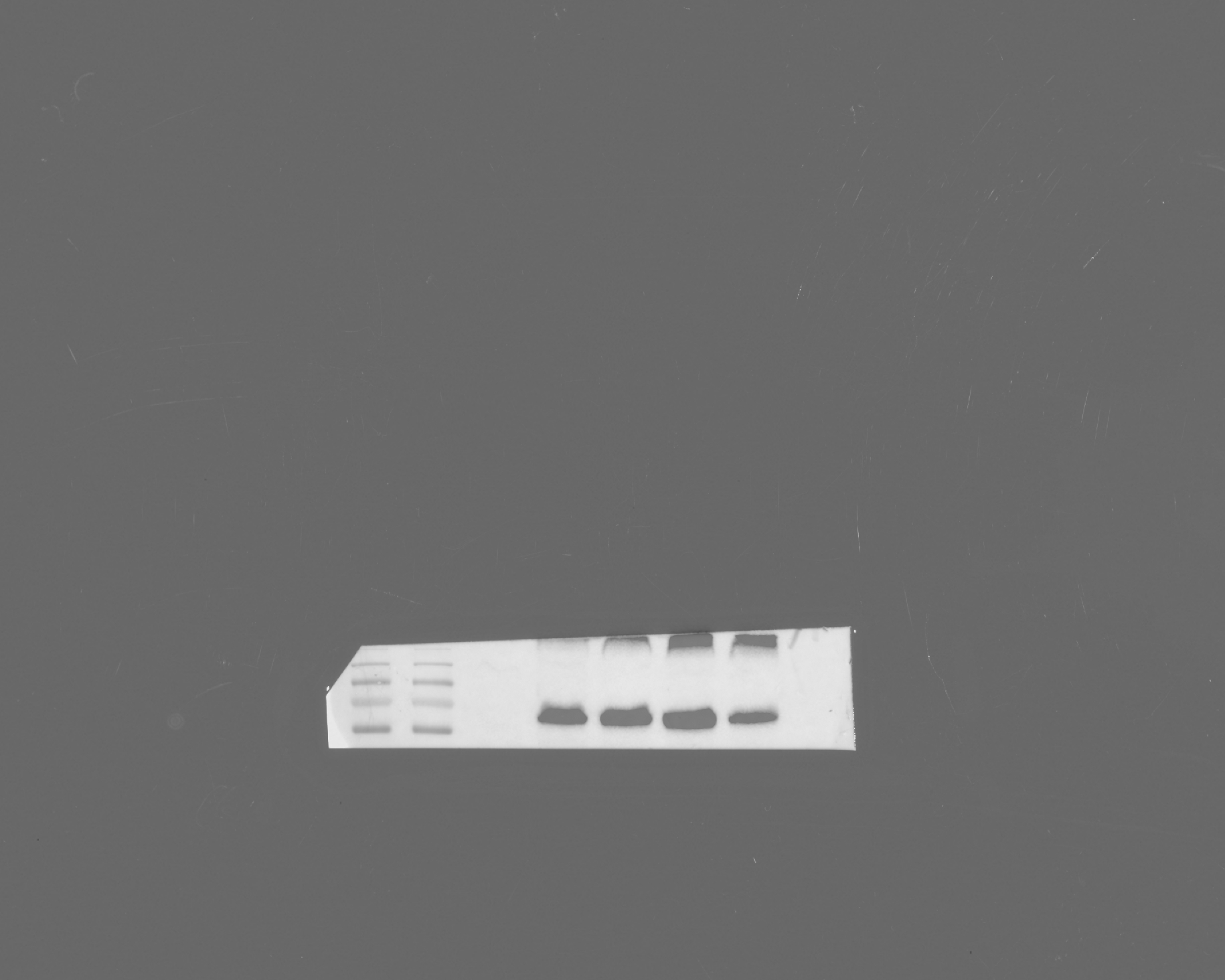

Supplement: Figure 3—figure supplement 1—source data 4. — Original files for western blot analysis. [file elife-97017-fig3-figsupp1-data4.zip › Figure 3-figure supplement 1_source data 4/F0.jpg]

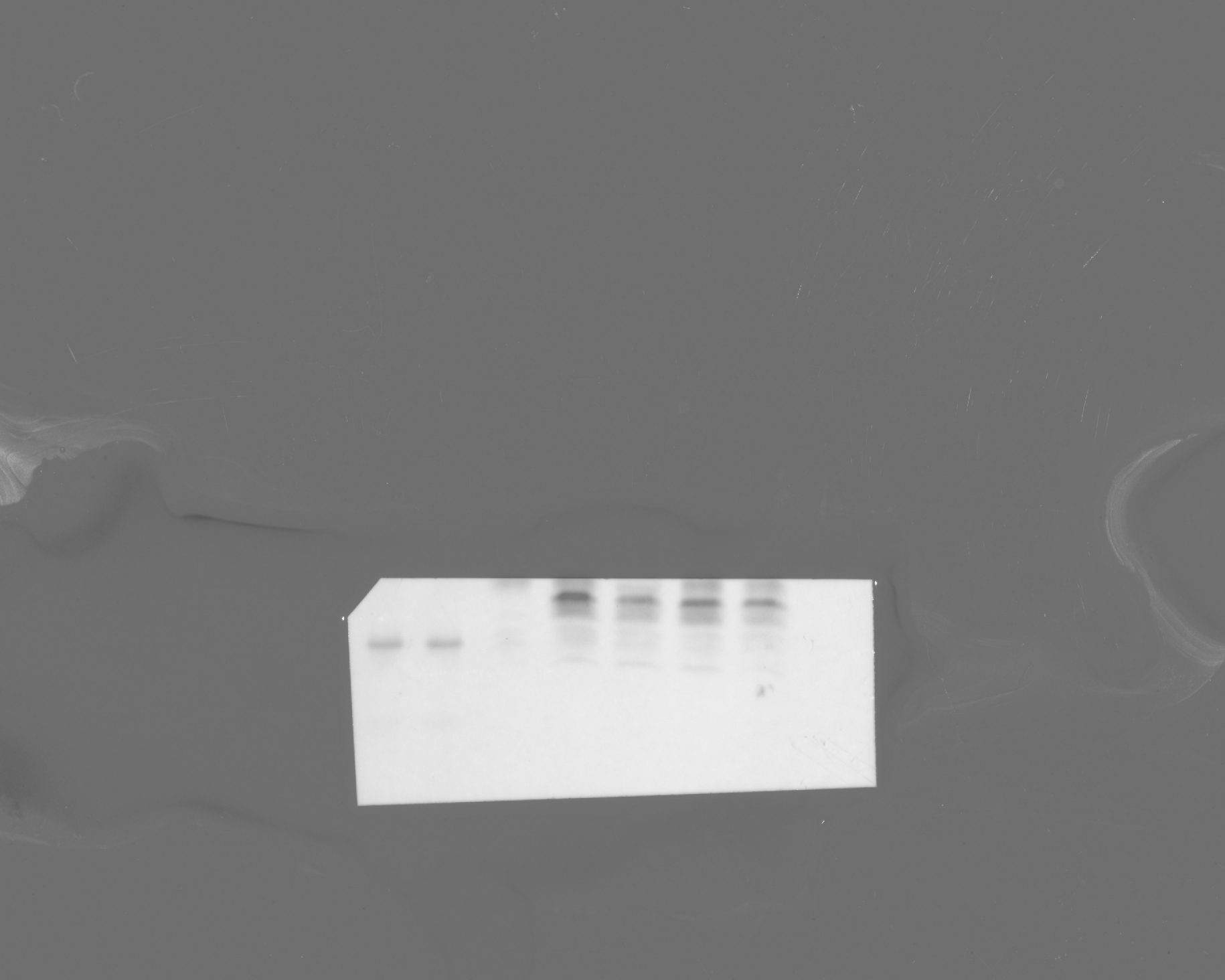

Supplement: Figure 3—figure supplement 1—source data 4. — Original files for western blot analysis. [file elife-97017-fig3-figsupp1-data4.zip › Figure 3-figure supplement 1_source data 4/F2.jpg]

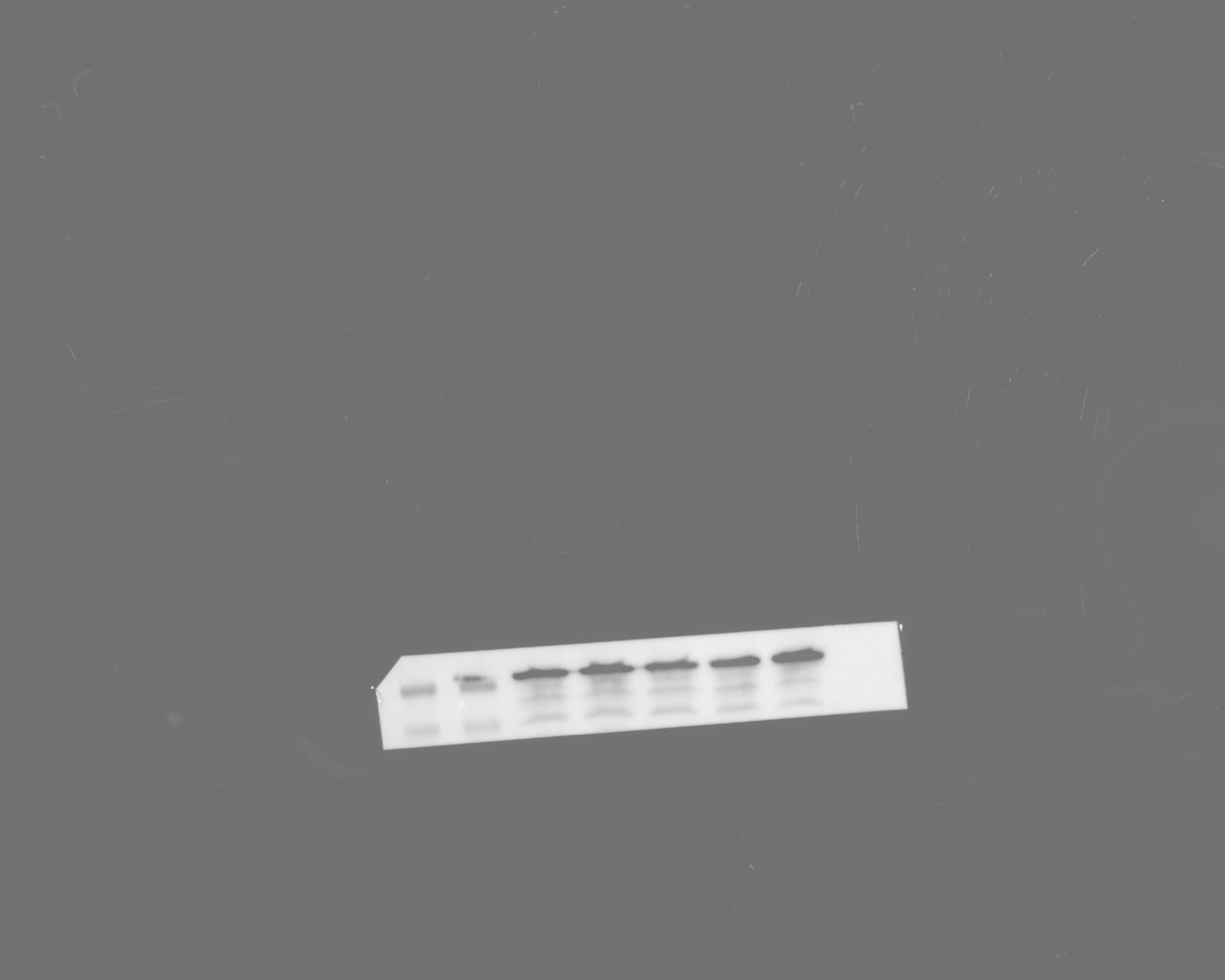

Supplement: Figure 3—figure supplement 1—source data 4. — Original files for western blot analysis. [file elife-97017-fig3-figsupp1-data4.zip › Figure 3-figure supplement 1_source data 4/GAPDH.jpg]

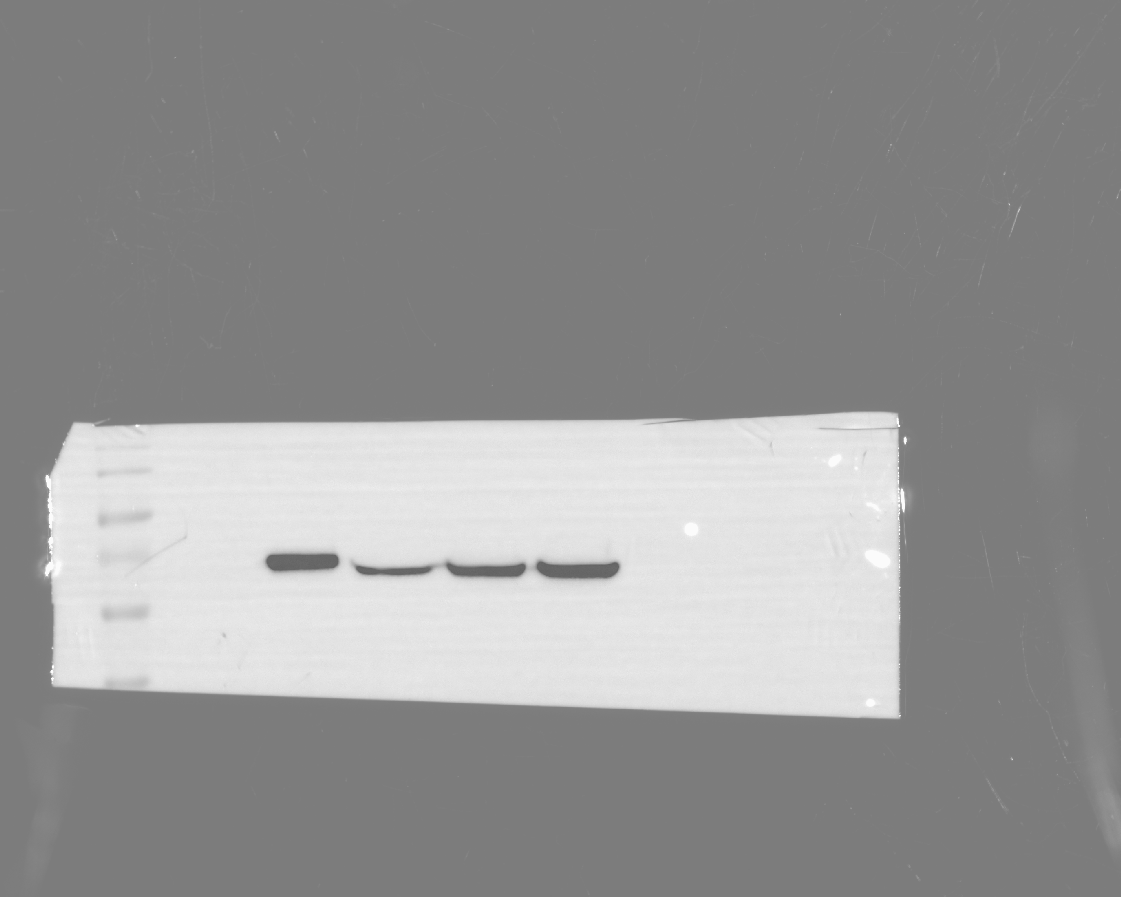

Supplement: Figure 3—figure supplement 1—source data 7. — Original files for western blot analysis. [file elife-97017-fig3-figsupp1-data7.zip › Figure 3-figure supplement 1_source data 7/F0.tif]

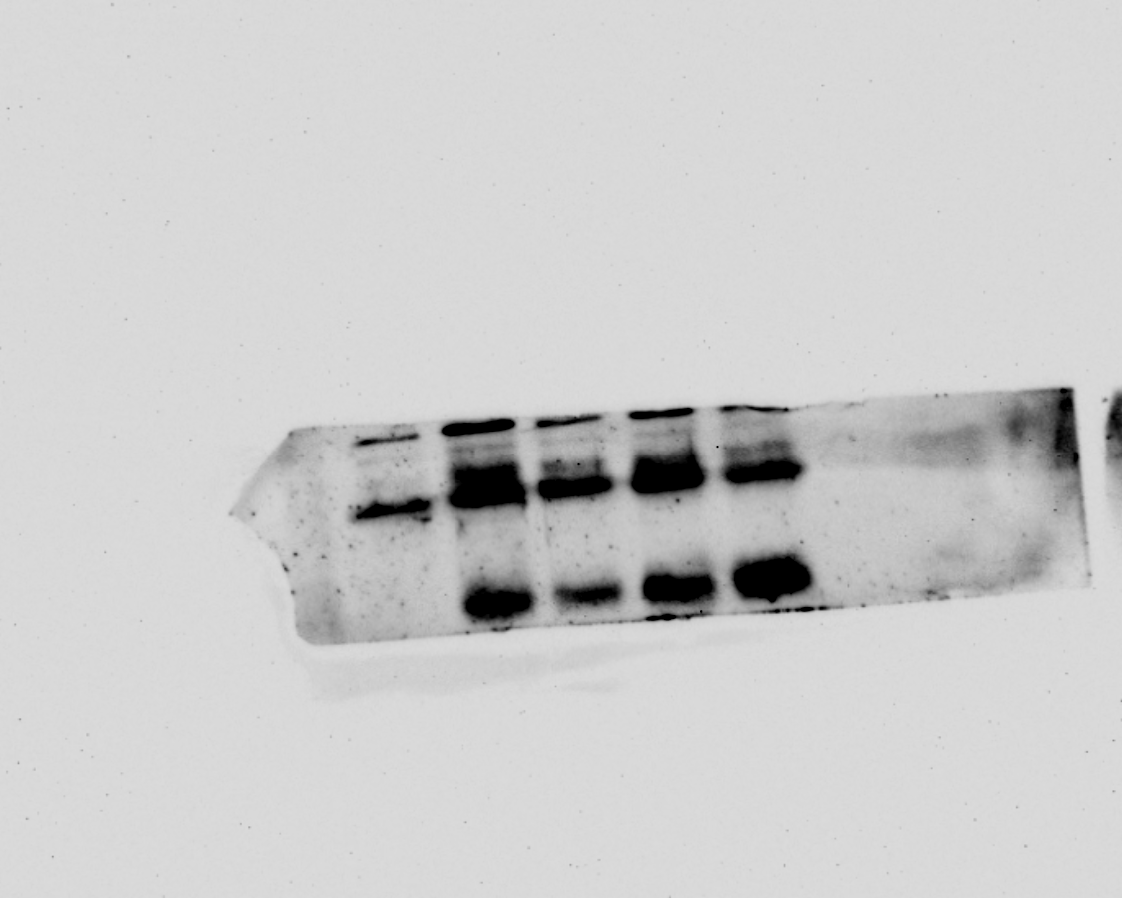

Supplement: Figure 3—figure supplement 1—source data 7. — Original files for western blot analysis. [file elife-97017-fig3-figsupp1-data7.zip › Figure 3-figure supplement 1_source data 7/F2.tif]

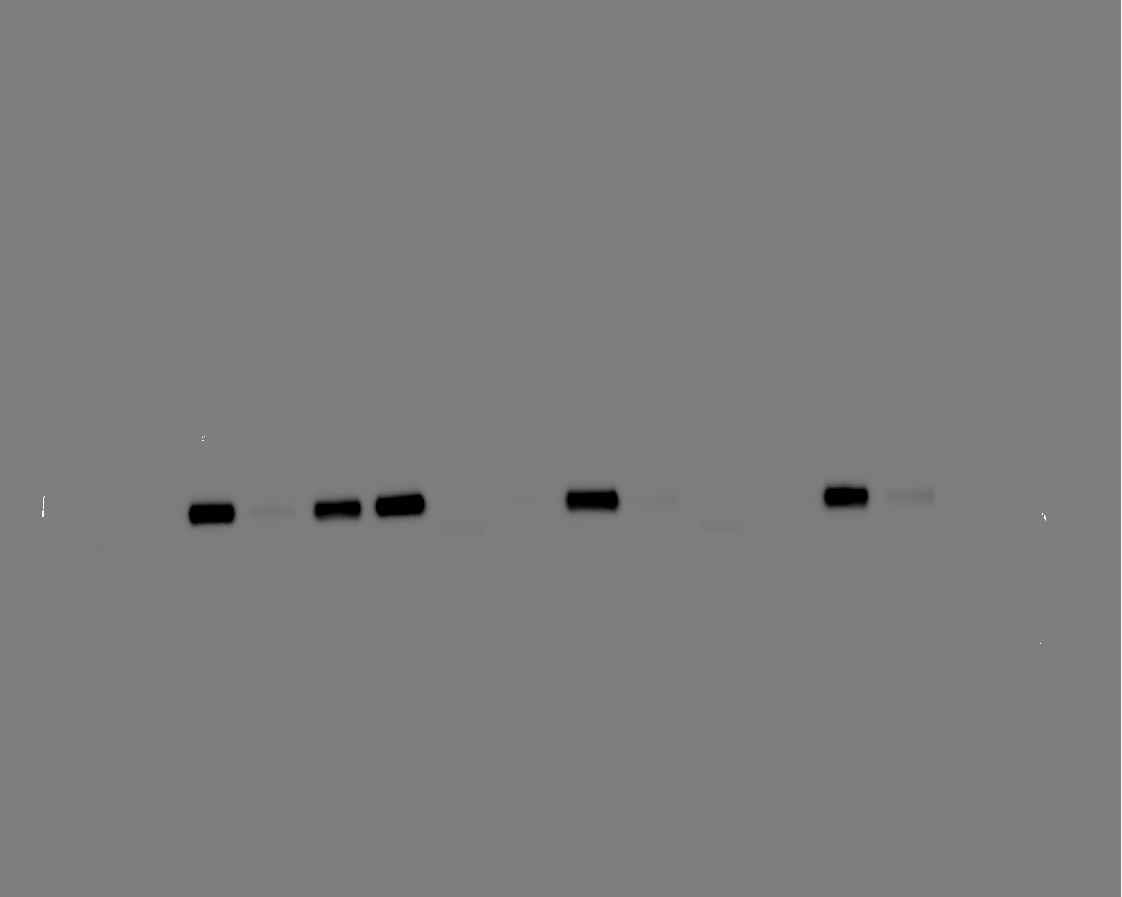

Supplement: Figure 3—figure supplement 1—source data 7. — Original files for western blot analysis. [file elife-97017-fig3-figsupp1-data7.zip › Figure 3-figure supplement 1_source data 7/G.tif]

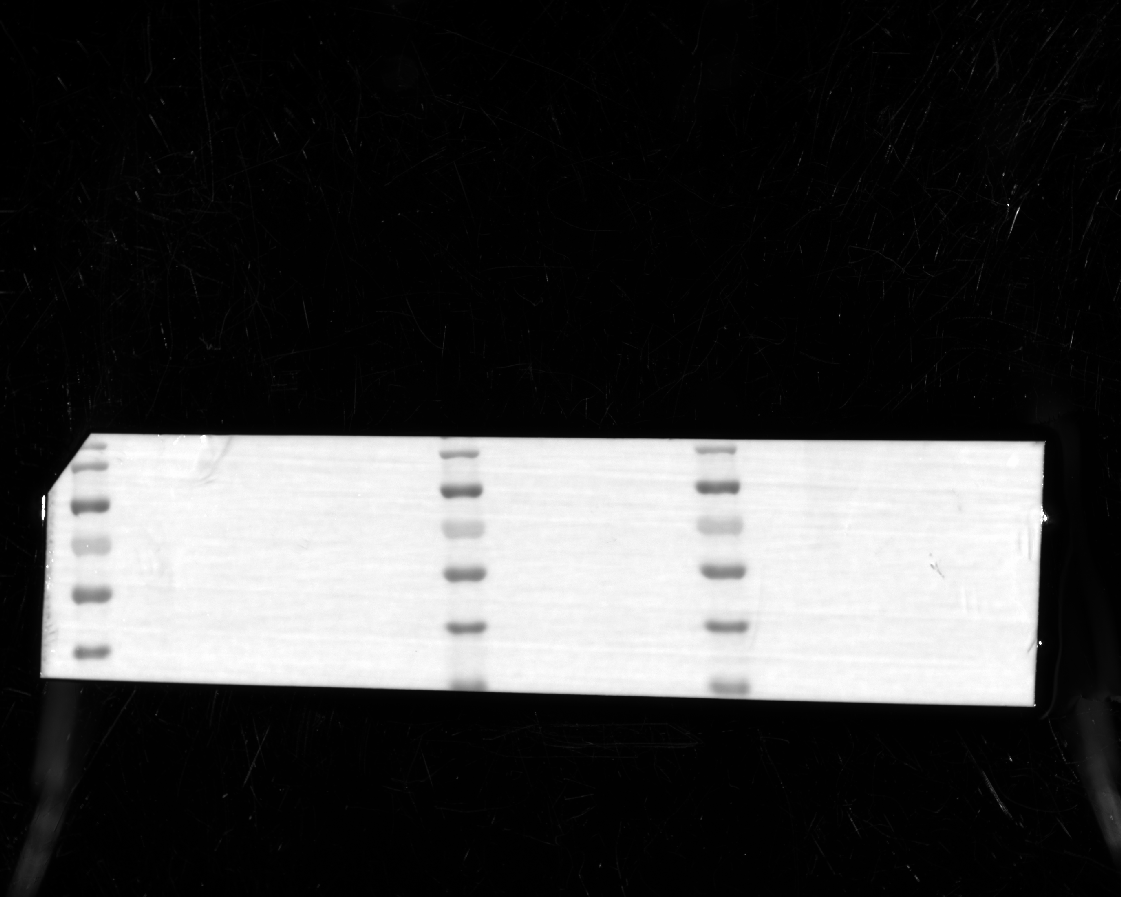

Supplement: Figure 3—figure supplement 1—source data 7. — Original files for western blot analysis. [file elife-97017-fig3-figsupp1-data7.zip › Figure 3-figure supplement 1_source data 7/G-ladder.tif]

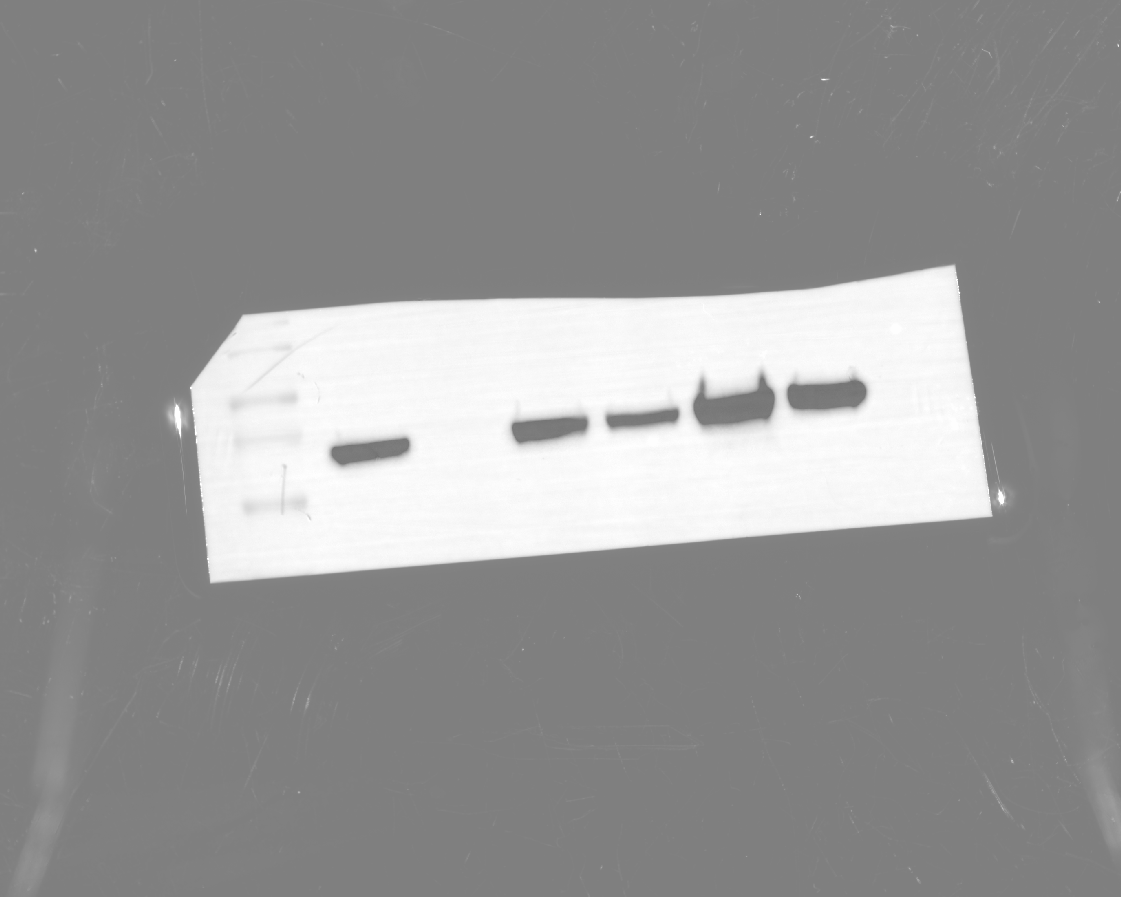

Supplement: Figure 3—figure supplement 1—source data 10. — Original files for western blot analysis. [file elife-97017-fig3-figsupp1-data10.zip › Figure 3-figure supplement 1_source data 10/F0.tif]

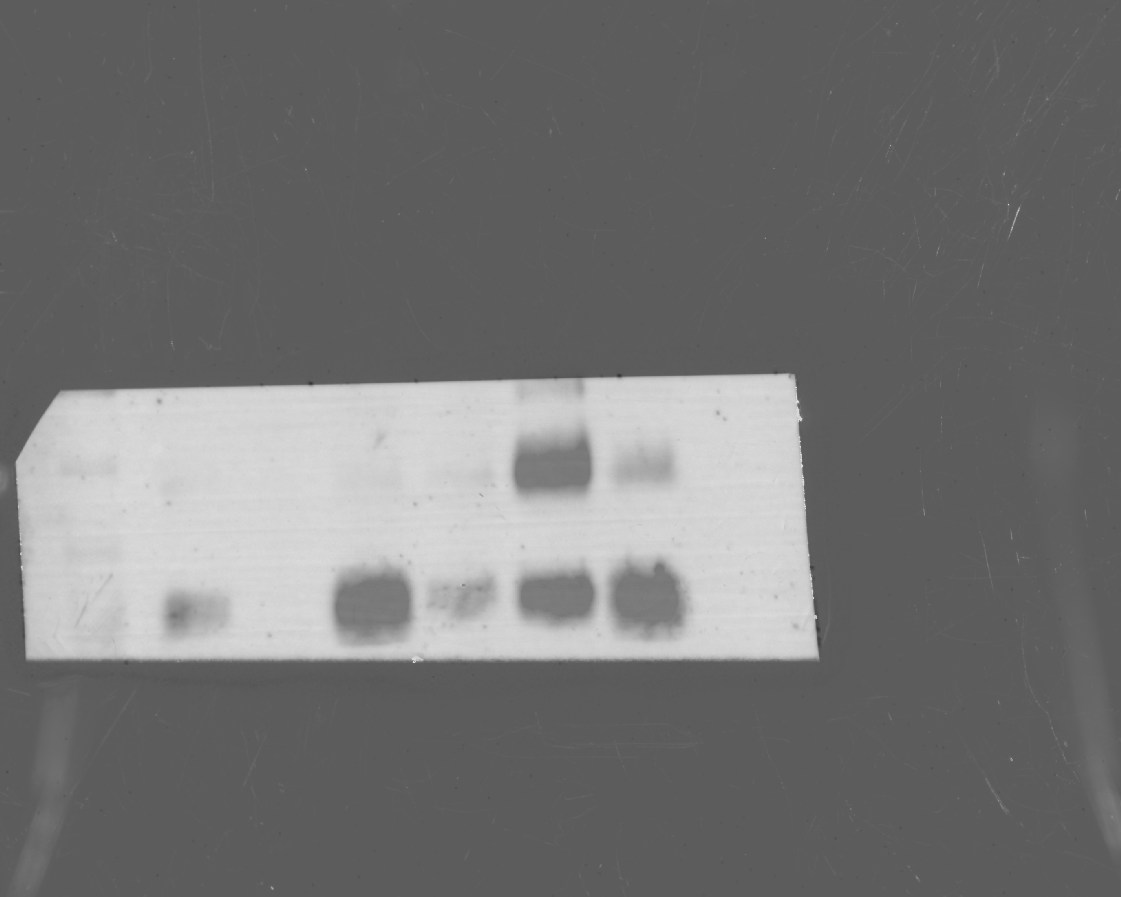

Supplement: Figure 3—figure supplement 1—source data 10. — Original files for western blot analysis. [file elife-97017-fig3-figsupp1-data10.zip › Figure 3-figure supplement 1_source data 10/F2.tif]

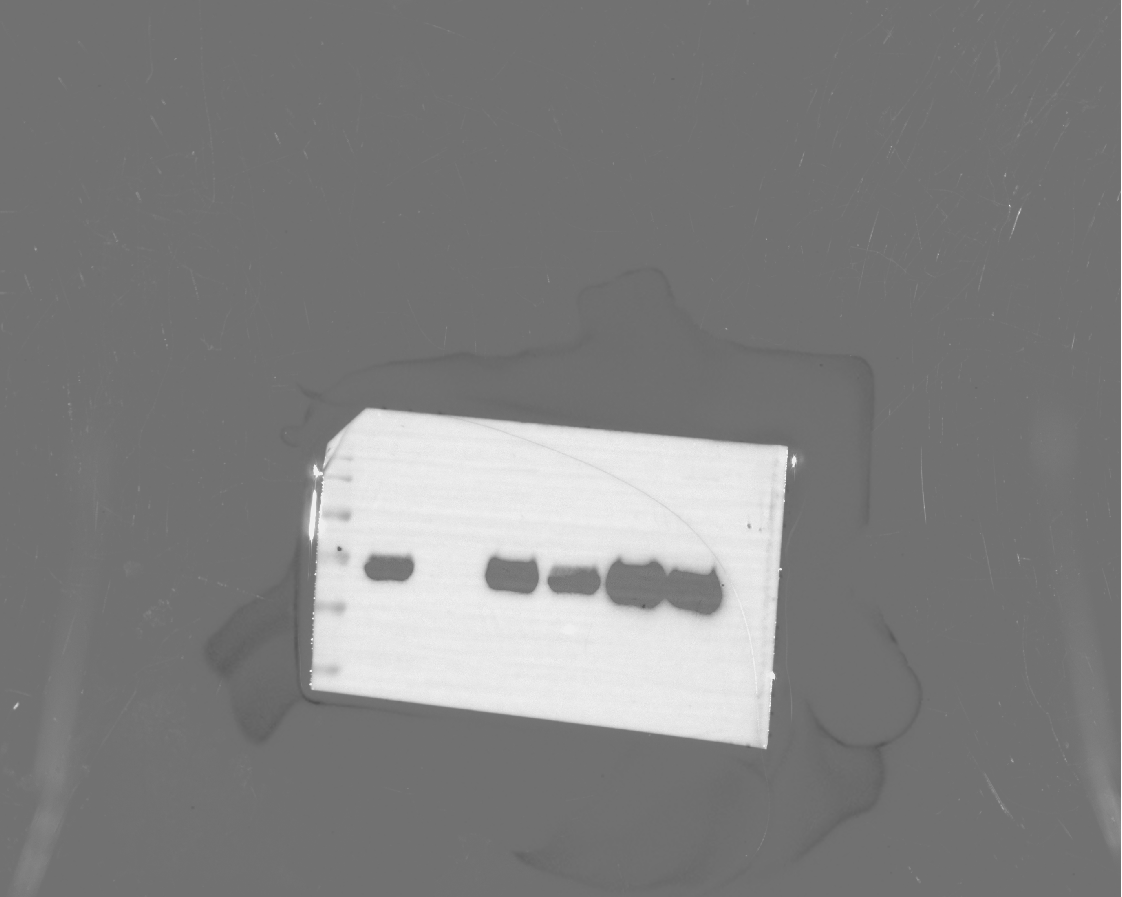

Supplement: Figure 3—figure supplement 1—source data 10. — Original files for western blot analysis. [file elife-97017-fig3-figsupp1-data10.zip › Figure 3-figure supplement 1_source data 10/M.tif]

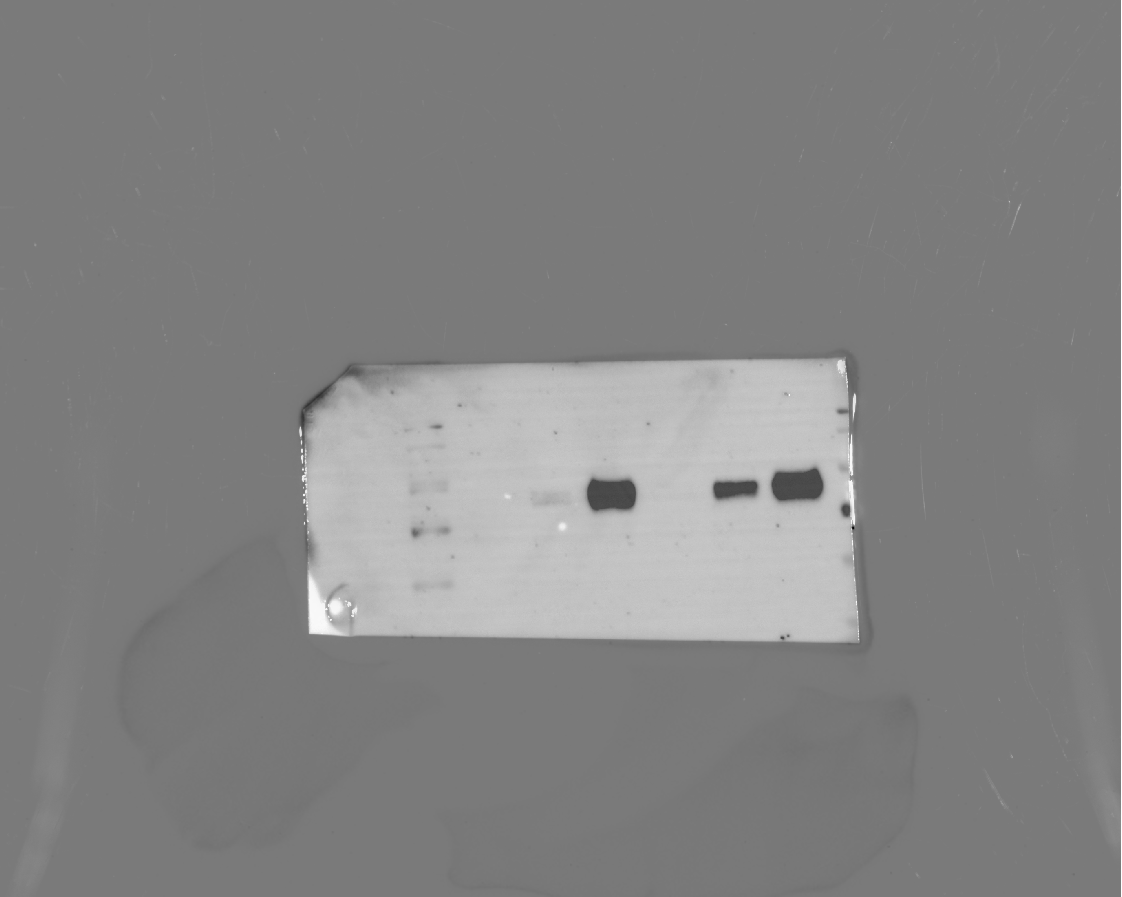

Supplement: Figure 3—figure supplement 1—source data 10. — Original files for western blot analysis. [file elife-97017-fig3-figsupp1-data10.zip › Figure 3-figure supplement 1_source data 10/G.tif]

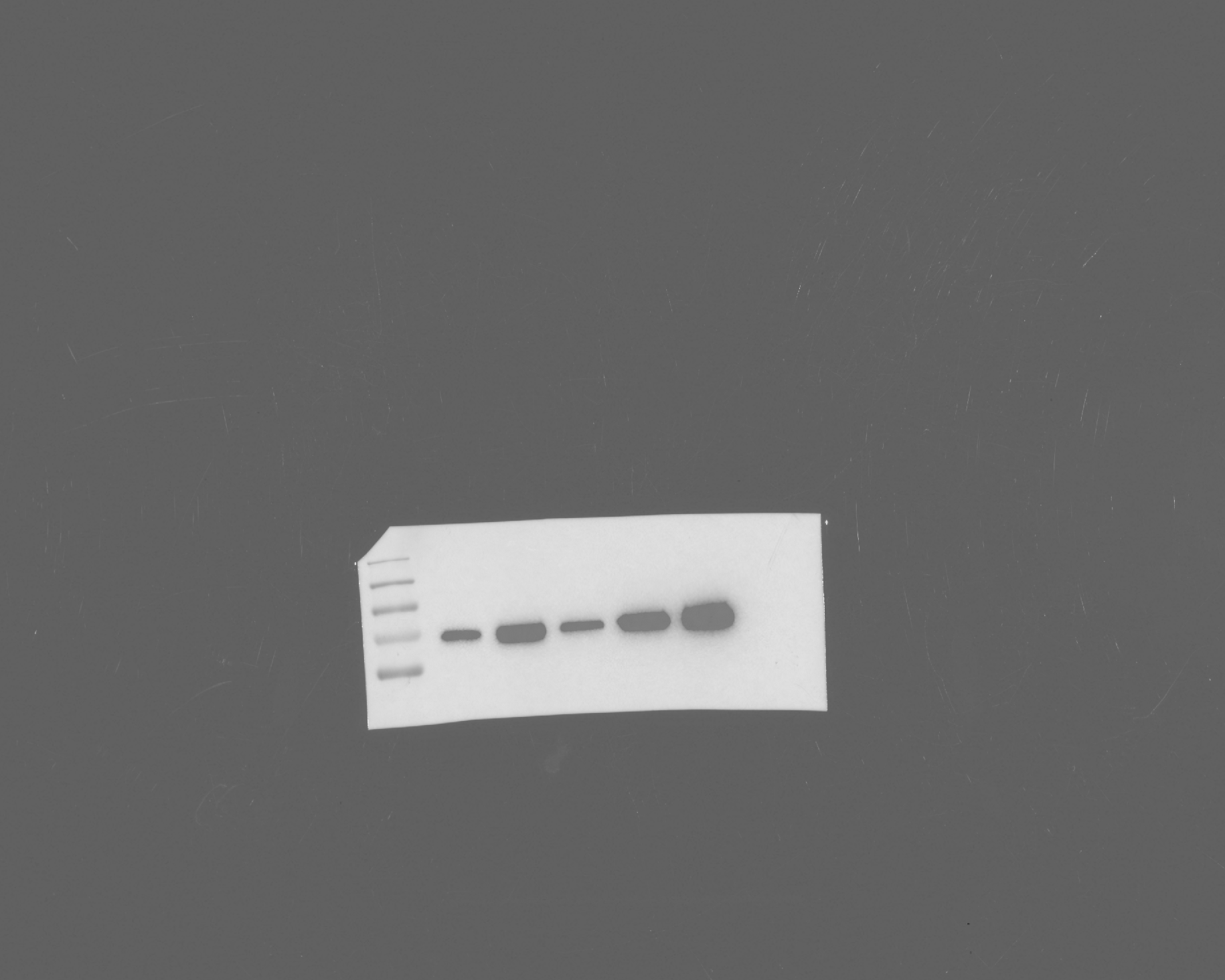

Supplement: Figure 4—source data 1. — Original files for western blot analysis. [file elife-97017-fig4-data1.zip › Figure 4_source data 1/F0.tif]

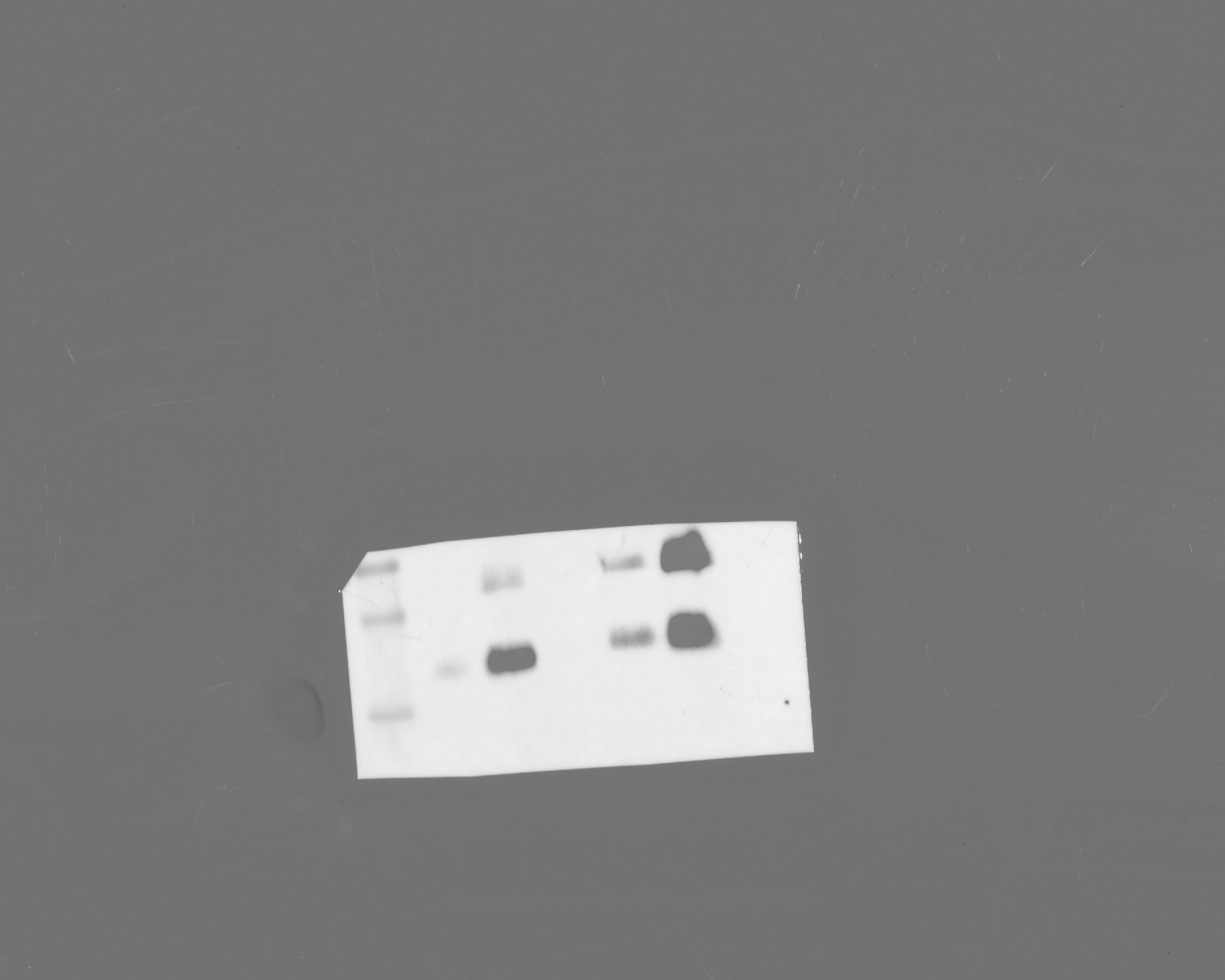

Supplement: Figure 4—source data 1. — Original files for western blot analysis. [file elife-97017-fig4-data1.zip › Figure 4_source data 1/F2.tif]

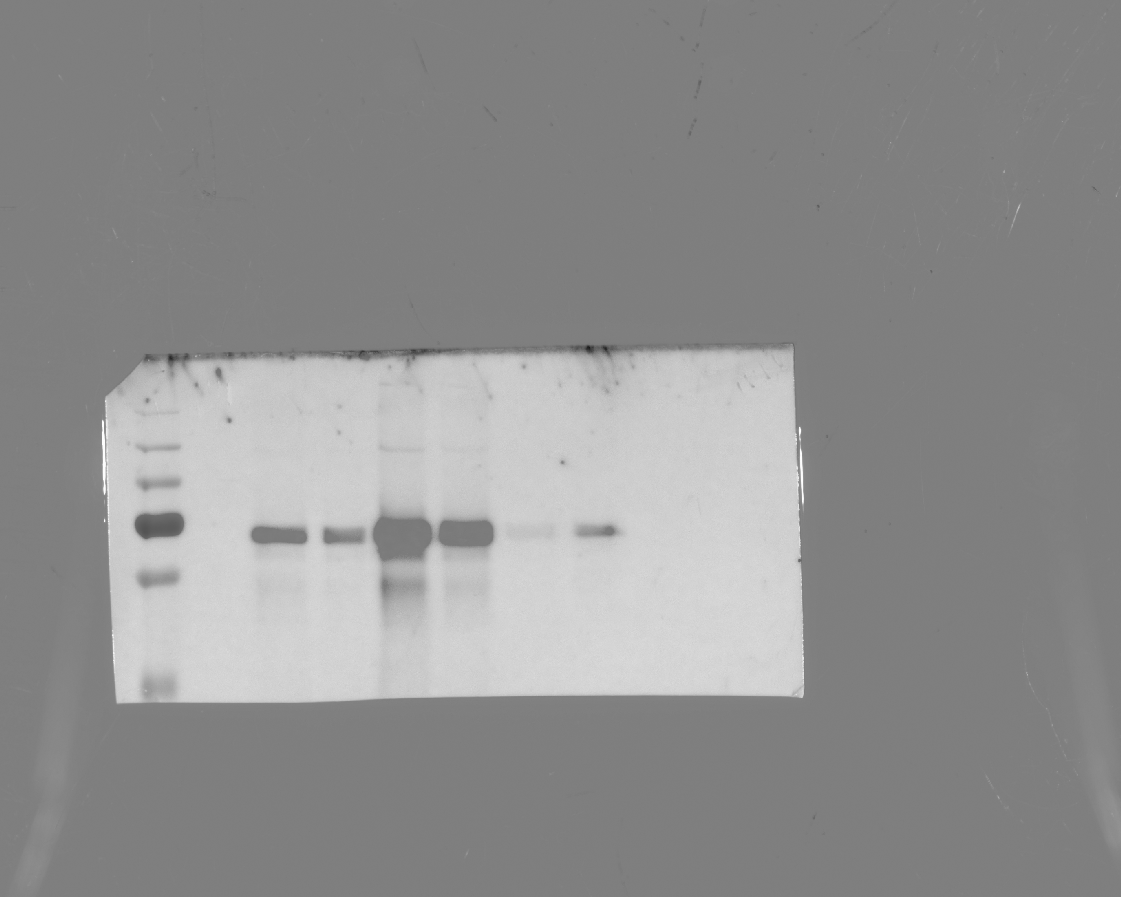

Supplement: Figure 4—source data 1. — Original files for western blot analysis. [file elife-97017-fig4-data1.zip › Figure 4_source data 1/M.tif]

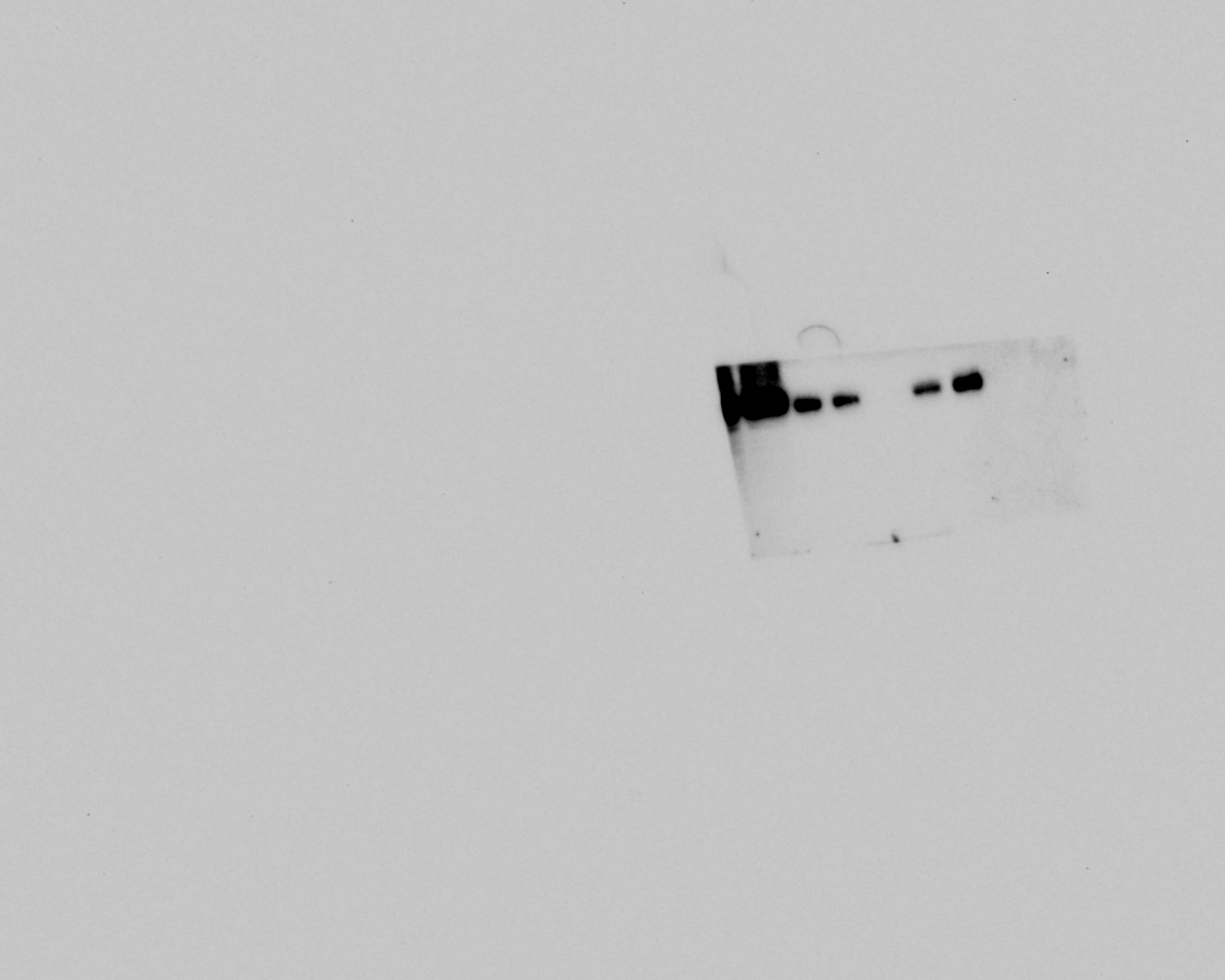

Supplement: Figure 4—source data 1. — Original files for western blot analysis. [file elife-97017-fig4-data1.zip › Figure 4_source data 1/G.tif]

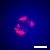

Supplement: Figure 4—source data 3. [file elife-97017-fig4-data3.zip › Figure 4_source data 3/WT(RGB).jpg]

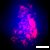

Supplement: Figure 4—source data 3. [file elife-97017-fig4-data3.zip › Figure 4_source data 3/L53D(RGB).jpg]

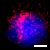

Supplement: Figure 4—source data 3. [file elife-97017-fig4-data3.zip › Figure 4_source data 3/V108D(RGB).jpg]

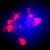

Supplement: Figure 4—source data 3. [file elife-97017-fig4-data3.zip › Figure 4_source data 3/Q393L(RGB).jpg]

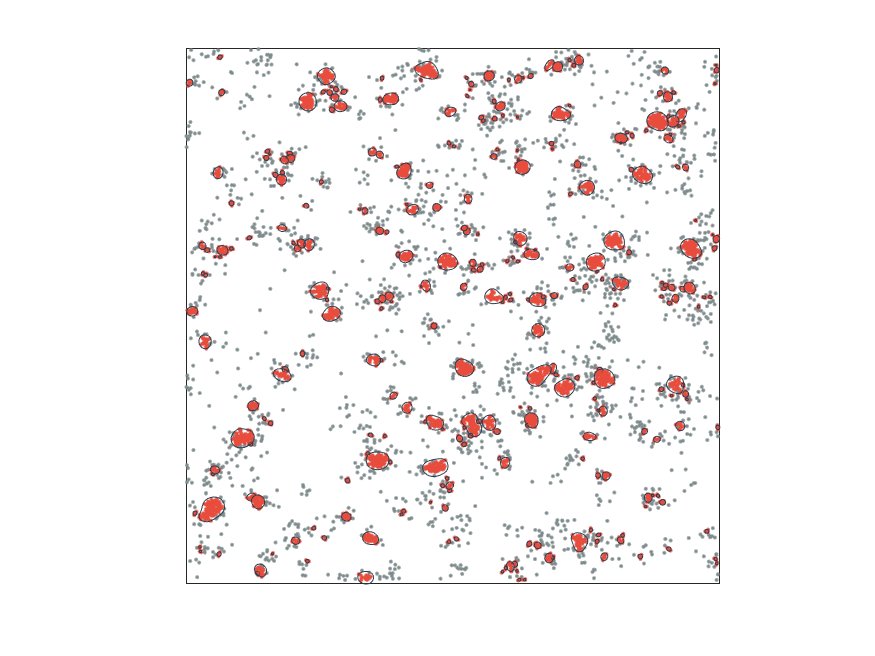

Supplement: Figure 5—source data 1. [file elife-97017-fig5-data1.zip › Figure 5_source data 1/LI4A-ClusterMap.jpg]

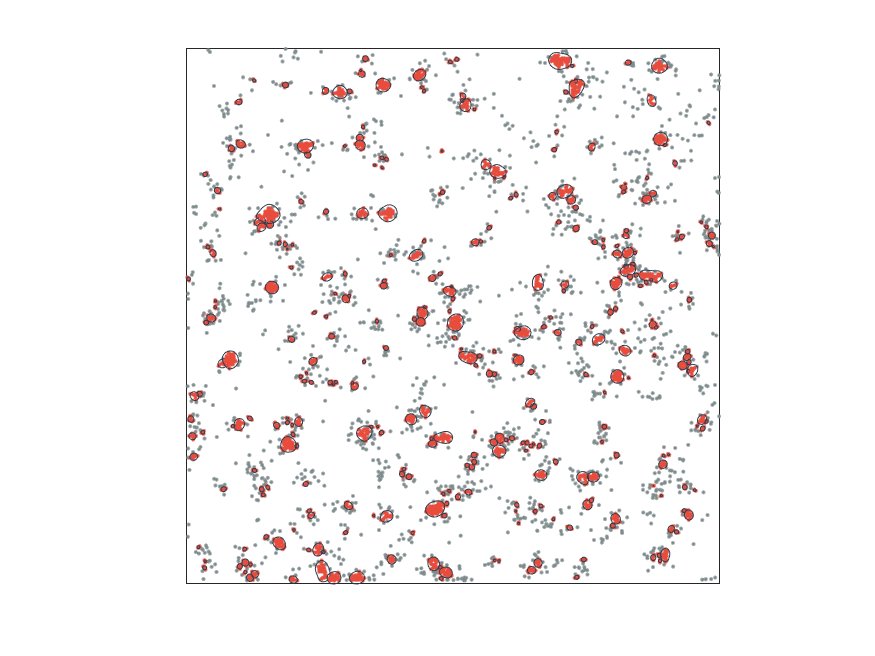

Supplement: Figure 5—source data 1. [file elife-97017-fig5-data1.zip › Figure 5_source data 1/WT-ClusterMap.jpg]

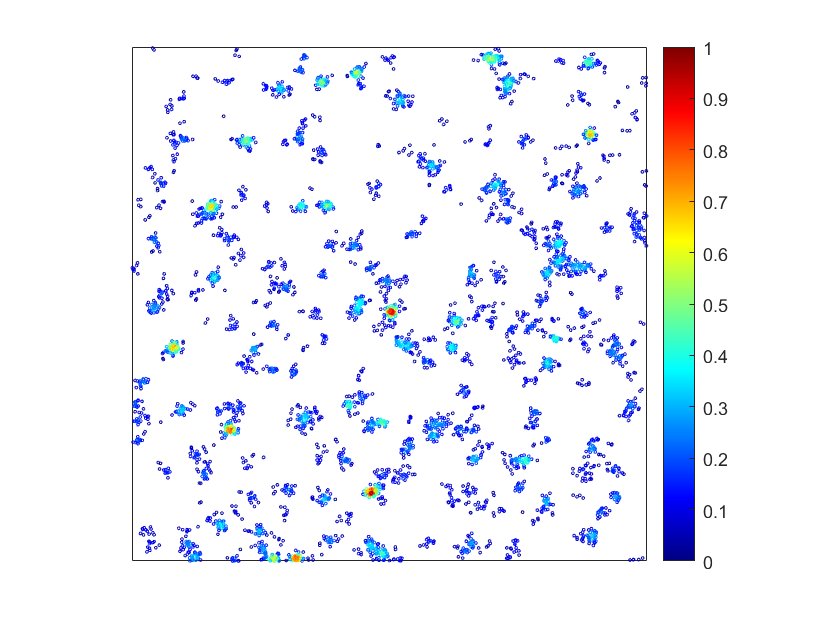

Supplement: Figure 5—source data 1. [file elife-97017-fig5-data1.zip › Figure 5_source data 1/WT-DensityMap.jpg]

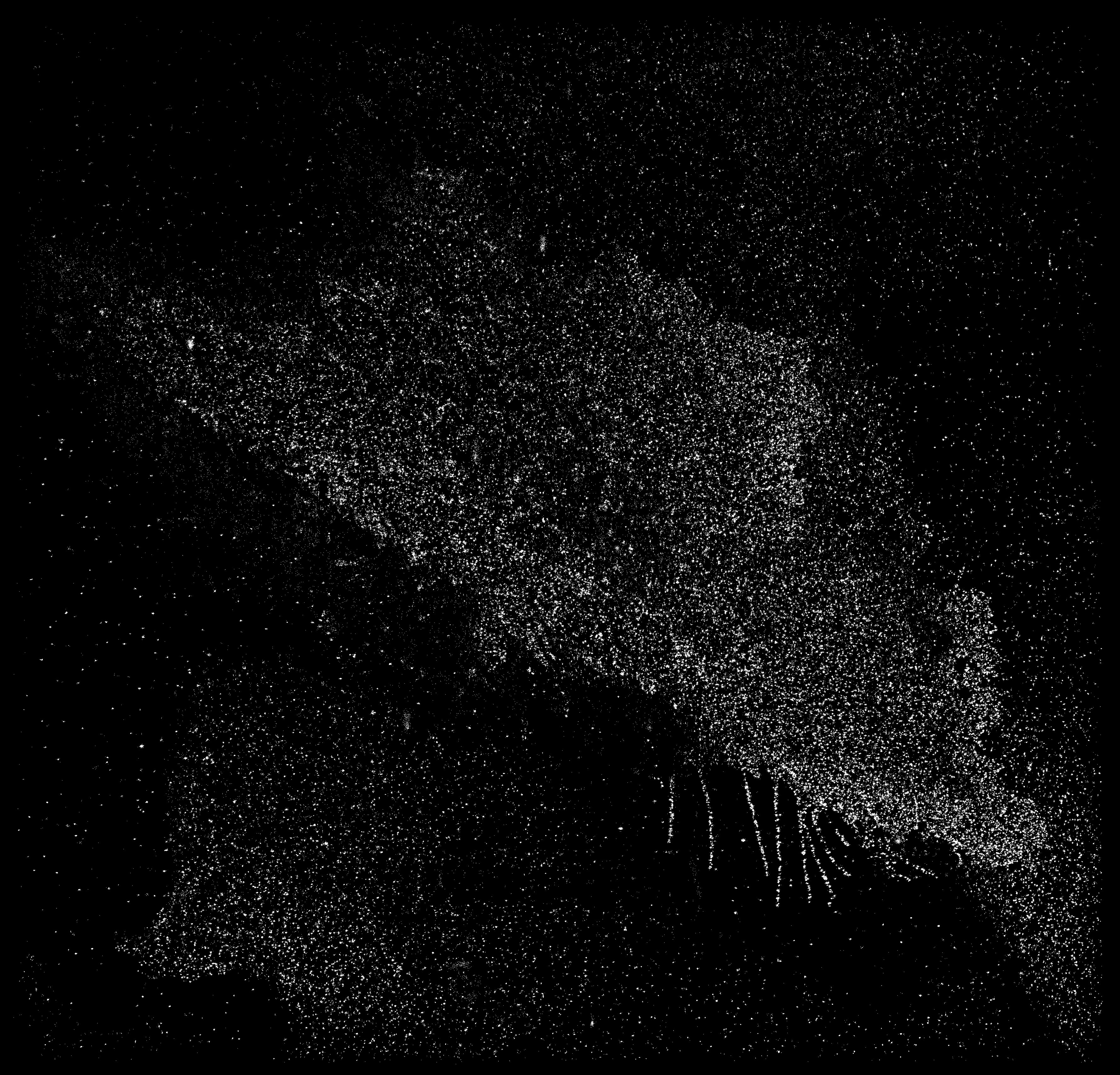

Supplement: Figure 5—source data 1. [file elife-97017-fig5-data1.zip › Figure 5_source data 1/WT.jpg]

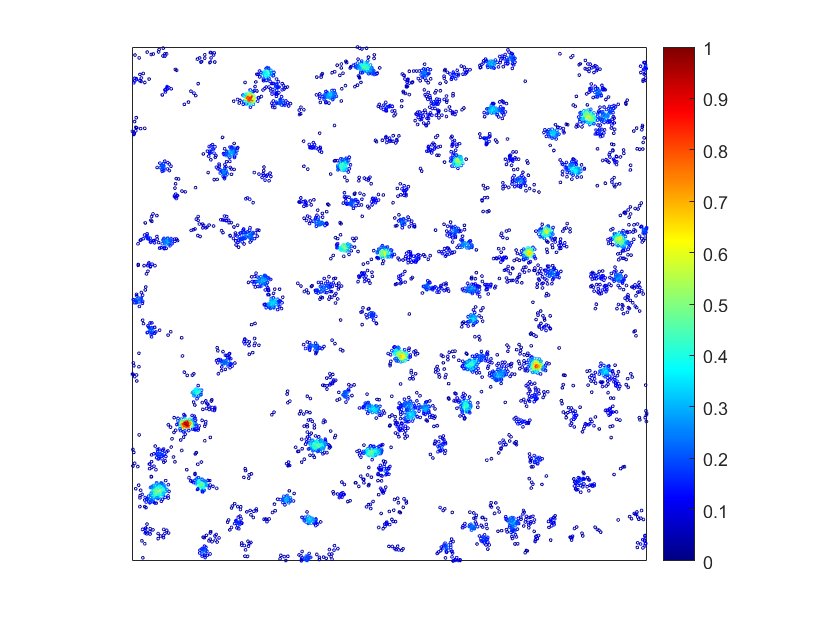

Supplement: Figure 5—source data 1. [file elife-97017-fig5-data1.zip › Figure 5_source data 1/LI4A-DenstityMap.jpg]

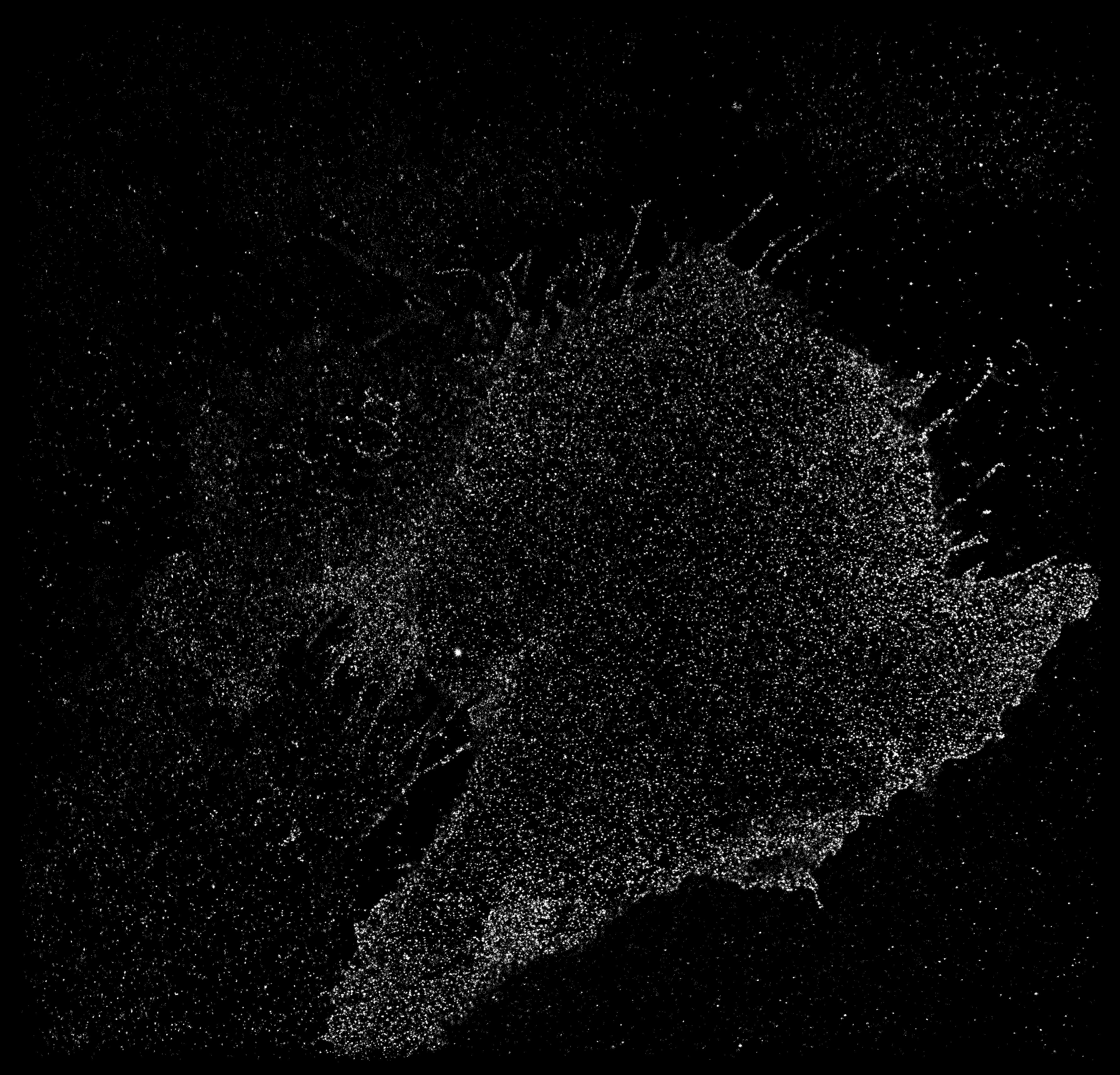

Supplement: Figure 5—source data 1. [file elife-97017-fig5-data1.zip › Figure 5_source data 1/LI4A.jpg]

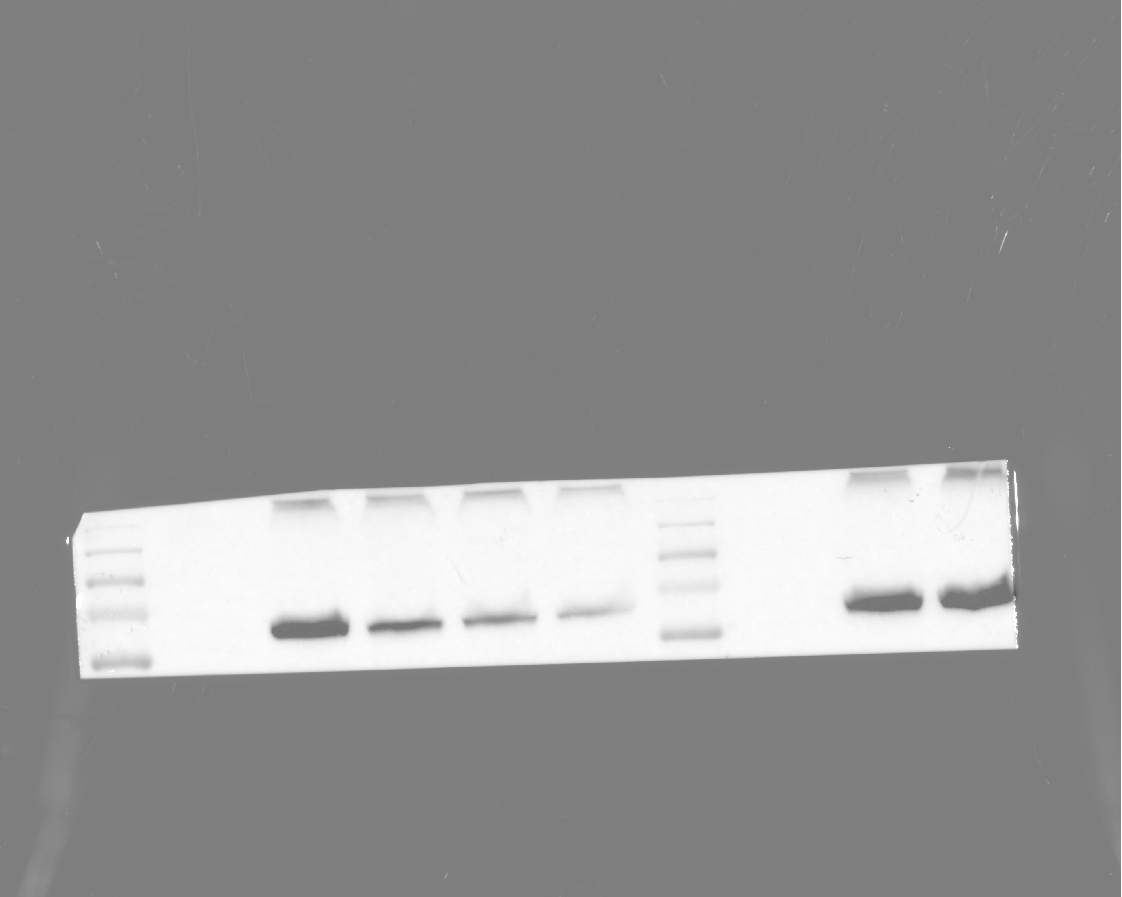

Supplement: Figure 5—figure supplement 1—source data 1. — Original files for western blot analysis. [file elife-97017-fig5-figsupp1-data1.zip › Figure 5-figure supplement 1_source data 1/F0.jpg]

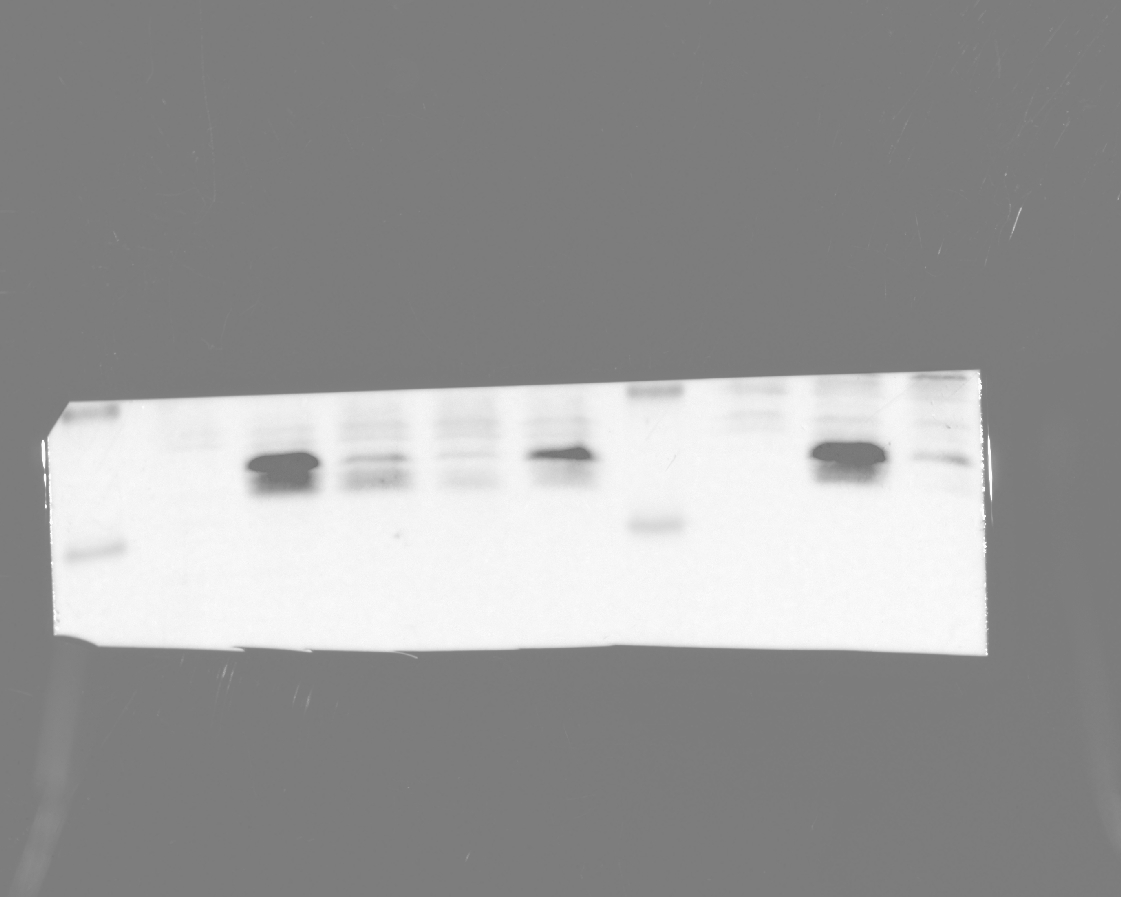

Supplement: Figure 5—figure supplement 1—source data 1. — Original files for western blot analysis. [file elife-97017-fig5-figsupp1-data1.zip › Figure 5-figure supplement 1_source data 1/F2.jpg]

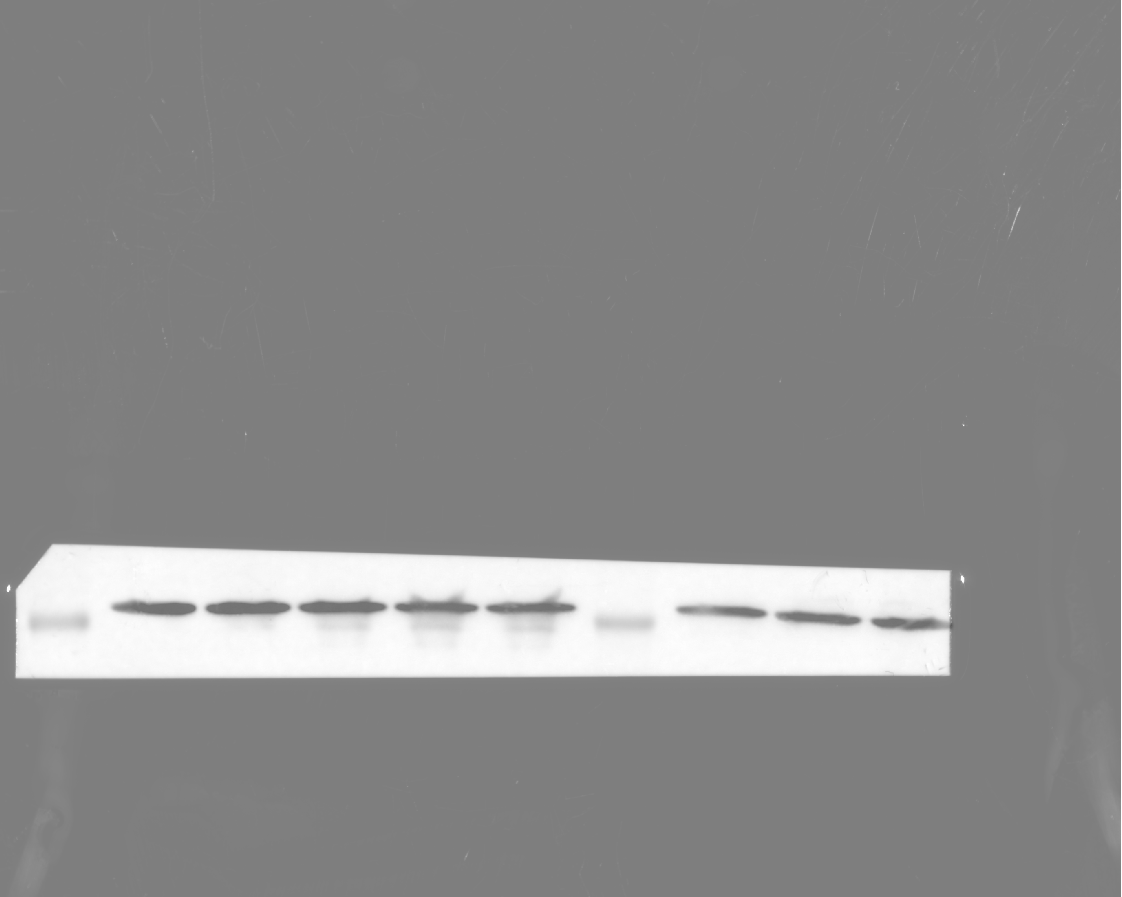

Supplement: Figure 5—figure supplement 1—source data 1. — Original files for western blot analysis. [file elife-97017-fig5-figsupp1-data1.zip › Figure 5-figure supplement 1_source data 1/GAPDH.tif]

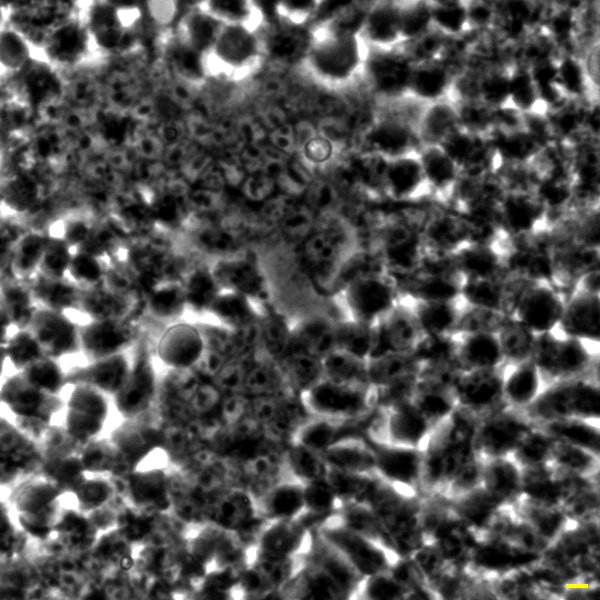

Supplement: Figure 5—figure supplement 1—source data 3. [file elife-97017-fig5-figsupp1-data3.zip › Figure 5-figure supplement 1_source data 3/WT.jpg]

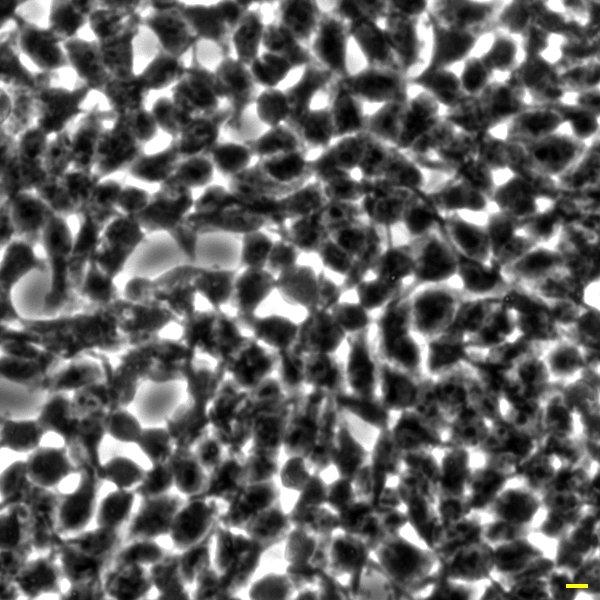

Supplement: Figure 5—figure supplement 1—source data 3. [file elife-97017-fig5-figsupp1-data3.zip › Figure 5-figure supplement 1_source data 3/NC.jpg]

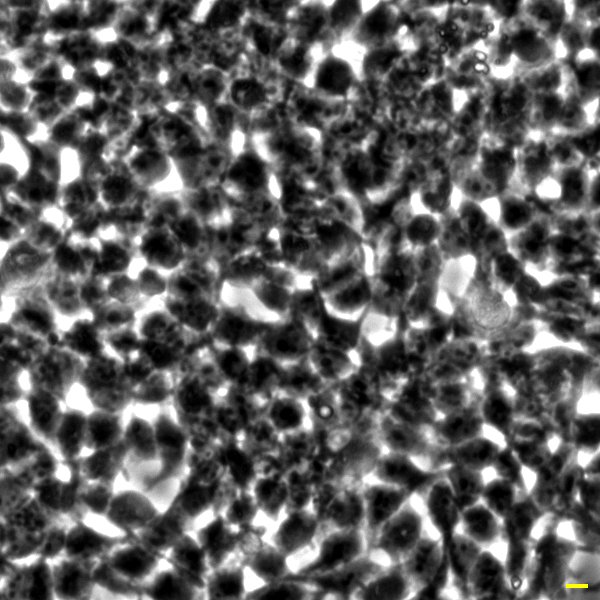

Supplement: Figure 5—figure supplement 1—source data 3. [file elife-97017-fig5-figsupp1-data3.zip › Figure 5-figure supplement 1_source data 3/LI4A.jpg]

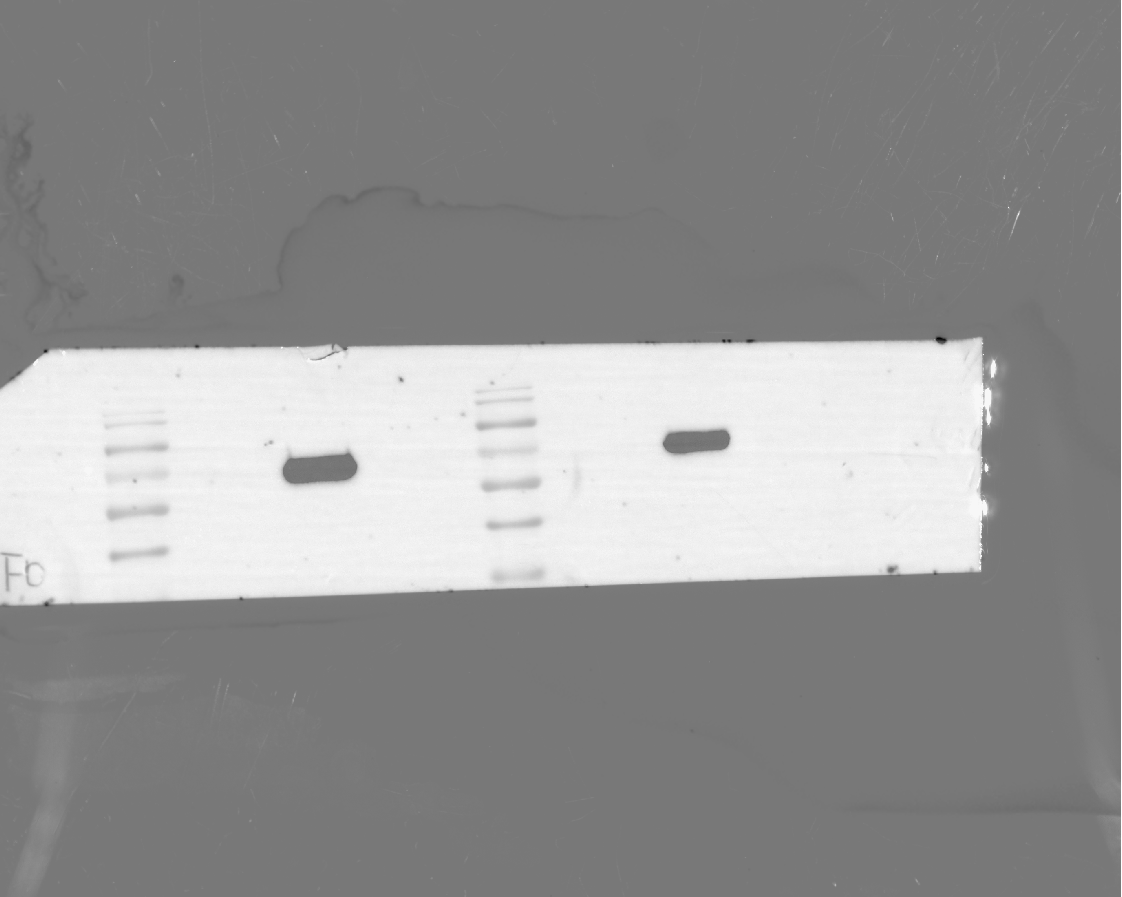

Supplement: Figure 5—figure supplement 1—source data 5. — Original files for western blot analysis. [file elife-97017-fig5-figsupp1-data5.zip › Figure 5-figure supplement 1_source data 5/F0.tif]

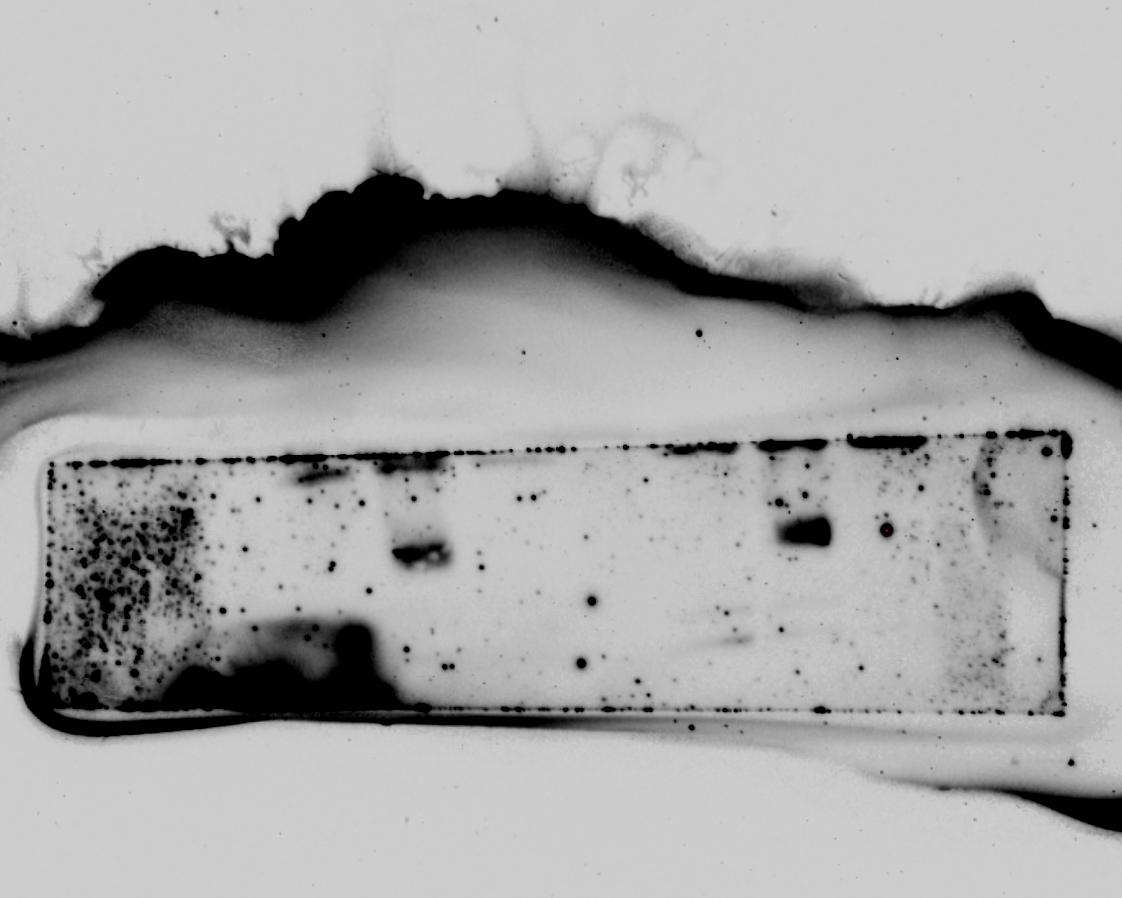

Supplement: Figure 5—figure supplement 1—source data 5. — Original files for western blot analysis. [file elife-97017-fig5-figsupp1-data5.zip › Figure 5-figure supplement 1_source data 5/F2.tif]

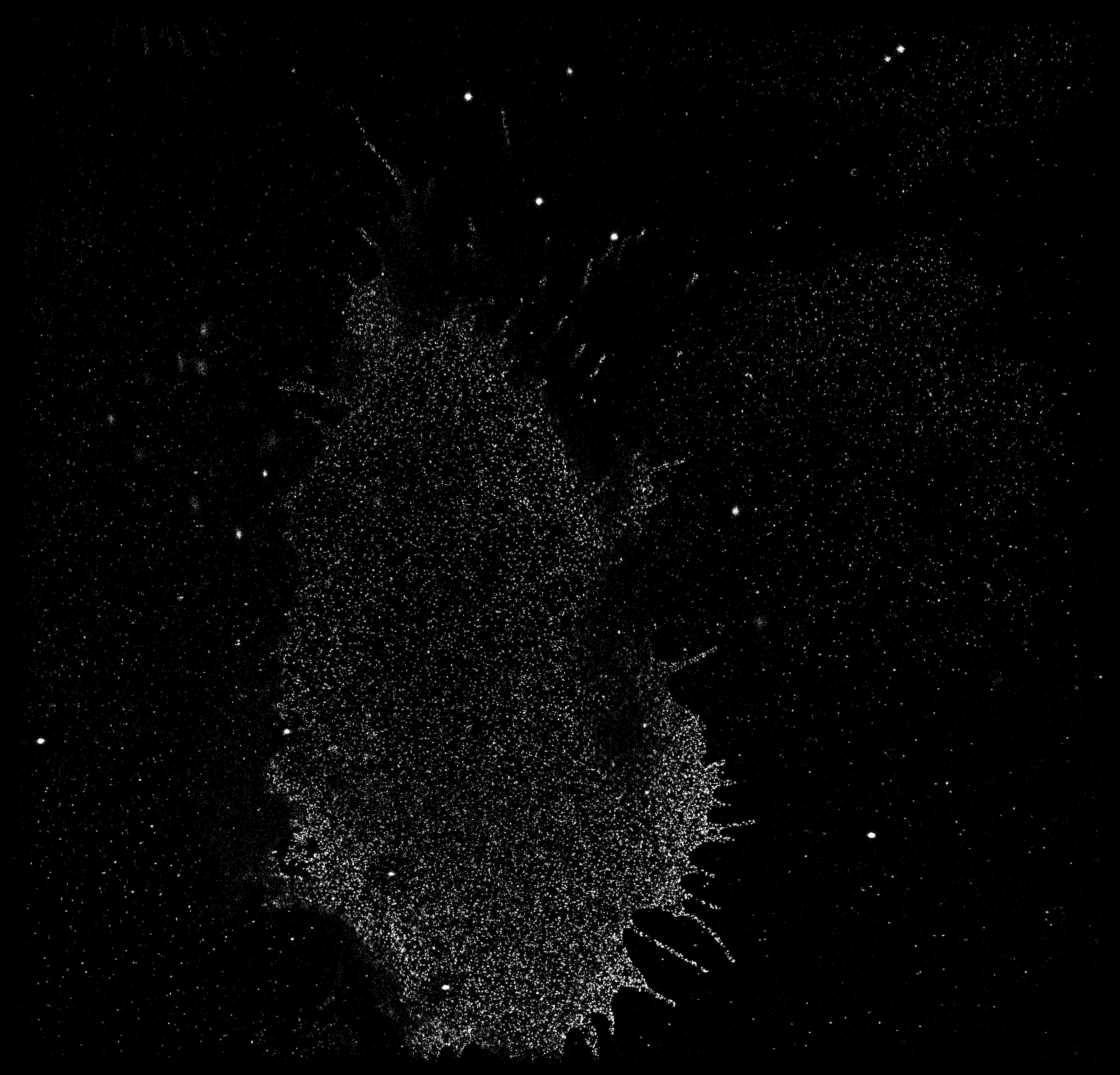

Supplement: Figure 6—source data 1. [file elife-97017-fig6-data1.zip › Figure 6_source data 1/YA.jpg]

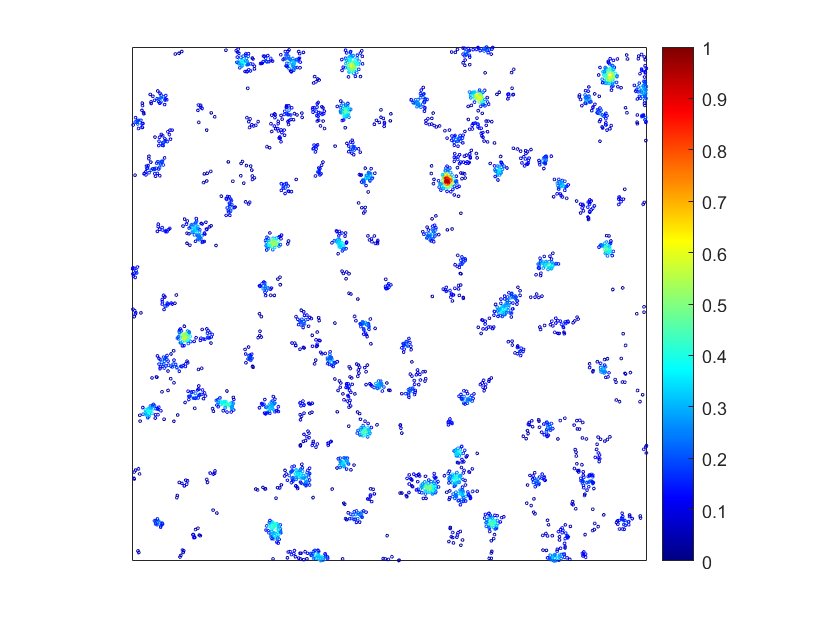

Supplement: Figure 6—source data 1. [file elife-97017-fig6-data1.zip › Figure 6_source data 1/YA-DensityMap.jpg]

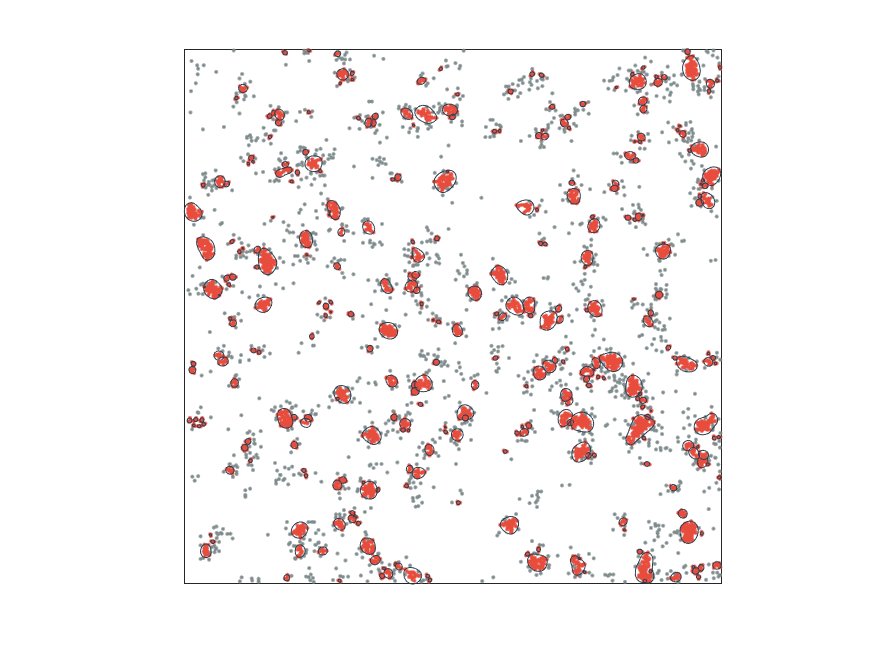

Supplement: Figure 6—source data 1. [file elife-97017-fig6-data1.zip › Figure 6_source data 1/WT-ClusterMap.jpg]

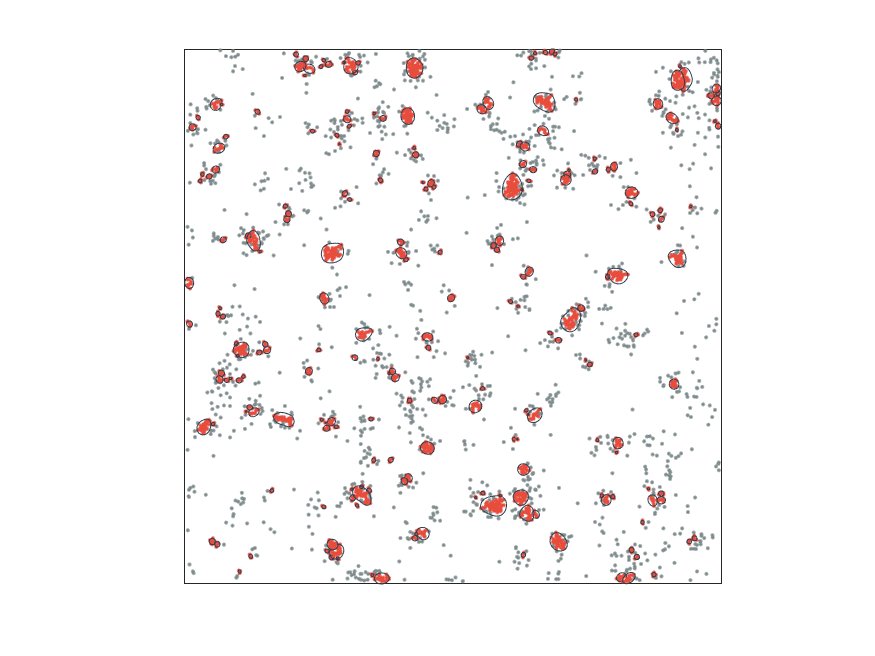

Supplement: Figure 6—source data 1. [file elife-97017-fig6-data1.zip › Figure 6_source data 1/YA-ClusterMap.jpg]

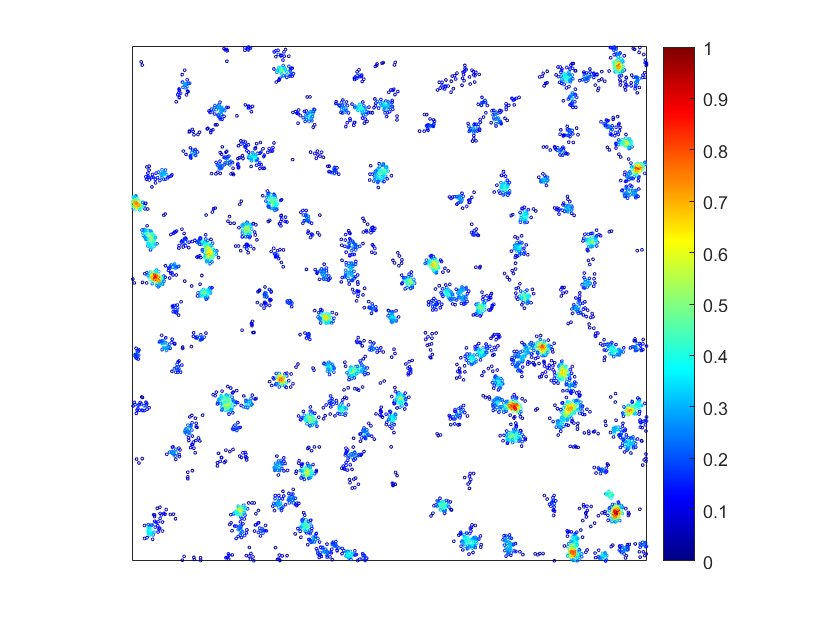

Supplement: Figure 6—source data 1. [file elife-97017-fig6-data1.zip › Figure 6_source data 1/WT-DensityMap.jpg]

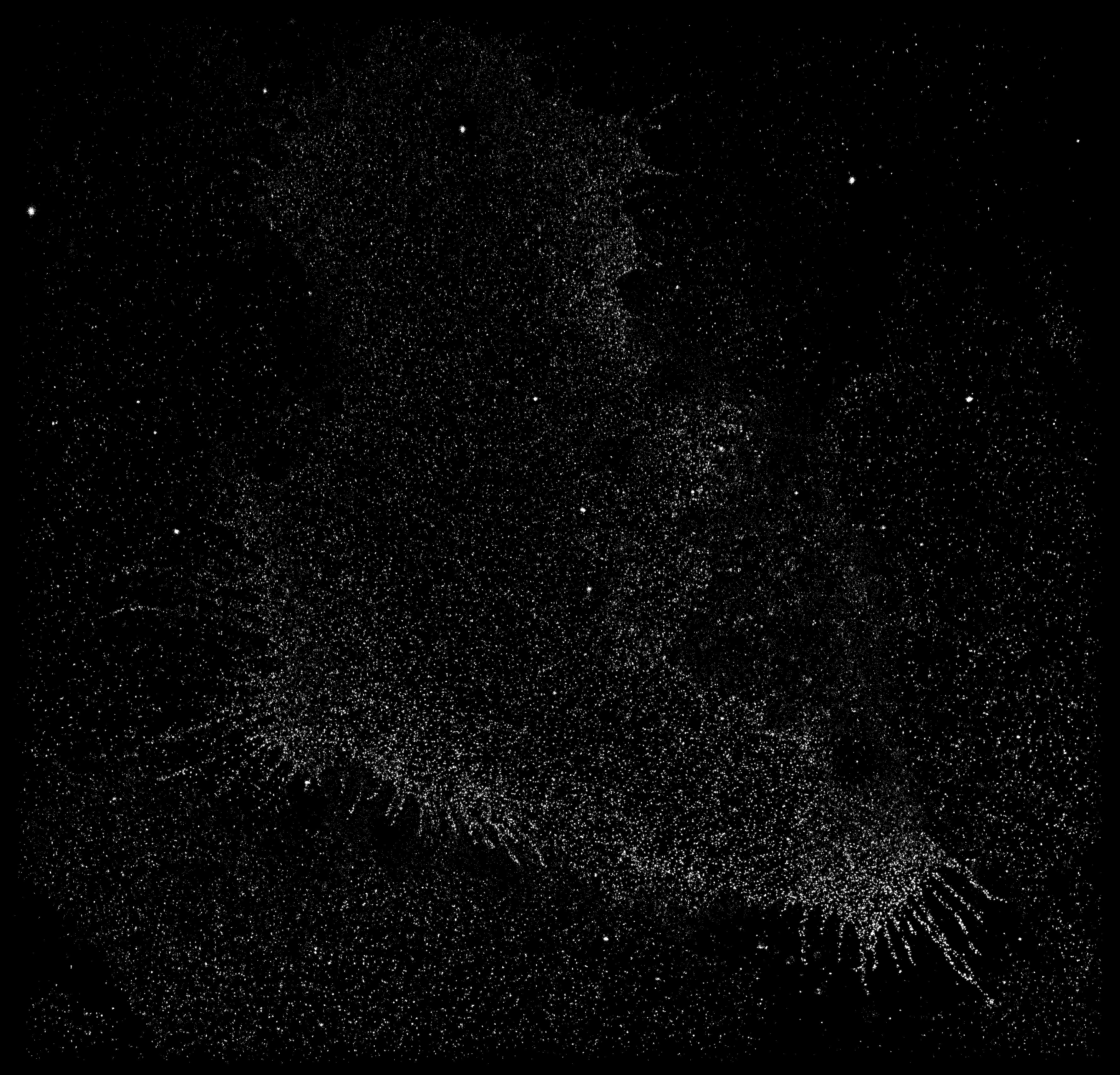

Supplement: Figure 6—source data 1. [file elife-97017-fig6-data1.zip › Figure 6_source data 1/WT.jpg]

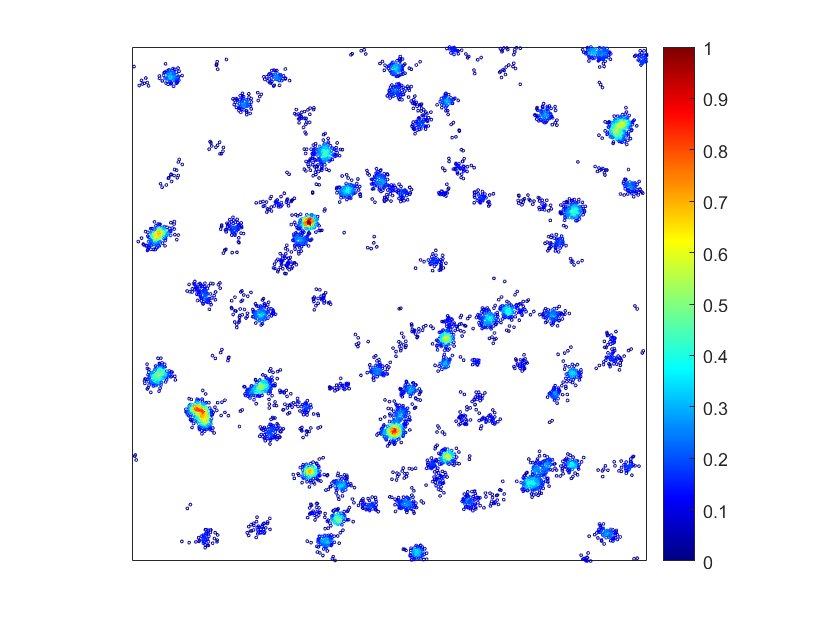

Supplement: Figure 6—source data 4. [file elife-97017-fig6-data4.zip › Figure 6_source data 4/NC-DenstiyMap.jpg]

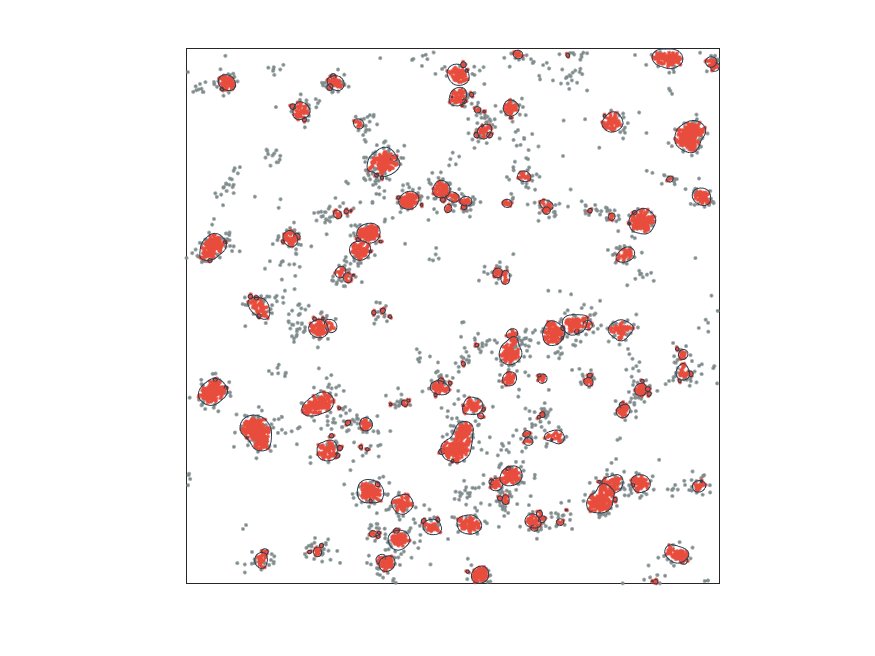

Supplement: Figure 6—source data 4. [file elife-97017-fig6-data4.zip › Figure 6_source data 4/NC-ClusterMap.jpg]

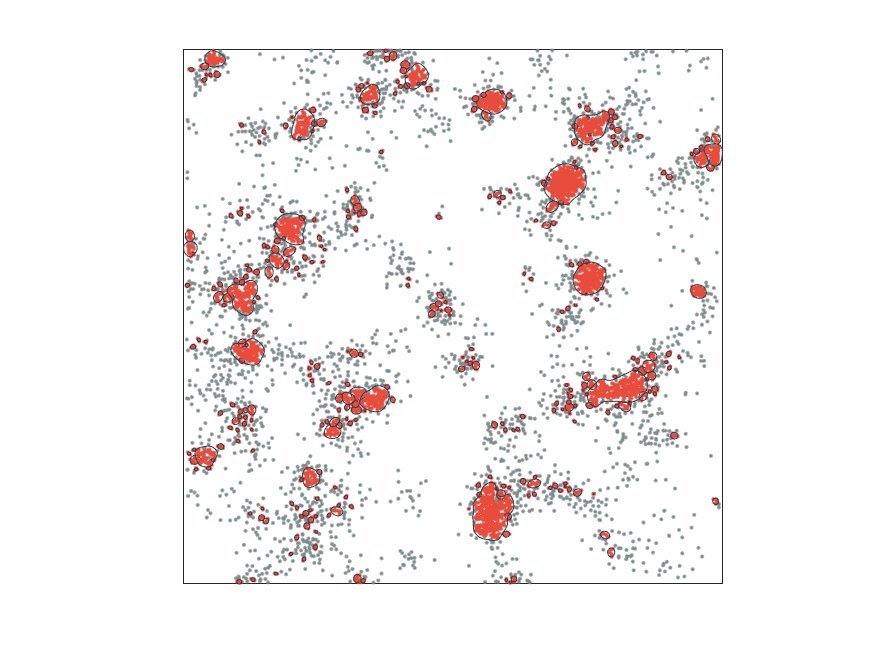

Supplement: Figure 6—source data 4. [file elife-97017-fig6-data4.zip › Figure 6_source data 4/Pitstop2-ClusterMap.jpg]

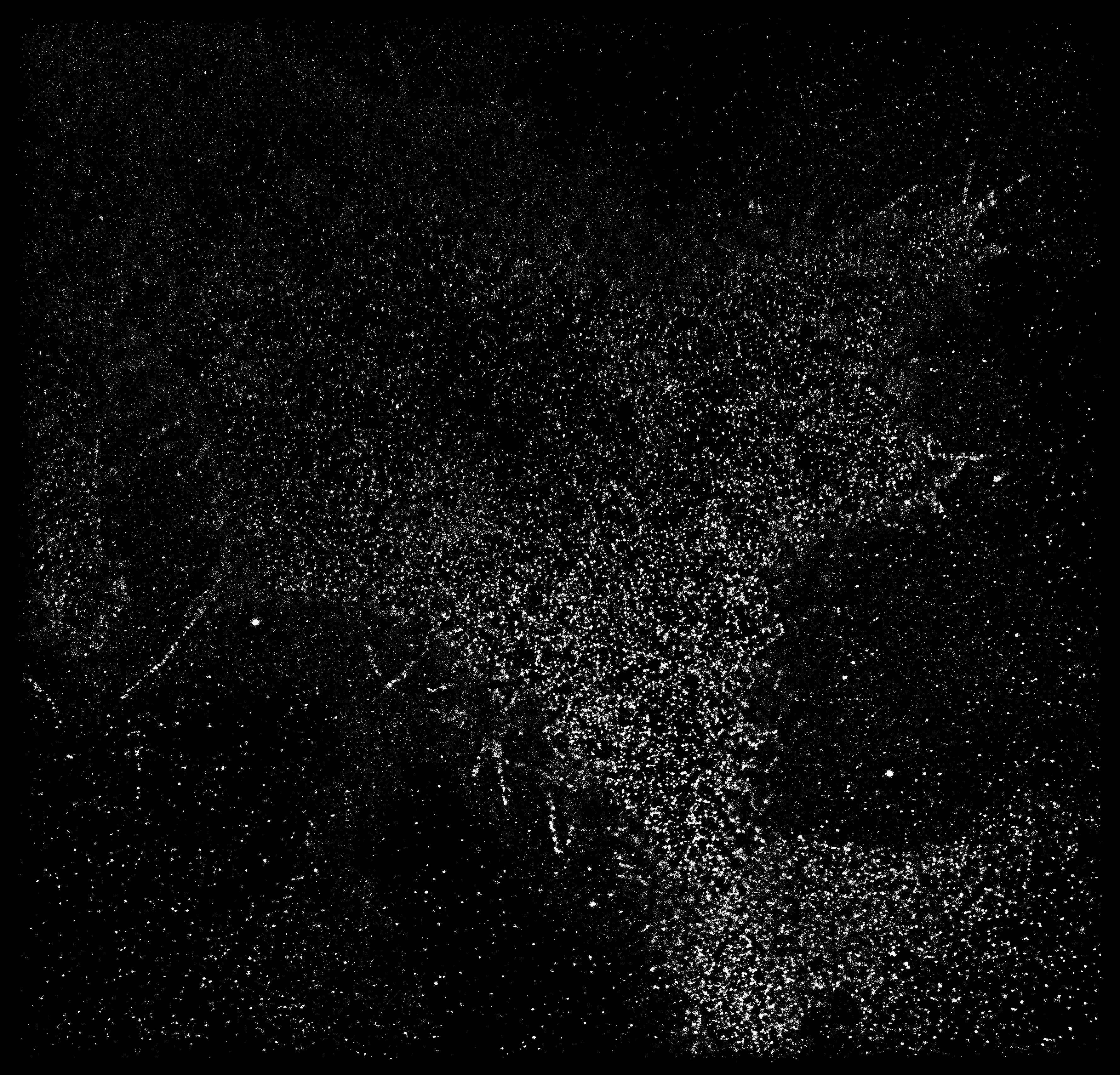

Supplement: Figure 6—source data 4. [file elife-97017-fig6-data4.zip › Figure 6_source data 4/Pitstop2.jpg]

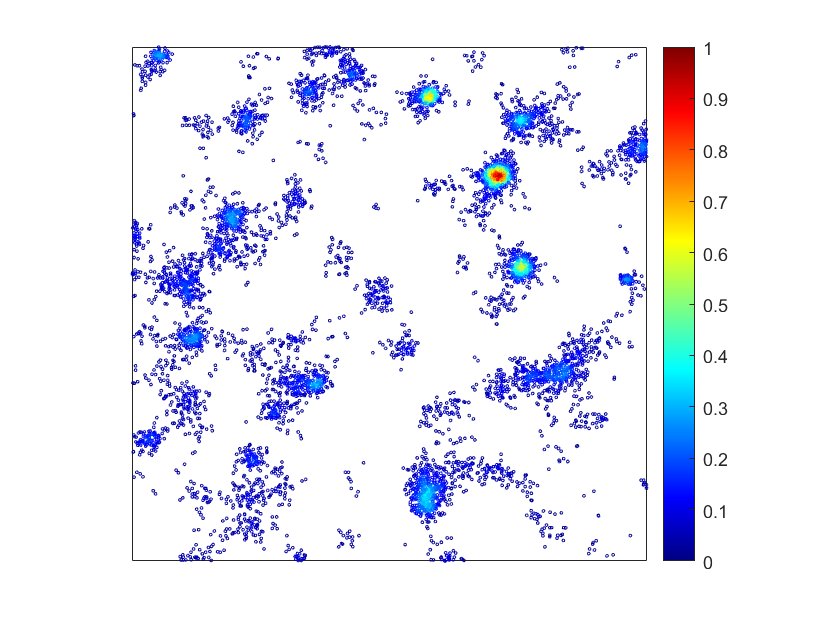

Supplement: Figure 6—source data 4. [file elife-97017-fig6-data4.zip › Figure 6_source data 4/Pitstop2-DensityMap.jpg]

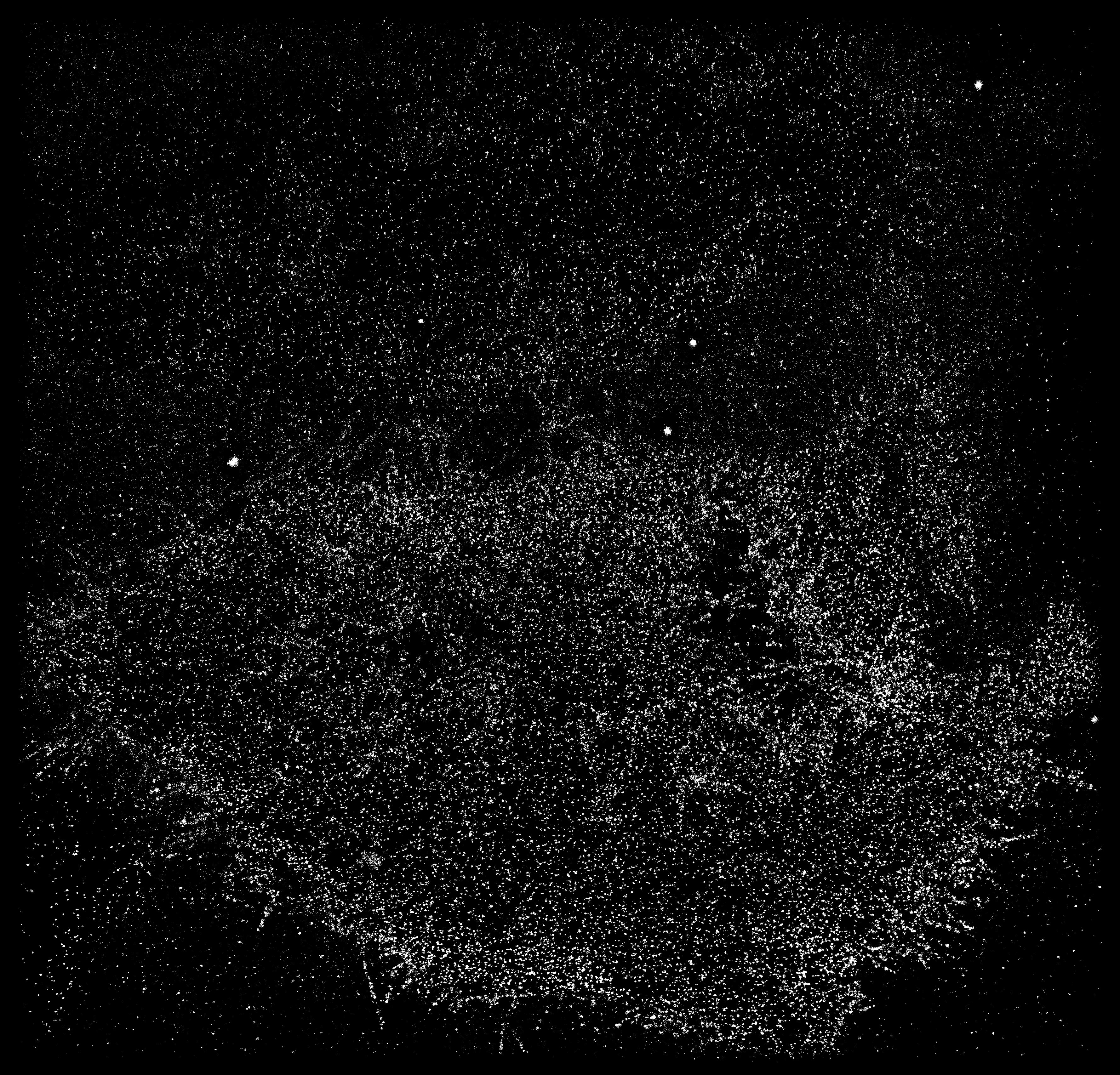

Supplement: Figure 6—source data 4. [file elife-97017-fig6-data4.zip › Figure 6_source data 4/NC.jpg]

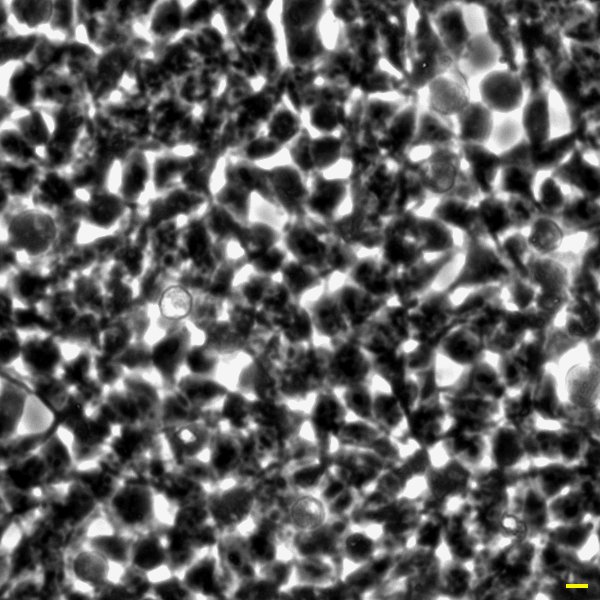

Supplement: Figure 6—figure supplement 1—source data 3. [file elife-97017-fig6-figsupp1-data3.zip › Figure 6-figure supplement 1_source data 3/YA.jpg]

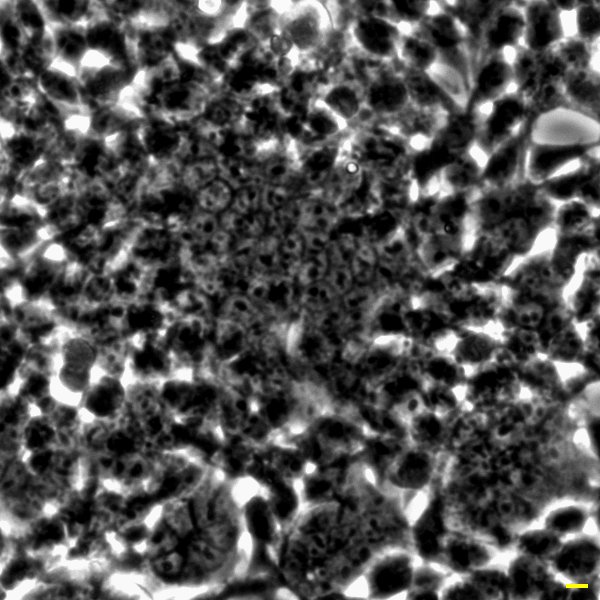

Supplement: Figure 6—figure supplement 1—source data 3. [file elife-97017-fig6-figsupp1-data3.zip › Figure 6-figure supplement 1_source data 3/WT.jpg]

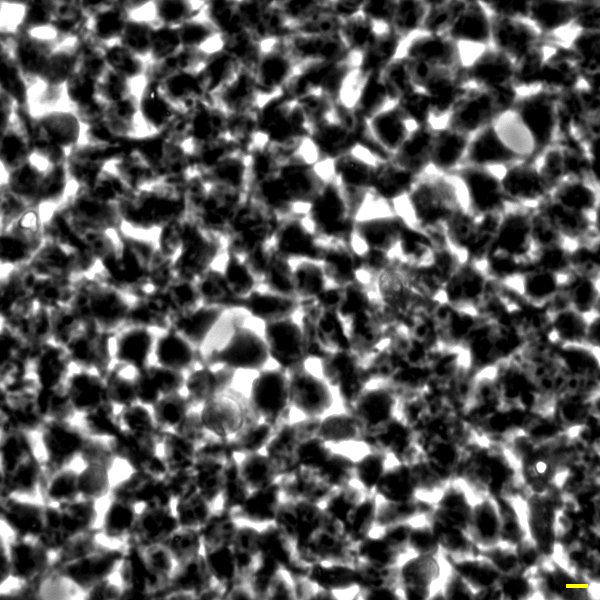

Supplement: Figure 6—figure supplement 1—source data 3. [file elife-97017-fig6-figsupp1-data3.zip › Figure 6-figure supplement 1_source data 3/NC.jpg]

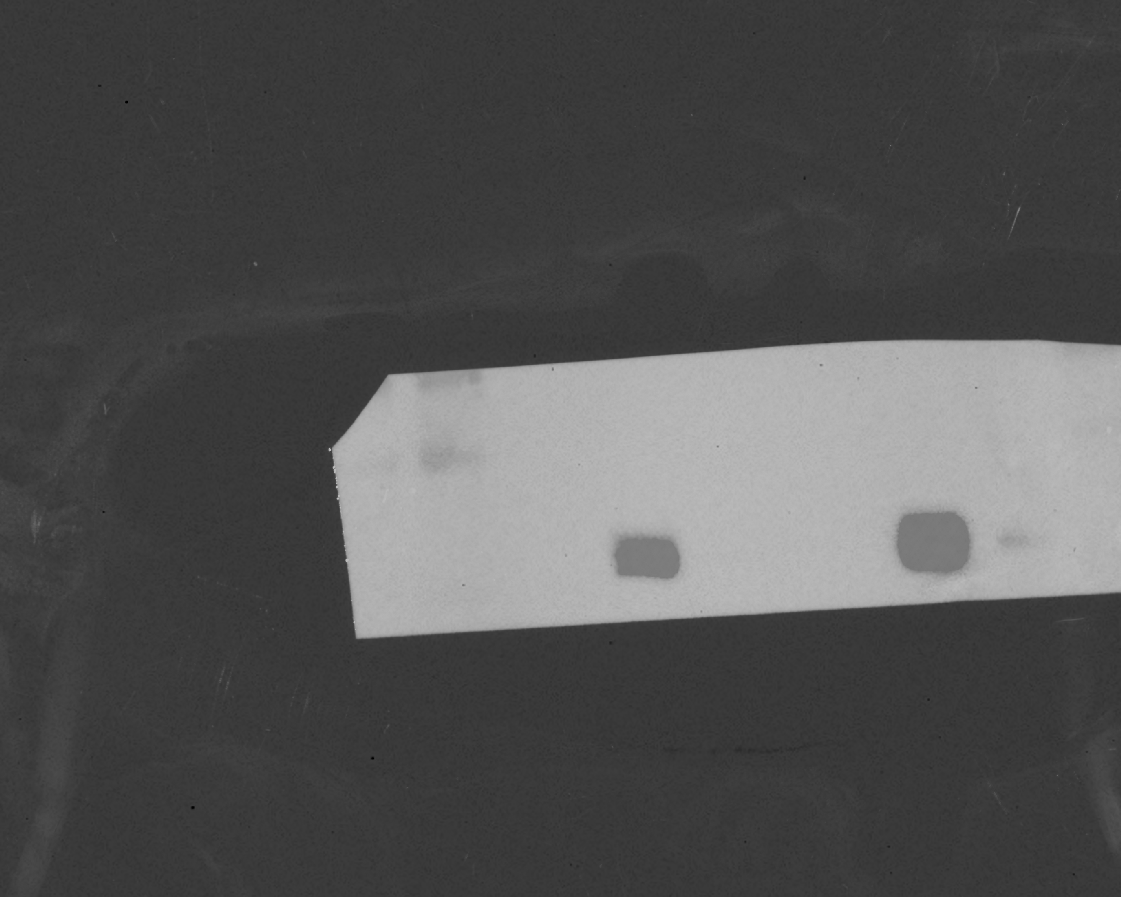

Supplement: Figure 6—figure supplement 1—source data 8. [file elife-97017-fig6-figsupp1-data8.zip › Figure 6-figure supplement 1_source data 8/F2_IP.tif]

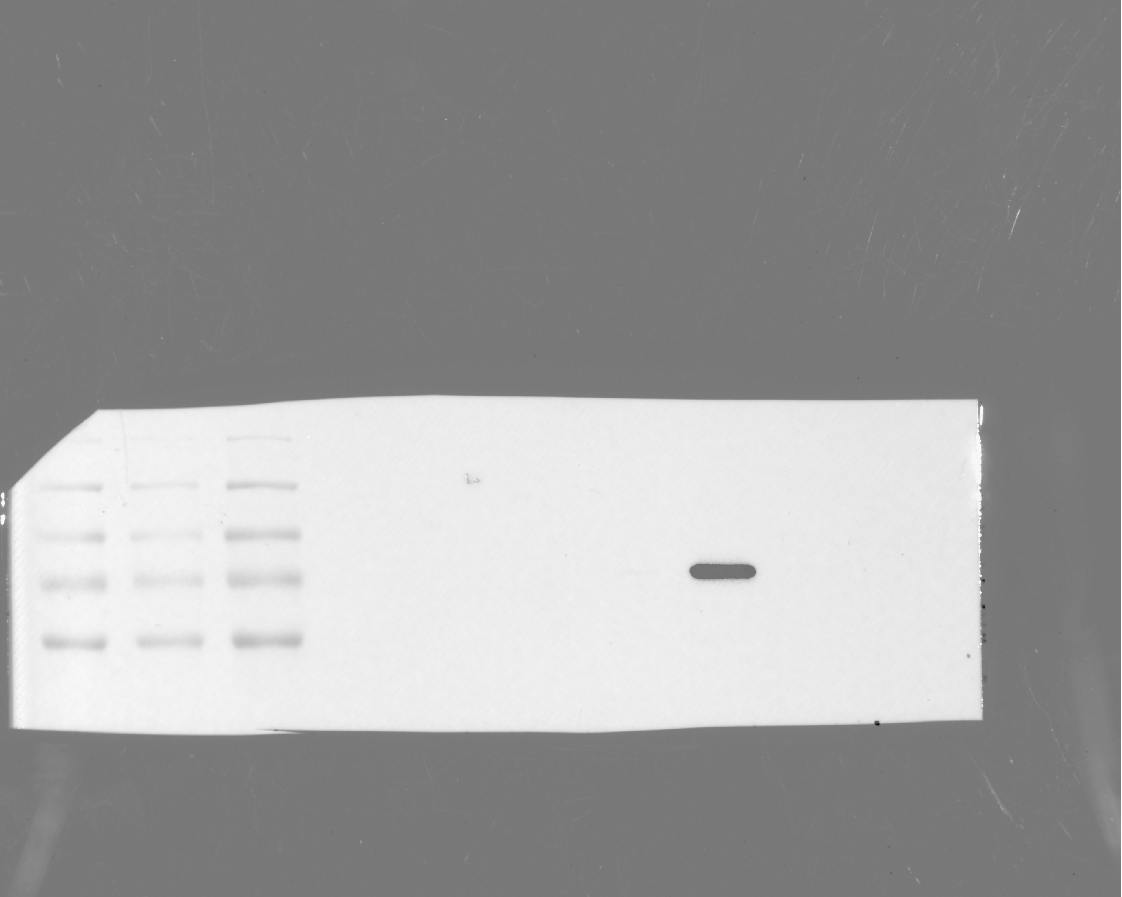

Supplement: Figure 6—figure supplement 1—source data 8. [file elife-97017-fig6-figsupp1-data8.zip › Figure 6-figure supplement 1_source data 8/AP-2_IP.tif]

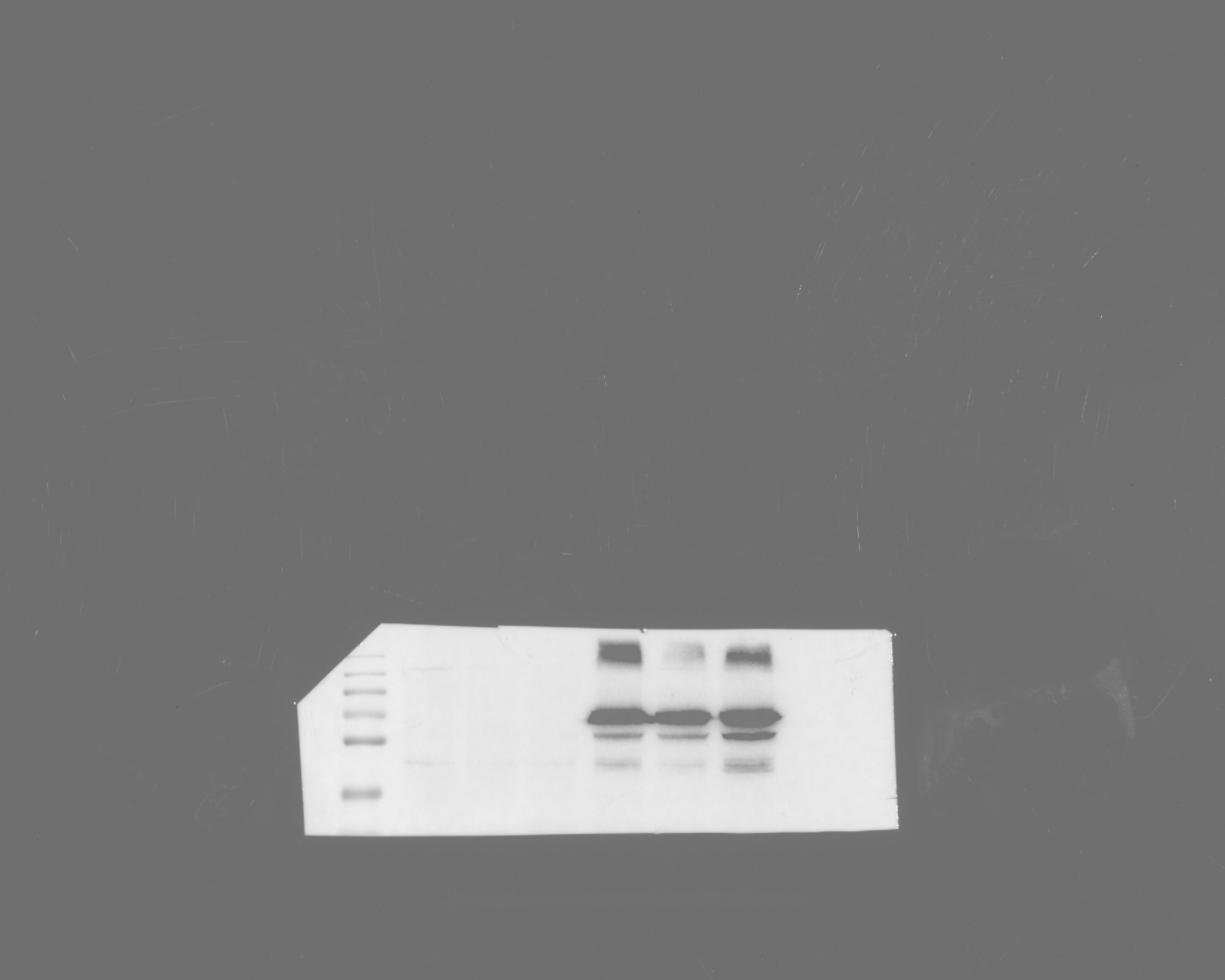

Supplement: Figure 6—figure supplement 1—source data 8. [file elife-97017-fig6-figsupp1-data8.zip › Figure 6-figure supplement 1_source data 8/AP-2_lysate.tif]

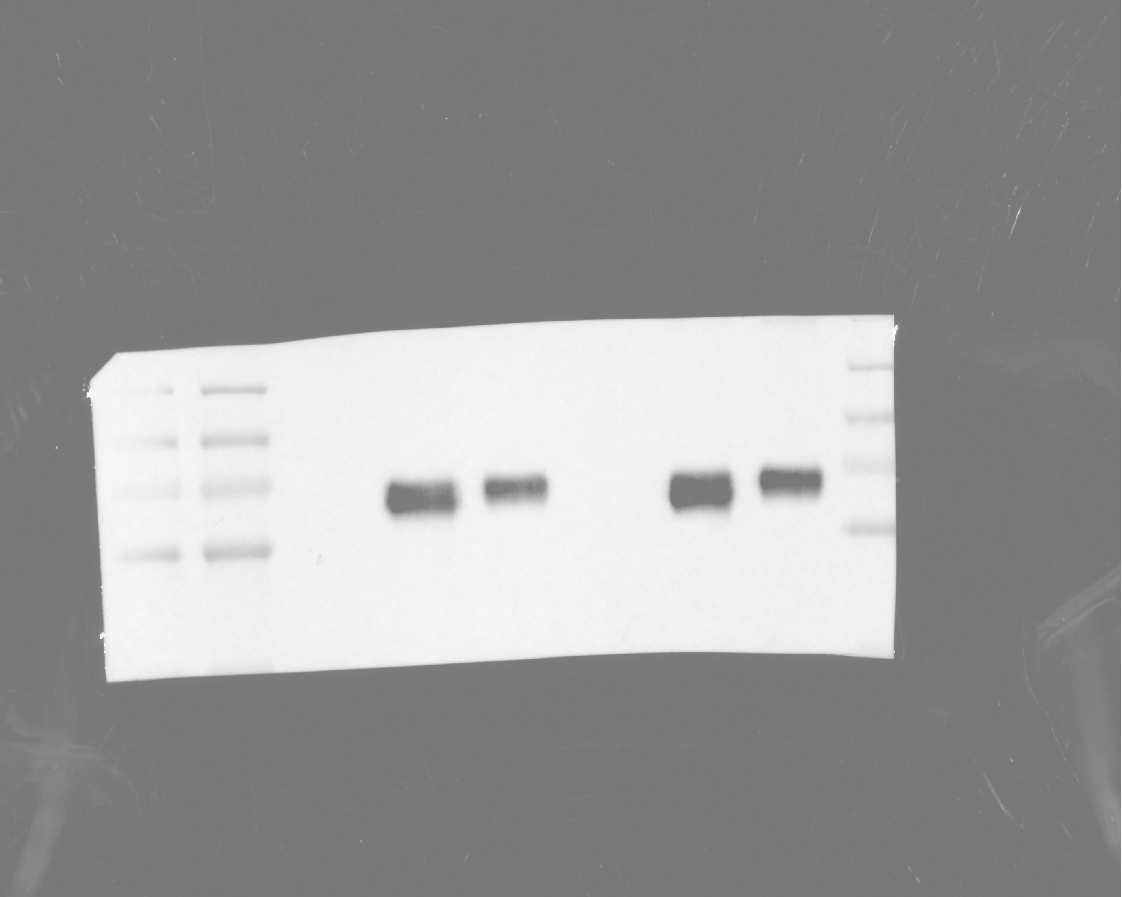

Supplement: Figure 6—figure supplement 1—source data 8. [file elife-97017-fig6-figsupp1-data8.zip › Figure 6-figure supplement 1_source data 8/F0-IP.tif]

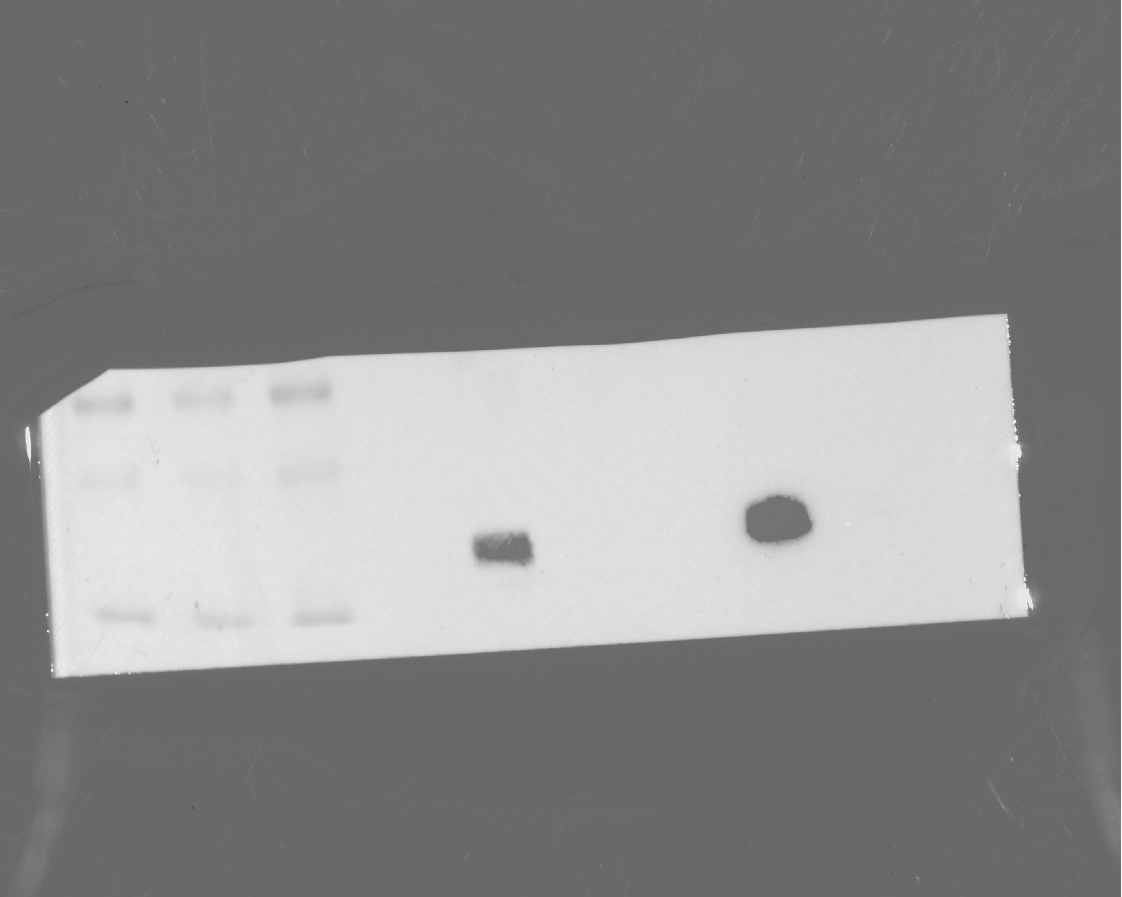

Supplement: Figure 6—figure supplement 1—source data 8. [file elife-97017-fig6-figsupp1-data8.zip › Figure 6-figure supplement 1_source data 8/F2_Iysate.tif]

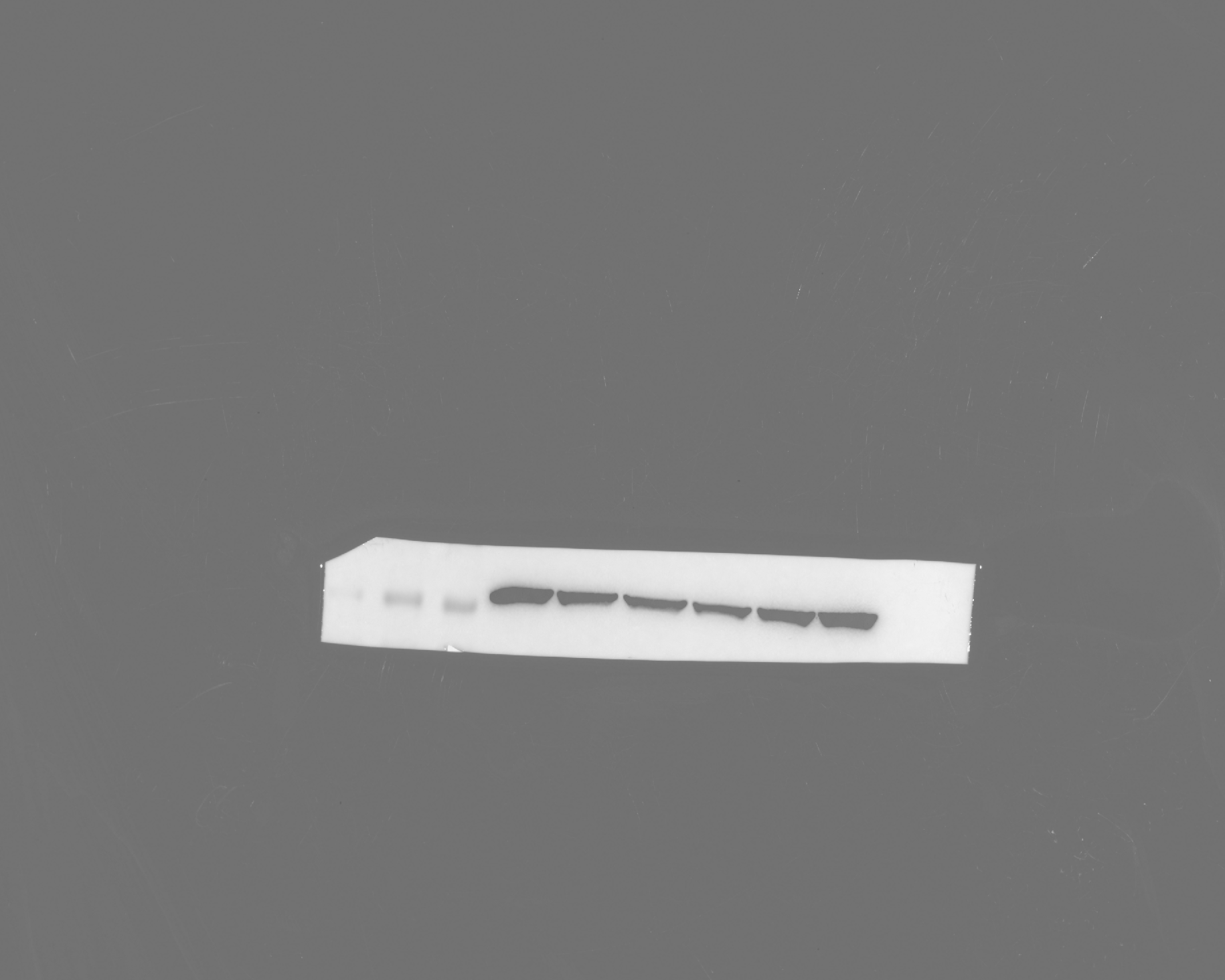

Supplement: Figure 6—figure supplement 1—source data 8. [file elife-97017-fig6-figsupp1-data8.zip › Figure 6-figure supplement 1_source data 8/GAPDH_Iysate.tif]

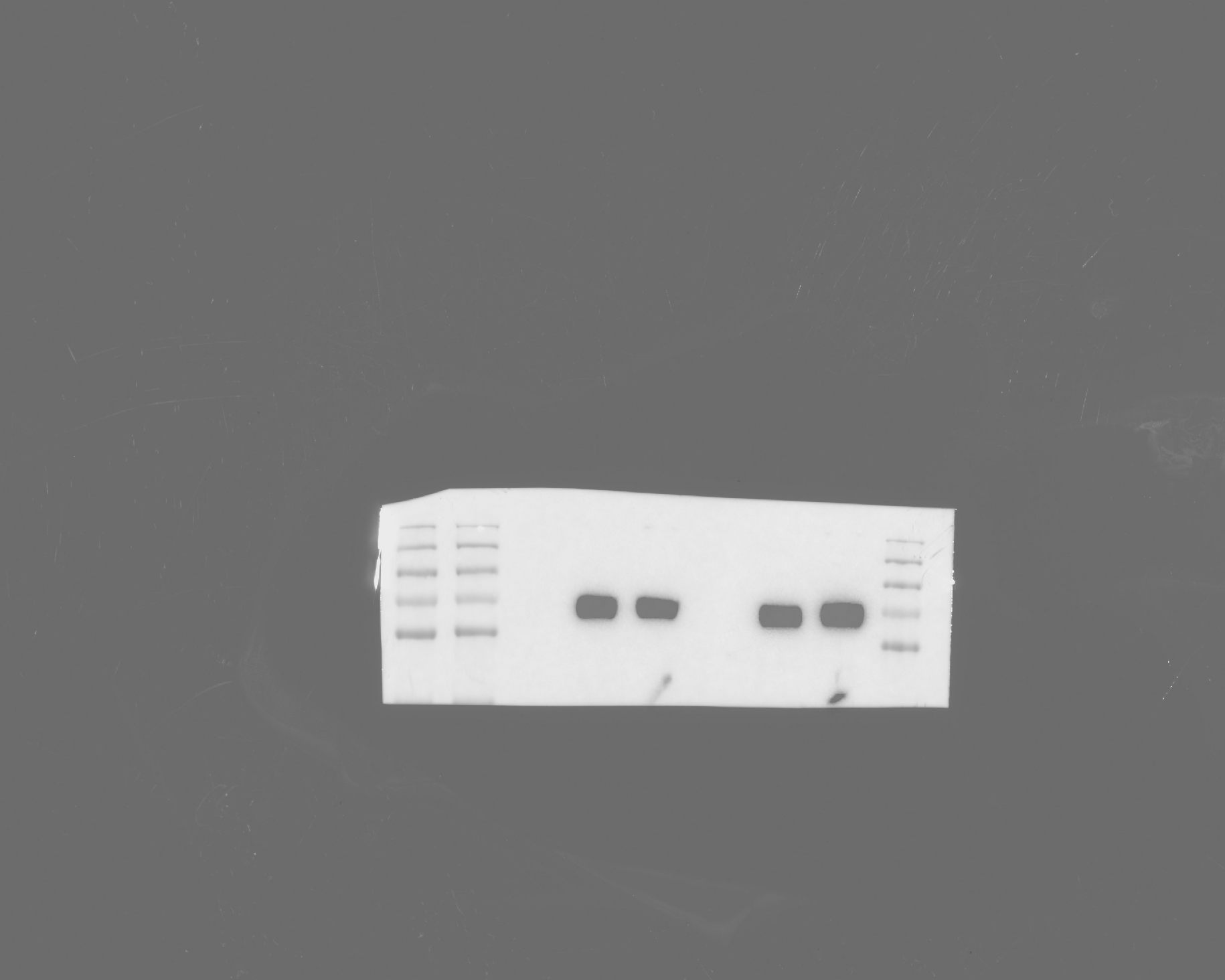

Supplement: Figure 6—figure supplement 1—source data 8. [file elife-97017-fig6-figsupp1-data8.zip › Figure 6-figure supplement 1_source data 8/F0_Iysate.tif]
