# Supplementary material for: The nanoscale organization of the Nipah virus fusion protein informs new membrane fusion mechanisms
Source: eLife. 2025 Jan 2;13:RP97017. doi: 10.7554/eLife.97017 (PMC11695058; doi:10.7554/eLife.97017)
Supplement: Figure 3—figure supplement 1—source data 5. — PPTX files indicating the relevant bands and treatments. [file elife-97017-fig3-figsupp1-data5.pptx]

## Slide 1
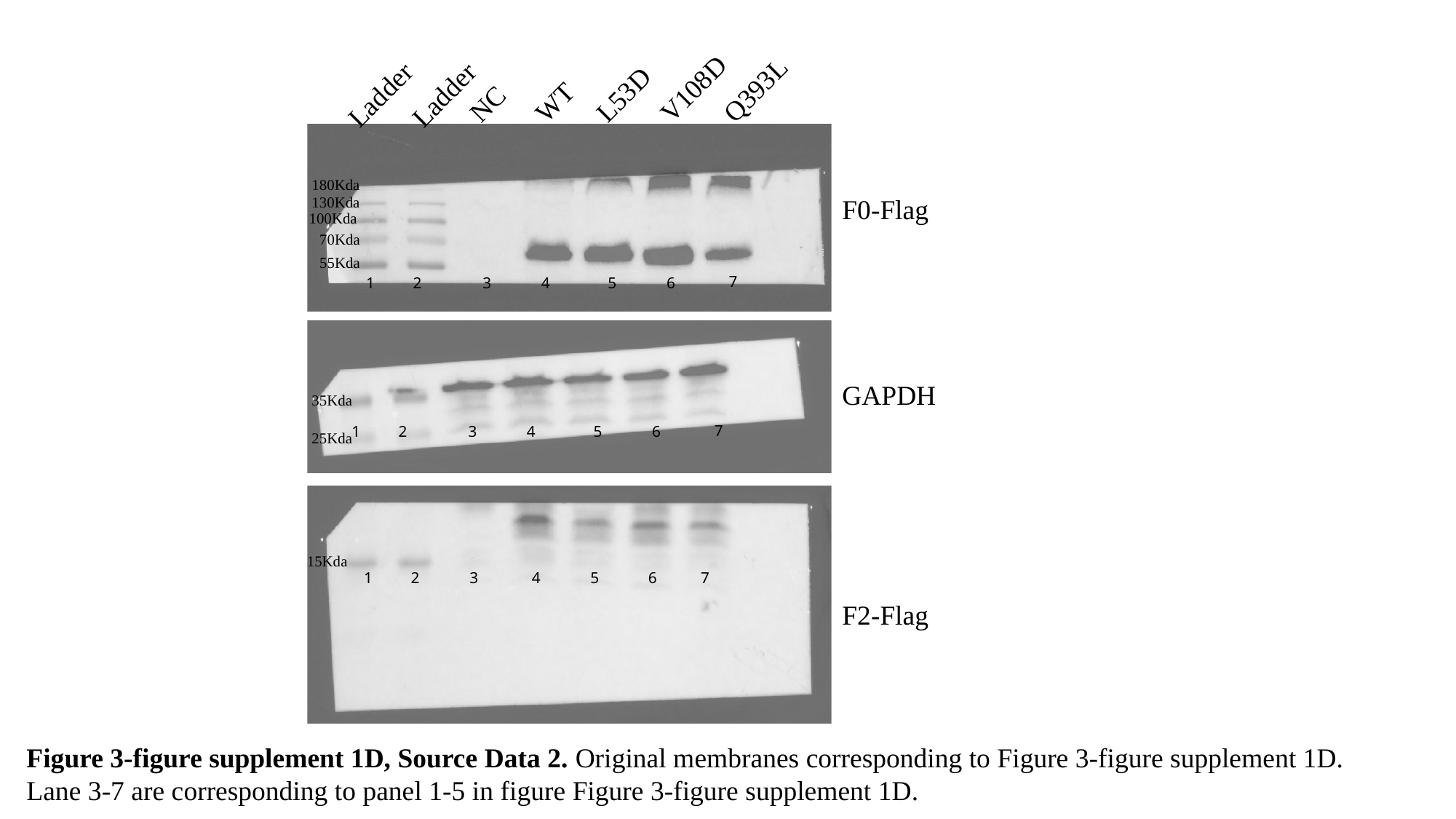

NC
WT
L53D
V108D
Q393L
Ladder
Ladder
180Kda
130Kda
100Kda
70Kda
55Kda
35Kda
25Kda
15Kda
F0-Flag
7
1
2
3
4
5
6
GAPDH
7
1
2
3
4
5
6
1
2
3
4
5
6
7
F2-Flag
Figure 3-figure supplement 1D, Source Data 2. Original membranes corresponding to Figure 3-figure supplement 1D. Lane 3-7 are corresponding to panel 1-5 in figure Figure 3-figure supplement 1D.
