# Supplementary material for: The nanoscale organization of the Nipah virus fusion protein informs new membrane fusion mechanisms
Source: eLife. 2025 Jan 2;13:RP97017. doi: 10.7554/eLife.97017 (PMC11695058; doi:10.7554/eLife.97017)
Supplement: Figure 3—figure supplement 1—source data 8. — PPTX files indicating the relevant bands and treatments. [file elife-97017-fig3-figsupp1-data8.pptx]

## Slide 1
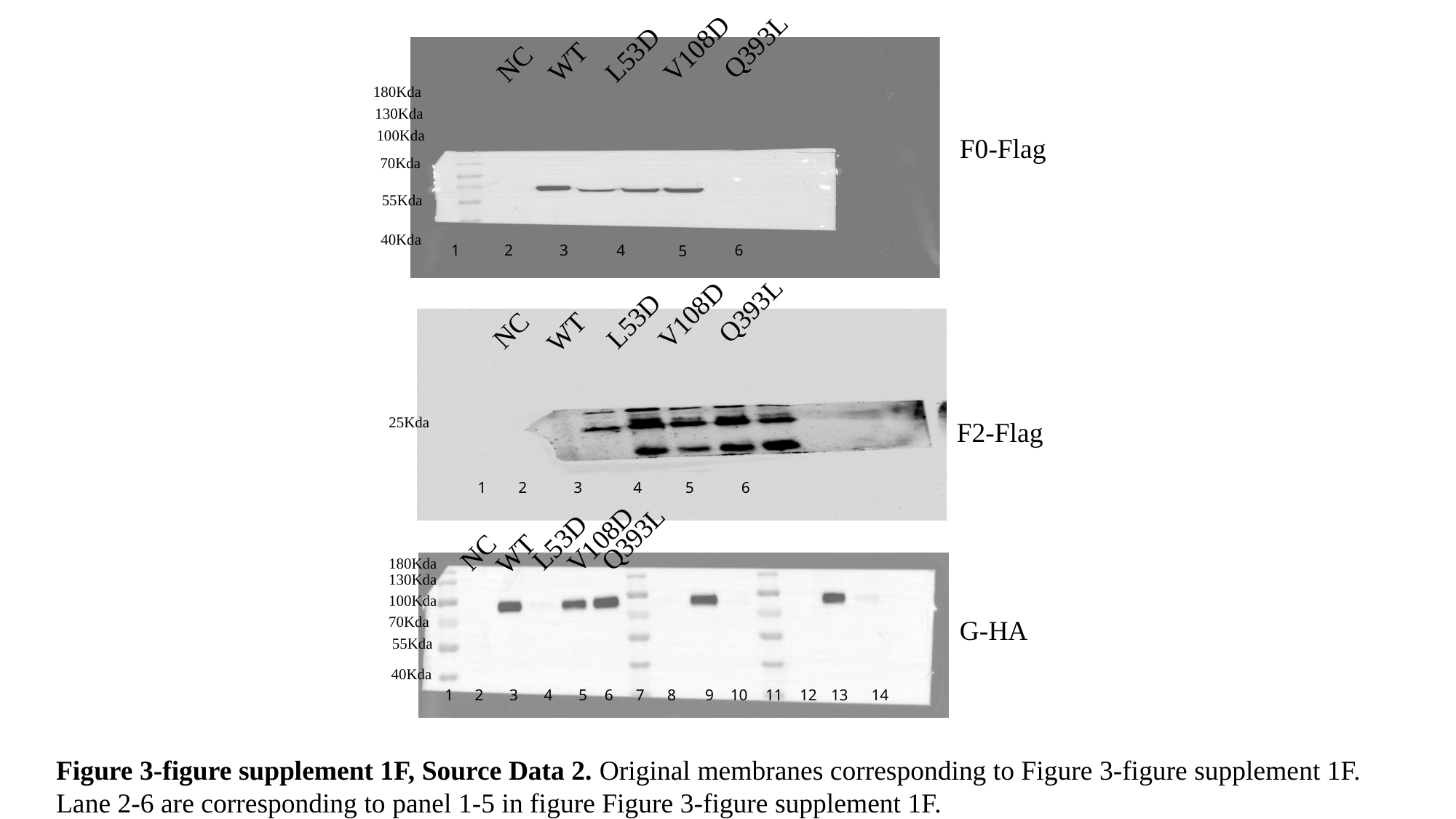

Q393L
NC
WT
L53D
V108D
180Kda
130Kda
100Kda
F0-Flag
70Kda
55Kda
40Kda
1
2
3
4
6
5
Q393L
NC
L53D
V108D
WT
25Kda
F2-Flag
1
2
3
4
5
6
L53D
NC
Q393L
V108D
WT
180Kda
130Kda
100Kda
70Kda
G-HA
55Kda
40Kda
1
2
3
4
5
6
7
8
9
10
11
12
13
14
Figure 3-figure supplement 1F, Source Data 2. Original membranes corresponding to Figure 3-figure supplement 1F. Lane 2-6 are corresponding to panel 1-5 in figure Figure 3-figure supplement 1F.
