# Supplementary material for: The nanoscale organization of the Nipah virus fusion protein informs new membrane fusion mechanisms
Source: eLife. 2025 Jan 2;13:RP97017. doi: 10.7554/eLife.97017 (PMC11695058; doi:10.7554/eLife.97017)
Supplement: Figure 3—figure supplement 1—source data 11. — PPTX files indicating the relevant bands and treatments. [file elife-97017-fig3-figsupp1-data11.pptx]

## Slide 1
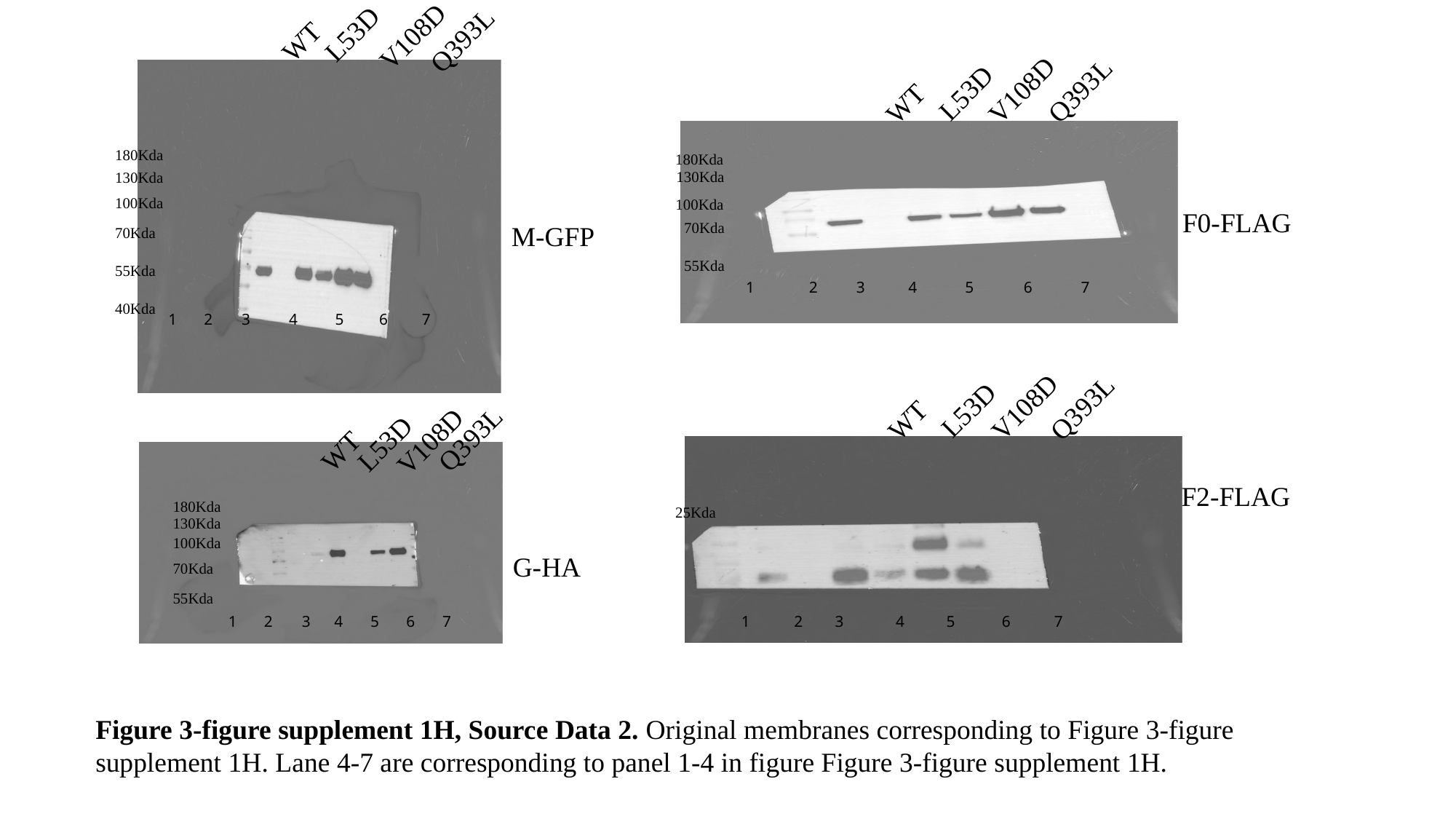

WT
L53D
V108D
Q393L
L53D
Q393L
WT
V108D
180Kda
180Kda
130Kda
130Kda
100Kda
100Kda
F0-FLAG
70Kda
M-GFP
70Kda
55Kda
55Kda
1
2
3
4
5
6
7
40Kda
1
2
3
4
5
6
7
L53D
Q393L
WT
V108D
WT
L53D
Q393L
V108D
F2-FLAG
180Kda
25Kda
130Kda
100Kda
G-HA
70Kda
55Kda
1
2
3
4
5
6
7
1
2
3
4
5
6
7
Figure 3-figure supplement 1H, Source Data 2. Original membranes corresponding to Figure 3-figure supplement 1H. Lane 4-7 are corresponding to panel 1-4 in figure Figure 3-figure supplement 1H.
