# Supplementary material for: The nanoscale organization of the Nipah virus fusion protein informs new membrane fusion mechanisms
Source: eLife. 2025 Jan 2;13:RP97017. doi: 10.7554/eLife.97017 (PMC11695058; doi:10.7554/eLife.97017)
Supplement: Figure 4—source data 2. — PPTX files indicating the relevant bands and treatments. [file elife-97017-fig4-data2.pptx]

## Slide 1
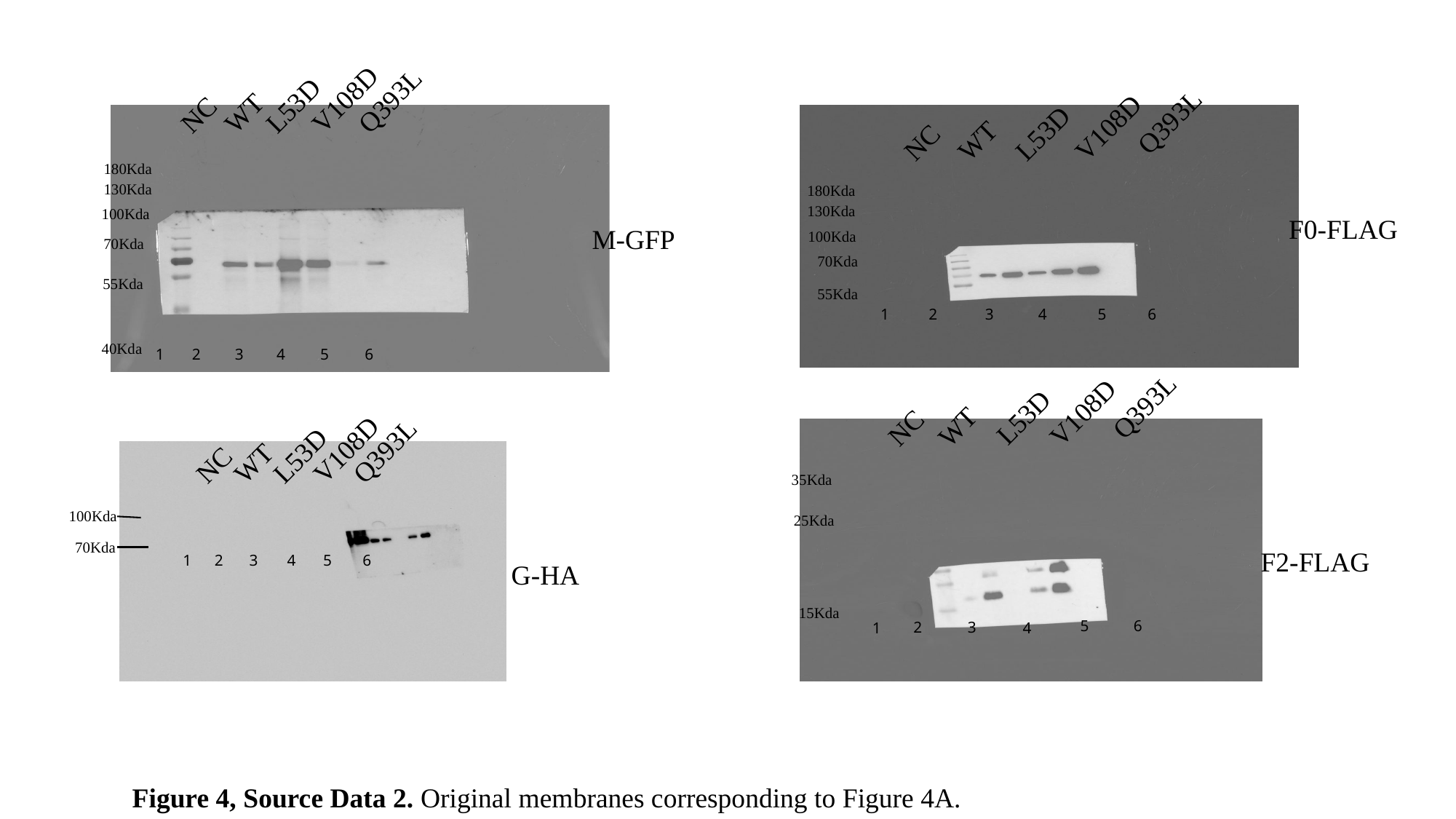

Q393L
NC
WT
L53D
V108D
Q393L
NC
WT
L53D
V108D
180Kda
130Kda
180Kda
130Kda
100Kda
F0-FLAG
M-GFP
100Kda
70Kda
70Kda
55Kda
55Kda
1
2
3
4
5
6
40Kda
1
2
3
4
5
6
Q393L
L53D
V108D
NC
WT
Q393L
NC
WT
L53D
V108D
35Kda
100Kda
25Kda
70Kda
F2-FLAG
1
2
3
4
5
6
G-HA
15Kda
5
6
2
3
1
4
Figure 4, Source Data 2. Original membranes corresponding to Figure 4A.
