# Supplementary material for: The nanoscale organization of the Nipah virus fusion protein informs new membrane fusion mechanisms
Source: eLife. 2025 Jan 2;13:RP97017. doi: 10.7554/eLife.97017 (PMC11695058; doi:10.7554/eLife.97017)
Supplement: Figure 5—figure supplement 1—source data 2. — PPTX files indicating the relevant bands and treatments. [file elife-97017-fig5-figsupp1-data2.pptx]

## Slide 1
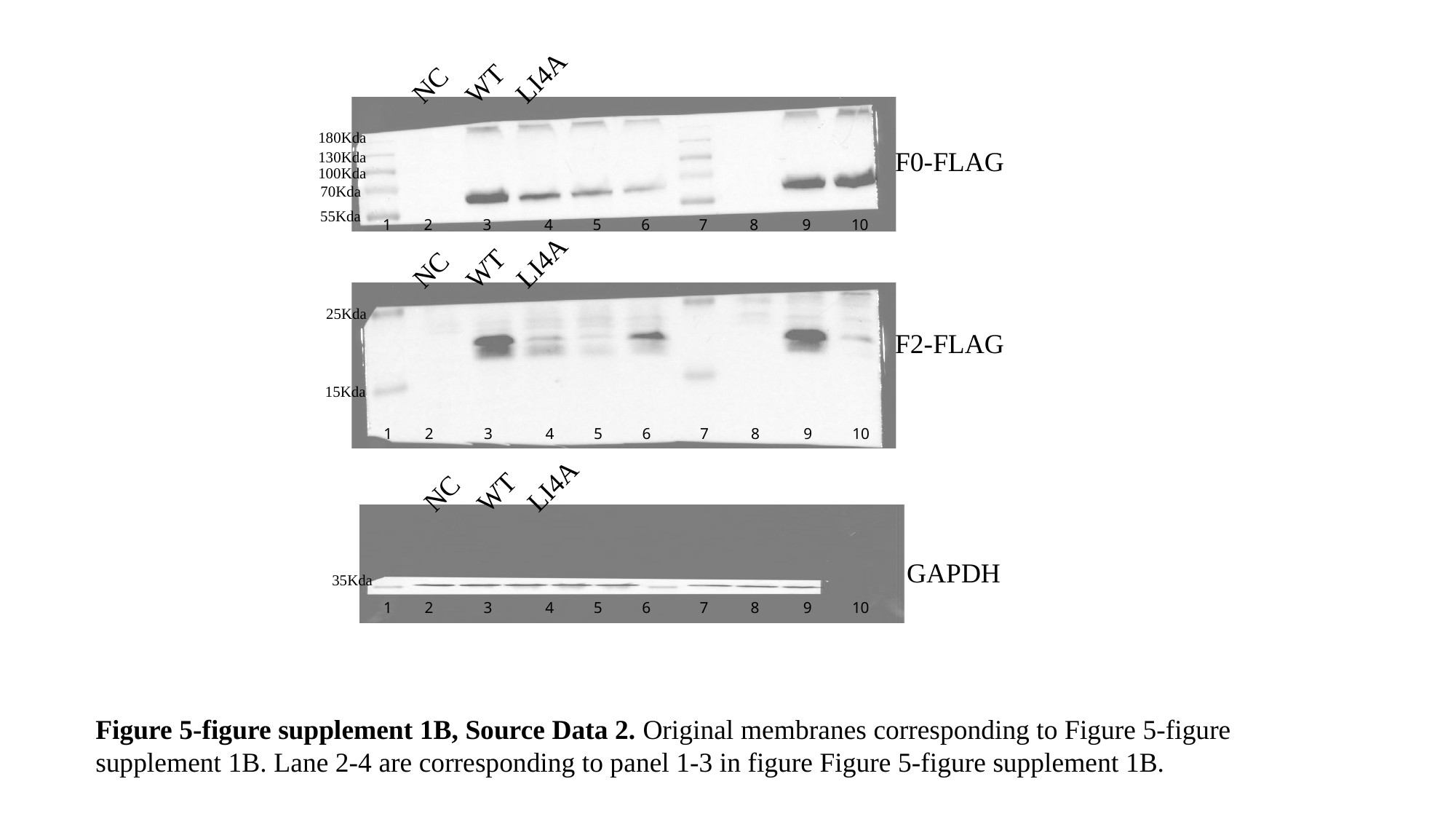

NC
LI4A
WT
180Kda
F0-FLAG
130Kda
100Kda
70Kda
55Kda
1
2
3
4
5
6
7
8
9
10
NC
LI4A
WT
25Kda
F2-FLAG
15Kda
1
2
3
4
5
6
7
8
9
10
NC
LI4A
WT
GAPDH
35Kda
1
2
3
4
5
6
7
8
9
10
Figure 5-figure supplement 1B, Source Data 2. Original membranes corresponding to Figure 5-figure supplement 1B. Lane 2-4 are corresponding to panel 1-3 in figure Figure 5-figure supplement 1B.
