# Supplementary material for: The nanoscale organization of the Nipah virus fusion protein informs new membrane fusion mechanisms
Source: eLife. 2025 Jan 2;13:RP97017. doi: 10.7554/eLife.97017 (PMC11695058; doi:10.7554/eLife.97017)
Supplement: Figure 5—figure supplement 1—source data 6. — PPTX files indicating the relevant bands and treatments. [file elife-97017-fig5-figsupp1-data6.pptx]

## Slide 1
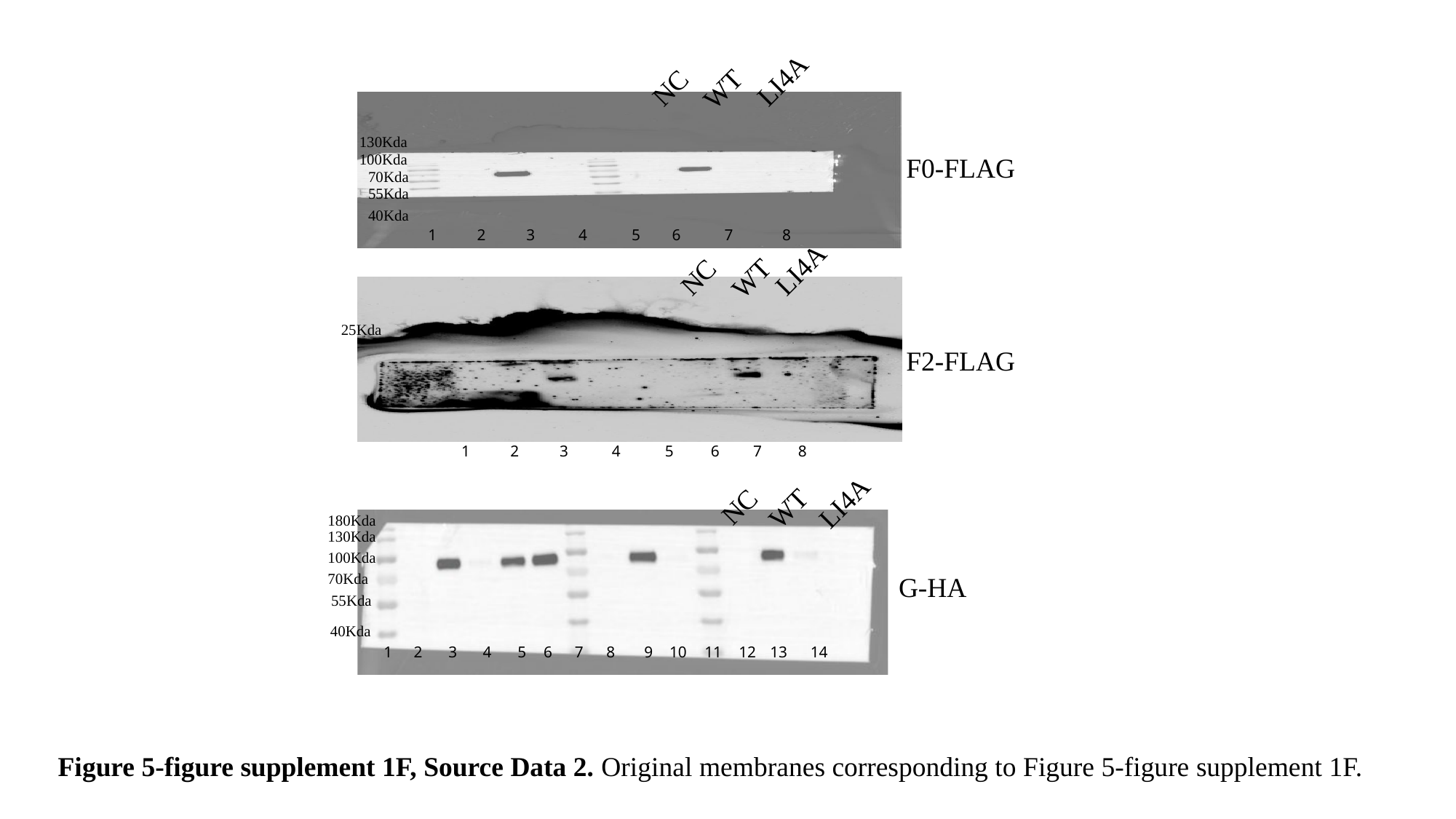

NC
LI4A
WT
130Kda
100Kda
F0-FLAG
70Kda
55Kda
40Kda
1
2
3
4
5
6
7
8
NC
LI4A
WT
25Kda
F2-FLAG
1
2
3
4
5
6
7
8
NC
WT
LI4A
180Kda
130Kda
100Kda
70Kda
G-HA
55Kda
40Kda
1
2
3
4
5
6
7
8
9
10
11
12
13
14
Figure 5-figure supplement 1F, Source Data 2. Original membranes corresponding to Figure 5-figure supplement 1F.
