# Supplementary material for: The nanoscale organization of the Nipah virus fusion protein informs new membrane fusion mechanisms
Source: eLife. 2025 Jan 2;13:RP97017. doi: 10.7554/eLife.97017 (PMC11695058; doi:10.7554/eLife.97017)
Supplement: Figure 6—figure supplement 1—source data 2. — PPTX files indicating the relevant bands and treatments. [file elife-97017-fig6-figsupp1-data2.pptx]

## Slide 1
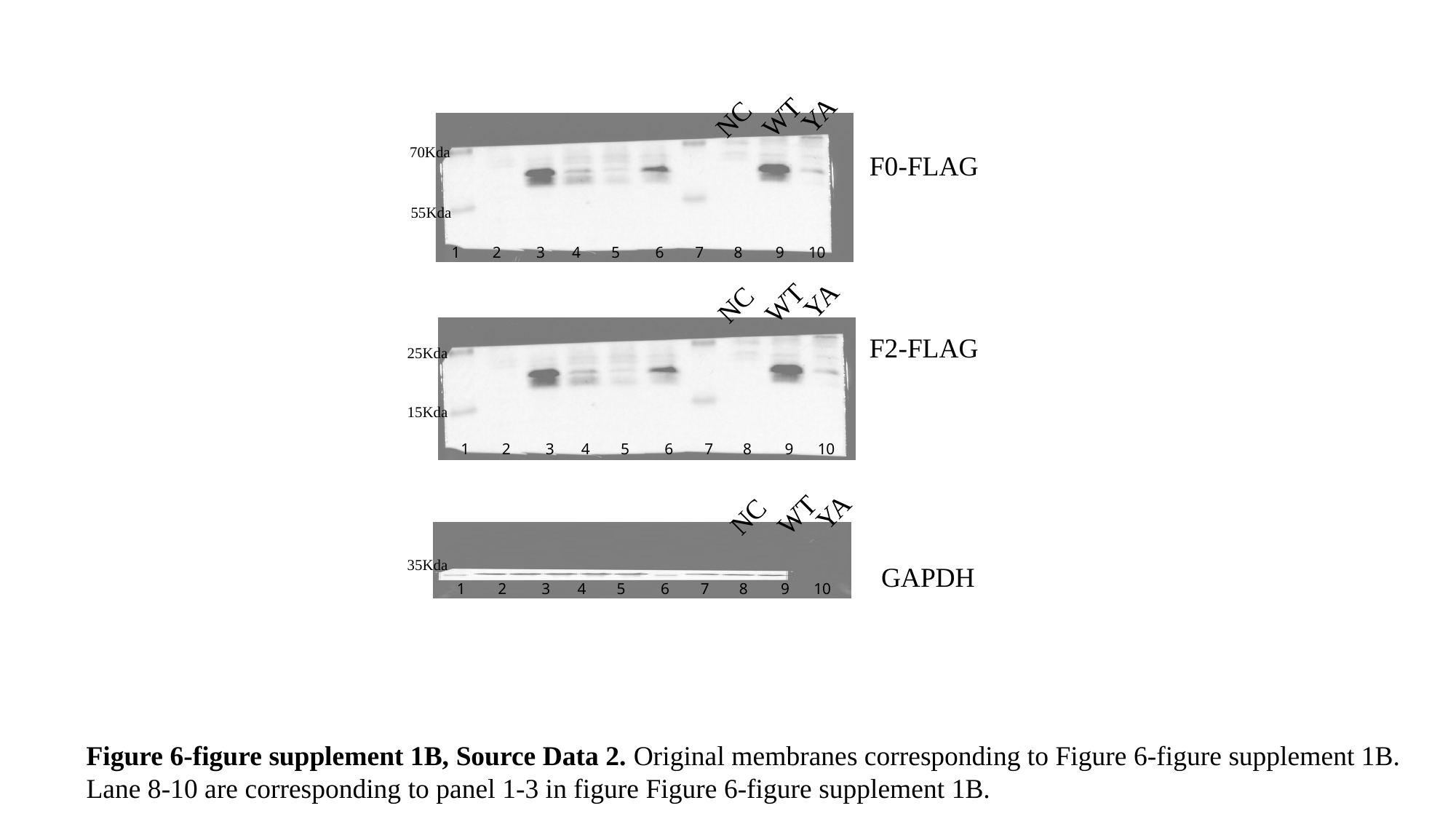

YA
NC
WT
70Kda
F0-FLAG
55Kda
1
2
3
4
5
6
7
8
9
10
YA
NC
WT
F2-FLAG
25Kda
15Kda
1
2
3
4
5
6
7
8
9
10
YA
NC
WT
35Kda
GAPDH
1
2
3
4
5
6
7
8
9
10
Figure 6-figure supplement 1B, Source Data 2. Original membranes corresponding to Figure 6-figure supplement 1B. Lane 8-10 are corresponding to panel 1-3 in figure Figure 6-figure supplement 1B.
