# Supplementary material for: The nanoscale organization of the Nipah virus fusion protein informs new membrane fusion mechanisms
Source: eLife. 2025 Jan 2;13:RP97017. doi: 10.7554/eLife.97017 (PMC11695058; doi:10.7554/eLife.97017)
Supplement: Figure 6—figure supplement 1—source data 9. [file elife-97017-fig6-figsupp1-data9.pptx]

## Slide 1
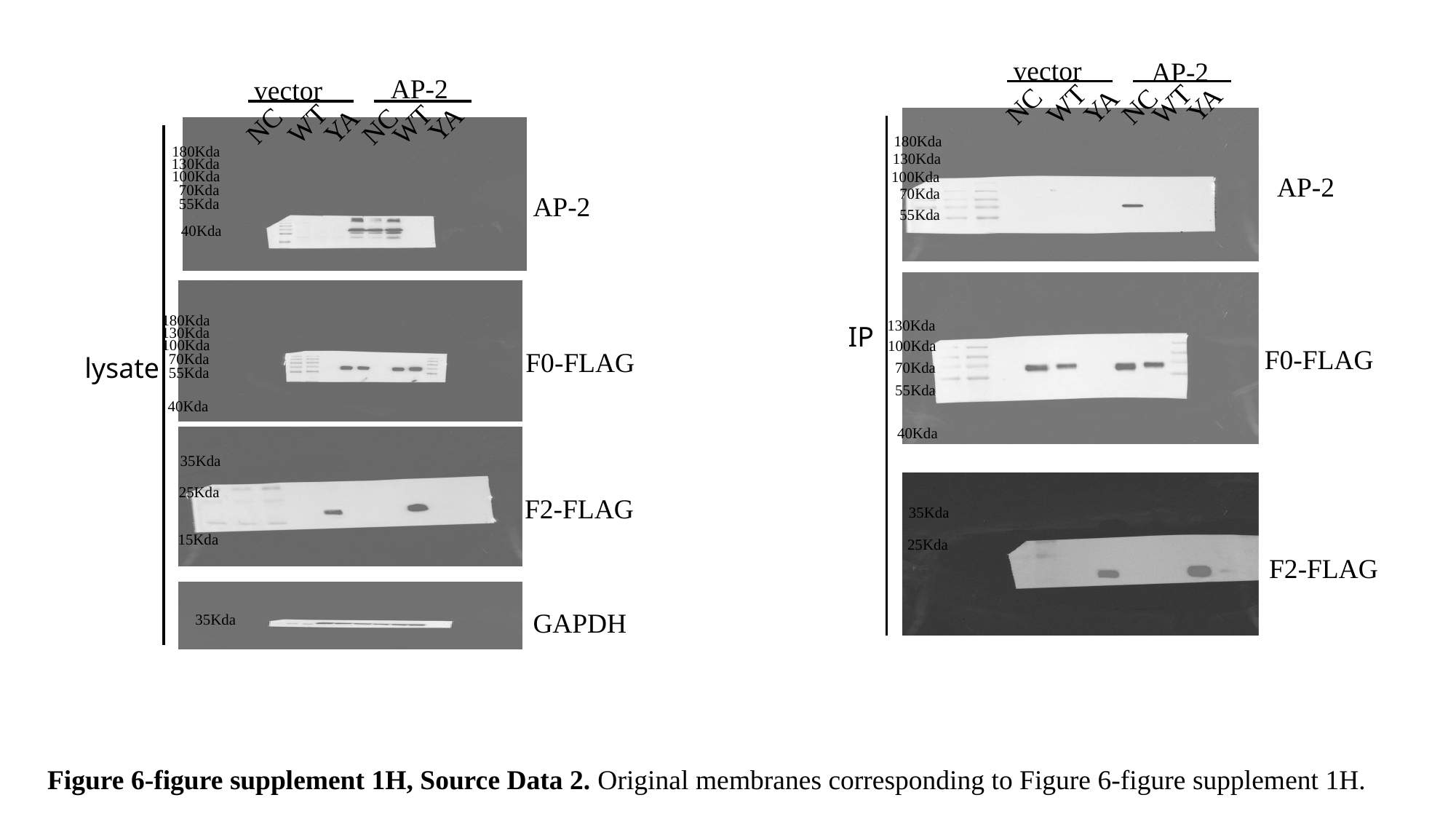

vector
AP-2
YA
AP-2
YA
NC
WT
NC
WT
vector
YA
YA
NC
WT
NC
WT
180Kda
180Kda
130Kda
130Kda
100Kda
100Kda
AP-2
70Kda
70Kda
AP-2
55Kda
55Kda
40Kda
180Kda
130Kda
IP
130Kda
100Kda
100Kda
F0-FLAG
F0-FLAG
70Kda
lysate
70Kda
55Kda
55Kda
40Kda
40Kda
35Kda
25Kda
F2-FLAG
35Kda
15Kda
25Kda
F2-FLAG
GAPDH
35Kda
Figure 6-figure supplement 1H, Source Data 2. Original membranes corresponding to Figure 6-figure supplement 1H.
